# Visible Light-Induced Templated Metathesis of Peptide–Nucleic Acid Conjugates with a Diselenide Bridge

**DOI:** 10.3390/biom13111676

**Published:** 2023-11-20

**Authors:** Mateusz Waliczek, Wiktoria Gancarz, Paulina Pochwała, Özge Pehlivan, Piotr Stefanowicz

**Affiliations:** Faculty of Chemistry, University of Wrocław, Joliot-Curie 14, 50-383 Wrocław, Poland

**Keywords:** peptide nucleic acid, templated synthesis, selenium, metathesis

## Abstract

The use of template molecules as chemical scaffolds that significantly influence the course of the reaction has recently been intensively studied. Peptide nucleic acids (PNA) are molecules that mimic natural nucleic acids. They are a promising matrix in such reactions because they possess high affinity and specificity in their interactions. The manner of PNA interaction is predictable based on sequence complementarity. Recently, we report the visible light-induced metathesis reaction in peptides containing a diselenide bond. Herein, we present an efficient and straightforward method of the visible light-driven diselenide-based metathesis of peptide–nucleic acid conjugates. Compared to a similar photochemical transformation in peptides, a significant increase in the metathesis efficiency was obtained due to the template effect.

## 1. Introduction

Typically, the synthesis of biopolymers is performed step by step using appropriate building blocks. The advantage of this method is that the efficiency of each step can be controlled and the intermediates can often be purified. However, attempts are now being made to obtain molecules consisting of identical or similar building blocks using template synthesis. The designed templates allow for the efficient control of the reaction so that a specific product is preferentially generated from a number of other possibilities. In general, the effect of templates in chemistry is mainly related to macrocycles, which can also be tailored for biological applications [1,2], although attempts are being made to use template synthesis to obtain oligomeric systems with defined sequences [3,4]. The diversity of the reactive functional groups present in biomolecules is related to the problem of chemoselectivity. Water tolerance is also an important issue in the synthesis or chemical transformation of large biomolecules. Thus, combining high reactivity with chemoselectivity and water tolerance remains a challenge. A very helpful tool in this case is the use of template chemistry. Templates determine the pattern formation on their surface and allow for the approximation of reactive groups. As a result, the reaction proceeds at a higher effective molarity, making it possible to form products in low-concentration solutions, in which the synthesis of the desired compound would be ineffective in the absence of the matrix [5]. Several applications of this approach have been proposed, particularly in the field of peptides or peptide nucleic acids (PNAs) [6]. The latter are DNA/RNA analogs that contain, instead of a phosphoribosyl polymer, a peptide-like backbone to which nucleobases are attached via acyl linkers. These DNA-mimicking molecules, consisting of the moieties of substituted N-[2-aminoethyl]glycine, were developed by Nielsen and Burchardt [5] and subsequently used in routine solid phase synthesis based on the Fmoc strategy [7,8]. Peptide nucleic acid (PNA) was developed as a ligand for the recognition of double-stranded DNA. Since then, PNA has emerged as a powerful new biomolecular tool with a wide range of important applications [9,10,11,12,13]. The unique chemical, physical, and biological properties of PNA have been exploited to produce biomolecular tools, antisense molecules, antigens, molecular probes and biosensors. In recent years, PNA has received attention in the field of template-guided synthesis [11]. In this article, Sayers et al. presented PNA-based template-guided selenocystine–selenoester ligation, which was then applied to the rapid detection of oligonucleotides.

Photochemical reactions are a very useful and convenient tool to modulate organic compounds by irradiation with a specific wavelength [14,15,16]. Ji et al. described the visible light-induced metathesis of diselenides in 2014 [17]. Since then, there have been many publications on light-driven metathesis based on disulfides, diselenides, and ditellurides [18,19]. However, these studies were based on low-molecular-weight compounds. Recently, we presented for the first time a photoinduced diselenide-based metathesis in peptides [20]. The reaction takes place under visible light under mild conditions (room temperature) and allows for the rapid exchange of peptide chains within the diselenide bridge formed by selenocysteine residues. However, it should be noted that these reactions proceed with an efficiency in the range of 40–50%. In this paper, we present the concept of studying the photochemical modulation of diselenide bonds by visible light using diselenide-modified PNA conjugates. This study takes into account the template effect resulting from efficient hybridization between complementary PNA strands. Accordingly, we investigated whether the proper nucleobase recognition of complementary PNA conjugates containing N-(selenoethyl)glycine or selenocysteine residues affects the efficiency of visible light-induced metathesis based on the template effect. We believe that this concept may provide a convenient method for rapid nucleic acid detection in the future.

## 2. Materials and Methods

### 2.1. Reagents

Solvents for peptide synthesis (analytical grade): dimethylformamide-DMF; trifluoroacetic acid-TFA were obtained from Sigma-Aldrich (St. Louis, MO, USA) and J. T. Baker (diethyl ether). The H-Rink amide Chemmatrix^®^ resin was purchased from Sigma-Aldrich (St. Louis, MO, USA). The (benzotriazole-1-yloxy)tripyrrolidinophosphonium hexafluorophosphate (PyBOP) coupling reagent was obtained from Novabiochem^®^ (London, United Kingdom). Four Fmoc–PNA derivatives were purchased from ASM Research Chemicals GmbH (Hannover, Germany). All standard amino acids derivatives were purchased from Peptydy. Pl (Zblewo, Poland). Bromoacetic acid and 2-bromoethylamine hydrobromide were obtained from Sigma-Aldrich (St. Louis, MO, USA). Fmoc–OSu reagent was purchased from Novabiochem (London, UK). Glyoxalic acid monohydrate was purchased from Merck (Darmstadt, Germany). Solvents for LC-MS analyses and HPLC separations, acetonitrile (MeCN), methanol (MeOH), and formic acid (HCOOH), were purchased from Baker (Sanford, ME, USA).

### 2.2. PNA Conjugates and PNA Templates Synthesis

All presented PNA conjugates were prepared on a solid support according to the standard Fmoc protocol. All solid-supported PNA conjugations were performed in a plastic syringe with polypropylene frit. A commercially available H-Rink Amide ChemMatrix^®^ resin was used. The individual sequences were selected so that the resulting product pairs were complementary to each other and arranged in a parallel or antiparallel fashion. In the first step, bromoacetic acid was bound to the solid support using a 3-fold excess of the substrate and DIC as coupling agent. Next, the previously prepared 2-(4-methoxybenzylseleno)ethylamine was applied in a 3-fold excess and stirred overnight. This selenium-based moiety was separated from the PNA sequence by a linker consisting of one, two, or three β-alanine residues, depending on the sequence. Alternatively, the previously synthesized N-Fmoc-(Se-Mob-selenoethyl)glycine moiety was used instead of bromoacetic acid and selenosteamine attachment. Coupling of the respective PNA derivatives was performed using a PyBOP in DMF. Both the Fmoc–PNA or amino acid derivatives and the coupling agent were used in 3-fold excess. However, in the case of the first β-alanine residue coupled to the secondary amine formed, a 6-fold excess of the required reagents was used. In addition, all coupling reactions and Fmoc deprotection (25% piperidine/DMF) were assisted by sonication for 15 min and 3 min, respectively. The iterative coupling reactions were monitored by the ninhydrin test. During the assembly of the anti-parallel sequence of the PNA conjugate, one of the sequences contained a selenocysteamine or selenocysteine moiety attached to the N-terminus. Similarly, a linker consisting of one, two, or three β-alanine residues was attached to the PNA sequence according to the same Fmoc protocol. Bromoacetic acid (3 eq.) was then coupled (3 eq. DIC as coupling agent), followed by a reaction with 2-(4-methoxybenzylseleno)ethylamine, which also proceeded overnight. DIPEA (2 eq.) was added to improve the reaction yield. The crude product was cleaved from the resin using a mixture of TFA/H_2_O/TIS (95:2.5:2.5, *v/v/v*) for 2 h at room temperature. The standard cleavage procedure allows for the obtainment of the peptide without protection of the group, except for Mob protection. Therefore, an additional treatment with a mixture consisting of 5%DMSO/TFA for 5 h was necessary. This procedure resulted in the formation of an oxidized product. The peptides obtained were precipitated in cold diethyl ether and then lyophilized.

### 2.3. Purification and Characterization of PNA Conjugates

All the products obtained on solid support were purified after cleavage by preparative reversed-phase HPLC on a Vydac C18 column (22 mm × 9 250 mm), using the following solvent systems: S1 0.1% aqueous TFA, S2 80% acetonitrile + 0.1% TFA, linear gradient from 5 to 70% of B for 50 min, flow rate 7.0 mL/min, UV detection at 254 nm. The resulting fractions were collected and subjected to a lyophilization process. The identities of the products were confirmed by MS analysis using the above-described Shimadzu IT-TOF mass spectrometer equipped with an electrospray (ESI) ionization source. The purity of peptides was analyzed using a Nexera XR (Shimadzu) HPLC system with a UV detection (PDA—254 nm) and an Aeris Peptide XB-C18 column (100 mm × 2.1 mm) with a 3.6 μm bead diameter. The separation conditions were described in the LC-MS section.

### 2.4. Irradiation Experiment

The mixture of PNA conjugates (equimolar) was dissolved in 0.5 mL of water (deionized by the reverse osmosis system; Hydrolab, Poland) and placed in an HPLC vial. Then, the sample was irradiated using an LED lamp (5 W, 250 lm) for 10–90 min. At specified intervals, a volume of 50 µL was taken and analyzed after dilution by LC–UV–MS.

### 2.5. LC–UV–MS

The LC–UV–MS analyses of obtained peptides were carried out on Shimadzu IT-TOF, which is a hybrid system consisting of an ion trap and time of flight mass analyzer. This instrument is also equipped with an electrospray (ESI) ion source. The potential between the spray needle and the orifice was set to 4.5 kV. The spectra were acquired in the *m/z* range of 200–2000. For fragmentation, the collision-induced dissociation—CID—technique was used with argon as the collision gas. The MS2 spectra were recorded using the following parameters: an accurate *m/z* value, 200–3000 *m/z* mass range, 3 Da isolation window was set, ion accumulation of 20 ms, and the collision energy was optimized individually for each ion between 20 and 30 eV. The LC system (Nexera X2 UPLC system) was operated with a mobile phase, consisting of solvent A: 0.1% formic acid in H_2_O and solvent B: 0.1% formic acid in MeCN. The following separation conditions were used: The gradient conditions (B %) were from 5 to 50% B within 15 min—Gradient 1. For more hydrophilic mixtures the following separation conditions were applied: 0 to 30% B within 15 min—Gradient 2. The flow rate was 0.3 mL/min and the injection volume was 2 μL. The separation was performed on an Aeris Peptide XB-C18 column (1000 mm × 2.1 mm) with a 3.6 μm bead diameter. The samples of peptides were dissolved in 400 μL of water:acetonitrile mixture (95:5). UV control using a PDA detector (190–380 nm) was carried out in concert with MS analysis.

## 3. Results and Discussion

### 3.1. The Synthesis of PNA Conjugates Containing Diselenide Bridge

**Figure 1:**
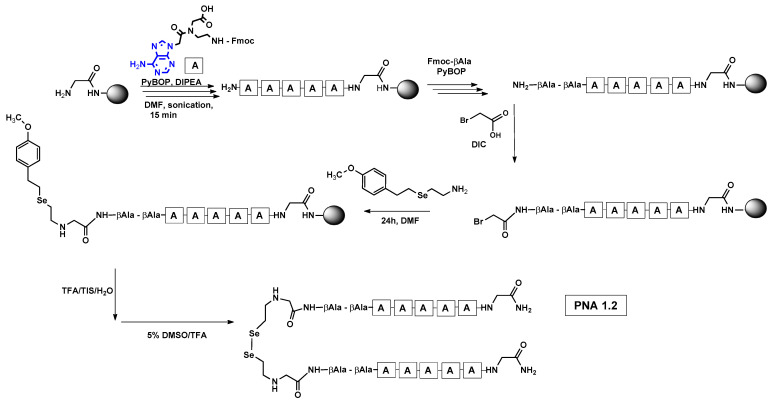
Schematic representation of solid-phase assembly of PNA 1.2 conjugates containing diselenide bond.

We are interested in developing a technique for the visible light-driven metathesis of PNA conjugates containing diselenide bonds that is additionally supported by a template effect. To test our hypothesis, we first designed a model reaction system consisting of a penta-nucleobase PNA (TTTTT) (all the structures in Table 1) bearing an N-terminal oxidized selenocysteamine moiety (**1.1**) and a complementary penta-nucleobase PNA (AAAAA) sequence containing the same selenium-based modification (**1.2**). As a result, a “hairpin” molecular architecture was achieved. The assembly of the PNA conjugates was based on solid-phase synthesis (SPS) using the readily available H-Rink Amide ChemMatrix^®^ resin and generally involved the routine iterative coupling of the respective Fmoc–PNA derivatives (Figure 1).

**Figure 2:**
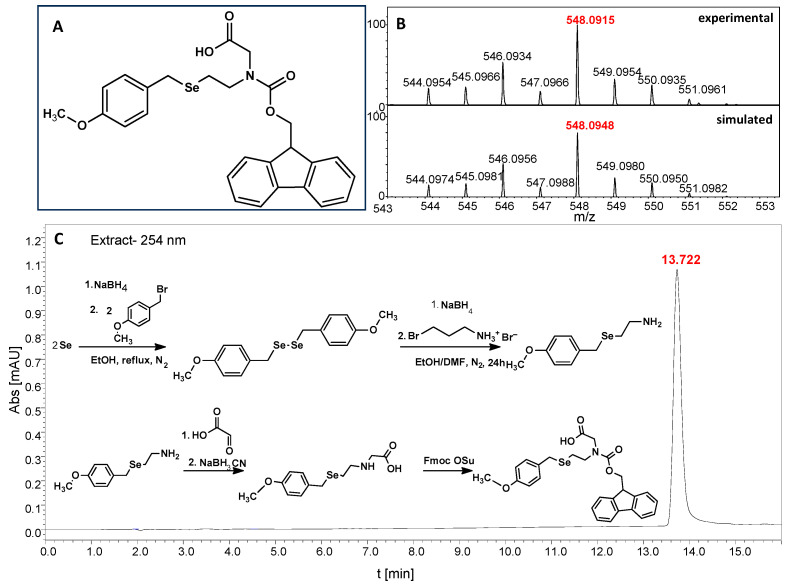
(**A**)The structure of N-Fmoc-(Se-Mob-2-selenoethyl)glycine. (**B**) MS spectrum of the Fmoc-based building block. (**C**) HPLC chromatogram obtained for Fmoc-based building block and the synthetic pathway for Fmoc derivative.

In particular, this is the first time that we have successfully applied the sonication-assisted solid-phase synthesis of PNA, which has recently been developed by our research group with peptide synthesis [21]. Although the synthetic procedure was performed manually, the use of sonication significantly reduced the coupling and Fmoc deprotection times to 15 and 3 min, respectively, as opposed to the usual 120 and 20 min. In addition, we did not start the synthesis with the addition of the first Fmoc–PNA derivative, but with the attachment of glycine, in order to protect against unwanted cyclization during the coupling of the second PNA residue, which is particularly prone to cyclization, which prevents further elongation [22]. A “hairpin” molecular architecture was also composed of a β-alanine residue linking the PNA sequence to a diselenide-based fragment [23,24]. The latter was introduced at the N-terminus as 2-(4-methoxybenzylseleno)ethylamine, which reacted with bromoacetic acid previously attached to β-alanine. Alternatively, we achieved the efficient synthesis of the N-Fmoc-(*Se*-Mob-2-selenoethyl)glycine building block, which can be conveniently used in the standard Fmoc-based solid-supported strategy. The synthetic pathway (Figure 2) involved the preparation of selenocysteamine with methoxybenzyl (Mob) protection. This compound was then subjected to reductive amination with glyoxylic acid followed by the incorporation of an N-Fmoc protecting group (see Figures S1–S10, Supplementary Materials). Such an approach requires additional solution phase synthetic activity, but the subsequent assembly of the diselenide-based PNA conjugates conveniently follows a standard Fmoc protocol. Nevertheless, both routes presented, involving the direct attachment of selenocysteamine to the solid phase or the use of a Fmoc derivative, lead to the desired products. However, after many syntheses, we would like to emphasize that the synthetic route using the building block leads to slightly better yields and purity of the compounds obtained. The resin-bound PNA conjugates were then subjected to acid cleavage from the solid support using a standard cocktail of TFA/TIS/H_2_O (95;2.5;2.5; *v:v:v*). The final step in the preparation of the model compounds was to remove the 4-methoxybenzyl protection in solution by treatment with 5% DMSO/TFA for 5 h, forming a diselenide homodimer. After global acid cleavage and purification by preparative reverse phase HPLC, the PNA conjugate **1.1** was obtained. The complementary PNA sequence was prepared using the same PNA oligomerization methodology, finally yielding PNA conjugate **1.2**. Due to the redox potential of selenocysteamine, it exists predominantly as a diselenide dimer and can therefore be stored intact for a longer period of time. All synthetic protocols and analytical data (See Figures S11–S16) are described in Section 2.2 and Section 2.3 and the Supplementary Materials.

### 3.2. Metathesis Reaction in Two-Component PNA Conjugates System

With the complementary diselenide-based PNA **1.1** and PNA conjugates **1.2** in hand, we were then able to carry out a proof-of-concept study of a templated and light-driven metathesis reaction within the diselenide bond. The experiment was carried out by simply mixing an equimolar amount (final concentration 150 µM) of complementary PNA conjugates and dissolving them in deionized water. In our previous work on metathesis in peptides, the reaction was performed in methanol. However, this solvent is poor at dissolving PNA, so water was the solvent of choice. The resulting solution was transferred to a glass vial and then irradiated with an LED lamp. After 15 min, a small portion of the aqueous mixture was taken and analyzed by LC-MS after dilution. Due to the very similar physico-chemical properties of the components of the analyzed mixture and the observed peak broadening, the initial separation results were highly unsatisfactory. Optimization experiments were therefore required, which subsequently revealed the need for column thermostatting at 50 °C. A thorough analysis of the results obtained clearly demonstrated the visible light-driven conversion of diselenide homodimers, but the level of yield obtained was not significantly different from that previously observed for the peptides—in the range of 40–50% (See Figures S17 and S18 and Supplementary Materials).

**Figure 3:**
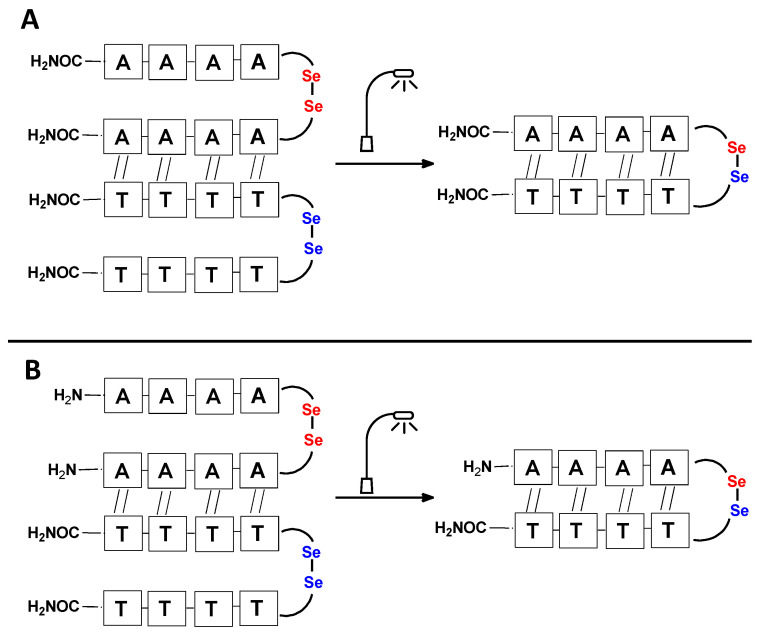
Schematic representation of diselenide-based visible light-induced metathesis in PNA. (**A**). parallel direction of hybridization; (**B**). antiparallel direction of hybridization.

**Figure 4:**
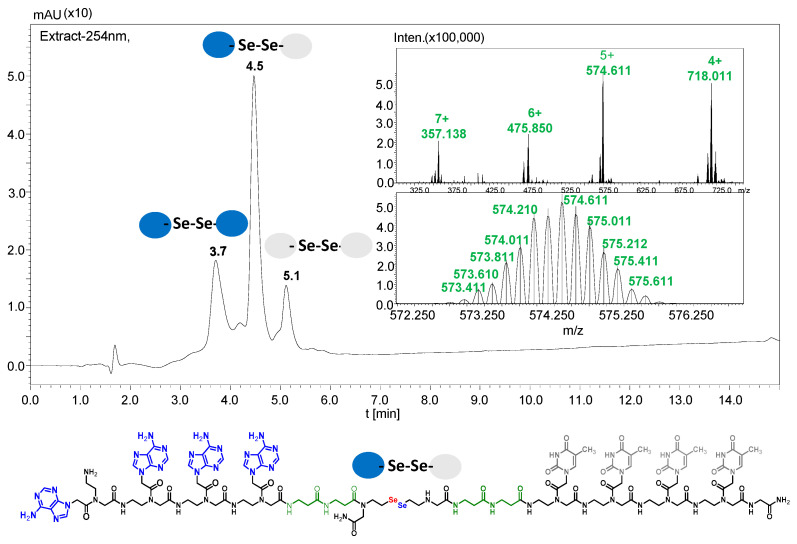
Figure showing the analysis results of PNA 2.1 and 2.2 conjugates after 15 min of irradiation. The structure and MS spectrum of the metathesis product are shown.

It should be noted that the conjugates designed so far hybridize in a parallel direction (Figure 3A). Data from the literature indicate that the interactions of the complementary PNA strands in the anti-parallel direction are clearly preferred, and the strands obtained are characterized by greater stability [25,26]. Following these data, we assembled PNA conjugate **2.1** and **2.2** conjugates according to a similar procedure, as described for the previous model compounds. In addition, we decided to extend the linker by incorporating a β-alanine residue, which we believed would facilitate the turning of the molecular hairpin. This time, we constructed a tetra-nucleobase PNA in which the tetra-adenine version of the PNA **2.1** conjugate carried a C-terminal diselenide moiety. The assembly was analogous and involved the direct attachment of bromoacetic acid to a solid support followed by an overnight reaction with Se-Mob-selenocysteamine. The peptide linker, consisting of two β-alanine residues and the appropriate PNA nucleobases, was then added in sequence. As the linker was coupled to a secondary amine (selenocysteamine residue), a 5-fold excess of Fmoc-protected β-alanine and coupling agent (PyBOP) was required for successful amino acid coupling. TFA-based cleavage, the oxidative removal of Mob protection, and preparative purification yielded the PNA **2.1** and **2.2** conjugates, which were used in the next photochemical experiments after lyophilization (see Figures S19–S24, Supplementary Materials). We repeated the visible light irradiation of an equimolar amount of newly synthesized PNA conjugates under similar conditions. This time, we controlled the conversion rate over time by taking a small portion of the reaction mixture after 15, 30, and 45 min and performing immediate LC–UV–MS analysis (See Figures S25 and S26, Supplementary Materials). The structural changes introduced proved to be very beneficial and resulted in a significant increase in the conversion of a diselenide-based homodimer into a complementary heterodimer. The analysis of the HPLC chromatogram (detection 254 nm) in Figure 4 shows the clearly dominant signal corresponding to the product of the photochemical transformation. The calculated yield, based on the integration of the peak area, was approximately 75%. Surprisingly, the maximum yield of photochemical conversion was reached in only 15 min, and the further prolongation of the visible light irradiation of the sample did not result in any additional conversion to the desired product. We believe that the explanation lies in the equilibrium nature of this reaction, which, due to the self-complementary nature of the PNA reactants, may allow the reaction to proceed in the opposite direction. To demonstrate that the template-driven photochemical transformation presented here is not limited to the polyadenine and polytymine PNA conjugates, we have also synthesized the second pair of PNA homodimers (**3.1** and **3.2**) composed of all four PNA nucleobases (ACTG and CAGT), which are complementary to each other (see Figures S27–S34, Supplementary Materials). These conjugates were assembled similarly but with one exception. Namely, the (2-selenoethyl)glycine residue was not synthesized directly on a solid support, but was incorporated as a building block (N-Fmoc-(*Se*-Mob-2-selenoethyl)glycine) prepared according to the synthesis described in Supplementary Materials. The obtained and purified PNA conjugates **3.1** and **3.2** were then subjected to visible irradiation in an aqueous solution. As expected, the diselenide dimer was formed as a predominant component, based on MS and HPLC data (see Figures S35–S38, Supplementary Materials). At this point, however, it should be noted that due to the very high physicochemical similarity of the PNA sequences, the retention times are very similar to each other. As a result, the calculation of the exact yield is an estimate and is above 70%.

**Figure 5:**
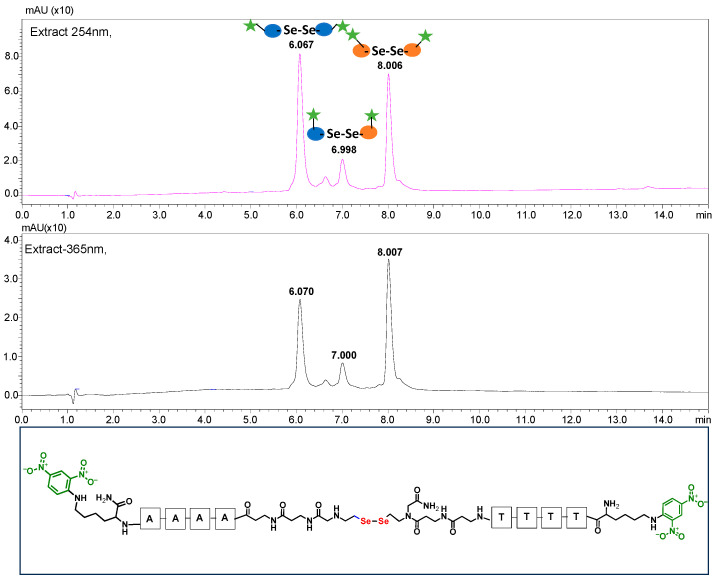
HPLC chromatogram obtained for the PNA conjugates **4.1** and **4.2** mixtures containing 2,4-dinitrophenyl chromophore after 60 min of visible light irradiation (at 254 and 365 nm). The bottom figure shows the structure of the metathesis product.

Other experimental work has also focused on the problem of quantifying photochemical transformations. UV absorption at a specific wavelength is usually considered to be an efficient tool for quantifying the compounds being analyzed. This is generally true, but when comparing different or even similar compounds that differ in UV absorption at the same wavelength, such analyses can be treated as an estimate due to possible quantitative errors. To overcome this drawback, we decided to incorporate a chromophore group with highly specific UV absorption. We therefore selected the 2,4-dinitrophenyl moiety and attempted to incorporate it under solid phase conditions as a direct aromatic nucleophilic substitution between the PNA conjugate and 2,4-dinitrofluorobenzene. While the reaction with the free α-amino group of the PNA nucleobase was successful, direct incorporation into the secondary amino group (selenocysteamine moiety present at the N-terminus of the PNA conjugate) was not achieved. Therefore, we decided to use the Fmoc-Lys(DNP)-OH derivative and then incorporate it into the PNA conjugate sequence (**4.1** and **4.2**) during solid-phase synthesis (see Figures S39–S44, Supplementary Materials). With the ready-made PNA conjugates in hand, we performed further photochemical experiments. Using similar 2,4-dinitrophenyl-containing conjugates, we irradiated the reaction mixture consisting of **4.1** and **4.2** with visible light for 15 min (Figure 5, see Figures S45 and S46, Supplementary Materials) and analyzed it by LC–UV–MS at 365 nm (wavelength characteristic for the chromophore) and 254 nm. Unexpectedly, a very small amount underwent metathesis and even prolonged irradiation did not cause a significant change in the substrate/product ratio. Considering the high yield of photoinduced metathesis of the same PNA conjugate, but without a chromophore group, the only rational explanation for this observation is the absorption of LED light by a chromophore present in the molecule. The 2,4-dinitrophenyl chromophore shows similar absorption with the visible LED light used in the experiments and is likely to have a filtering effect. Summarizing the experimental data obtained, the presence of chromophore groups, especially those showing absorption in visible light, is prohibited for the presented visible light-induced photochemical transformation.

### 3.3. Concentration-Dependent Study of Photoinduced Metathesis

According to the concept of the templated effect, the proximity of the reactive groups increases, leading to an increase in the effective molarity. This in turn implies the possibility of a reaction taking place at a lower concentration level, in contrast to untemplated reactions. Therefore, as part of the research completed in this project, we carried out a series of photochemical experiments using a series of dilutions of PNA **2.1** and PNA **2.2** combined in equimolar amounts. A stock solution of 130 µM was prepared and kept in the dark. The following concentrations of PNA conjugates were tested: 130 µM, 65 µM, 13 µM, 6.5 µM, and 2.5 µM. As predicted, very similar results were obtained for each dilution tested. When comparing the chromatograms corresponding to the 130 and 2.5 µM concentrations, no significant differences can be seen since the reactants **2.1** and **2.2** were consumed to almost the same extent (see Figures S25 and S26, Supplementary Materials). The possibility of visible light-induced metathesis at the nanomolar level is not excluded and was confirmed by mass spectrometry as a more sensitive analytical tool, whereas the detection of the mixture components at such a low concentration level was limited in this case by the poor response of the UV detector at 254 nm.

Nevertheless, these dilution experiments suggest that the visible light-driven photochemical metathesis within the diselenide bond, supported by the template presented here, is likely to proceed in a concentration-independent manner. A very similar effect has been observed by Sayers et al. [11], who reported the selenium-based native chemical ligation (NCL) of two complementary PNA strands and the possibility of reacting at a much lower concentration level driven by the template effect. The template effect can only occur when there is hybridization between complementary nucleobase pairs. To demonstrate the formation of specific interactions, the melting temperature (Tm) study was performed for the irradiation mixture. A plot of the temperature dependence of the absorbance measured at 260 nm is shown in Figure S84. This graph shows a characteristic sigmoidal curve, which, in this case, is relatively broad, which can be explained by the intramolecular nature of the hybridization. The determined melting temperature of this system is about 43 °C, which is as expected, given the presence of only four nucleobases. The UV-Vis-based procedure used for the T_m_ study has been described in Supplementary Materials.

### 3.4. Metathesis Reaction of PNA Conjugates in a Three-Component System

Encouraged by the satisfactory results obtained for the template-assisted photoinduced metathesis of the PNA conjugates, in which the target molecule contained complementary sequences, we also decided to study the three-component system consisting of a model PNA template and two PNA conjugates with diselenide bonds.

**Figure 6:**
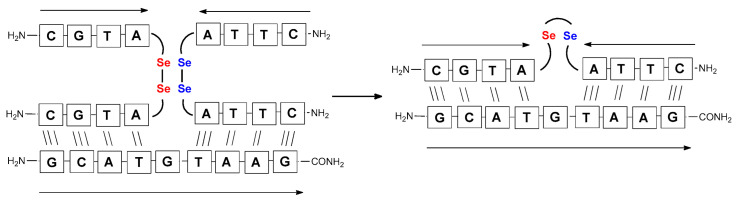
Schematic representation of diselenide-based photoinduced metathesis reaction of PNA conjugates in a three-component system.

Unlike the previous experiments, the latter two were not complementary to each other, but to the PNA template. In other words, we were interested in testing whether the PNA conjugates would be able to recognize the corresponding nucleobases in the template, which in turn would facilitate the photoinduced metathesis reaction in diselenide-based PNA homodimers, since they are not complementary to each other. The schematic representation is shown in Figure 6. To test our hypothesis, we assembled a model PNA sequence composed of 9-mer GCATGTAAG nucleobases (PNA template **T1**) using a standard Fmoc-based solid-phase synthesis protocol, as described for the previous compounds (see Figures S47–S49, Supplementary Materials). A C-terminal lysine residue was included in the sequence to prevent unwanted solid-phase cyclization during the coupling step of the second Fmoc derivative and to improve the solubility of the oligomer. Next, we synthesized the tetra-nucleobase PNA conjugate **5.1** (ACGT) with a longer linker consisting of one β-Ala residue and the (2-selenoethyl)glycine moiety at the N-terminus. Similarly, a second diselenide-based homodimer **5.2** (TGCA) was assembled with an analogous linker and a selenocysteamine residue at the C-terminus (the structures and analytical data for all the PNA conjugates presented in this section are provided in Supplementary Materials, see Figures S59–S80). This type of architecture should allow the homodimer strands to hybridize in the antiparallel direction. However, it should be noted that the conjugates obtained were complementary to eight PNA nucleobases, leaving the guanine at position 5 unpaired. This procedure was intentional and was intended to facilitate the formation of the appropriate “hairpin” architecture as a result of light-driven metathesis. After the preparative purification and LCMS analysis, we proceeded with the visible light irradiation experiments. As before, equal amounts of diselenide-based PNA homodimers and 1.2 eq. of PNA template were weighed, mixed in a glass vial, and dissolved in 500 µL of water. Unfortunately, the HPLC analysis of a sample taken after 30 min did not show the presence of any metathesis product. The prolonged irradiation did not change the composition of the mixture. Following this observation, we decided to change the length of the linker by one β-Ala residue (PNA conjugate **6.1** and **6.2**) and two additional β-Ala in the following variant (PNA conjugate **7.1** and **7.2**). In addition, we prepared other variants of the template that differed (see Figures S50–S54, ESI) in the removal of the guanine at position 5 (**T2**) (all nucleobases complementary to the PNA conjugates) and in the presence of an additional guanine inserted after the guanine at position 5 in the next sequence (**T3**) (two nucleobases remained unpaired) (see Figures S55–S58, Supplementary Materials). In short, we obtained a total of three pairs of diselenide-based PNA conjugates, differing in the length of the linker between the PNA sequence and the selenocysteamine moiety, and three sequences of PNA templates containing one, two, or three unpaired guanine derivatives. The modifications made aimed to obtain more variants of the molecular constructions, allowing for the tuning of the visible light-driven metathesis. Additionally, we carried out a study on the melting temperature (Tm) of the mixture consisting of the PNA template (**T1**) and the complementary PNA conjugates (**6.1** and **6.2**). This UV-Vis-based experiment was designed to demonstrate the hybridization between template and complementary conjugates. The plot with a characteristic sigmoidal curve is provided in the Supplementary Materials (see Figure S85). In contrary to the self-complementary PNA system, the determined T_m_ (32 °C) is lower; however, the characteristic inflection on the sigmoidal curve was sharper. Nevertheless, despite the fact that the T_m_ was relatively low, the study showed the formation of interactions between complementary nucleobases. The next step was to study the prepared oligomers in different combinations in photochemical experiments. We carefully analyzed the HPLC and MS data obtained; only in the system consisting of PNA conjugate **6.1**, **6.2**, and **T1** the product was formed (see Figures S81 and S82, Supplementary Materials). Thus, among the structures obtained, these proved to be the most suitable ones. However, according to the MS and HPLC data, the metathesis yield was significantly lower than expected and only a few percent of the product was formed. On the other hand, when this experiment was performed without the template (**T1**), no metathesis product was observed at all. Thus, the importance of the template in facilitating the reaction is evident. However, this observation will be the subject of further research in the future.

## 4. Conclusions

In conclusion, we have carried out the efficient synthesis of several PNA conjugates on a solid support containing a (2-selenoethyl)glycine moiety at both the C- and N-terminus of the sequence. Two synthetic routes were demonstrated, involving either the direct attachment of Se-Mob selenocysteamine to bromoacetic acid in the solid phase, or the prior preparation of N-Fmoc-(Se-Mob-2-selenoethyl)glycine as a building block and its subsequent application to solid-phase synthesis using a routine Fmoc strategy. TFA-based cleavage followed by the removal of Mob protection generated diselenide homodimers. The resulting PNA conjugates were successfully subjected to a metathesis reaction under visible light irradiation within 15 min. Due to the template effect, which is explained by the specific recognition of complementary nucleobases, the homodimer–heterodimer conversion proceeded with a much higher yield compared to its conventional peptide counterpart. However, we would like to emphasize that only self-complementary (two-component system) PNA conjugates worked very efficiently in our study. In the case of PNA conjugate homodimers that are not complementary to each other, but are complementary to the PNA template (ternary system), the metathesis reaction proceeds only with low yield. In addition, we demonstrated the feasibility of the reaction over a wide range of substrate concentrations, and even 2 µM proved to be as effective as over 100 µM. Based on the data obtained, we have shown that the PNA conjugate must not contain additional chromophore groups such as dabcyl or 2,4-nitrofluorophenyl. The reason for this is the effective absorption of visible light, resulting in too little energy being available for homolytic diselenide bond cleavage.

## Figures and Tables

**Table 1:** Schematic representation of synthesized PNA conjugates and PNA templates.

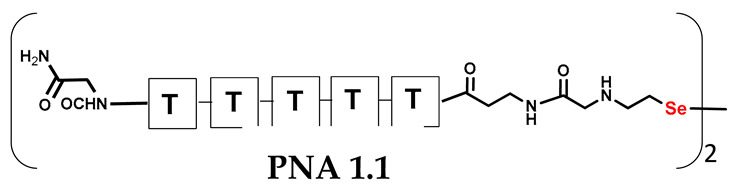	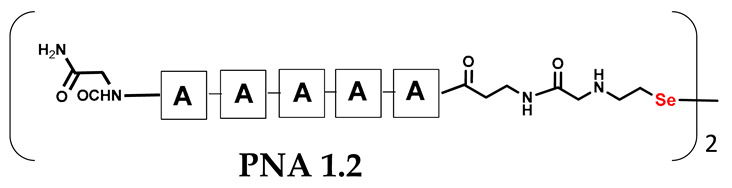
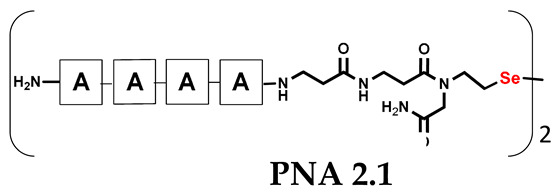	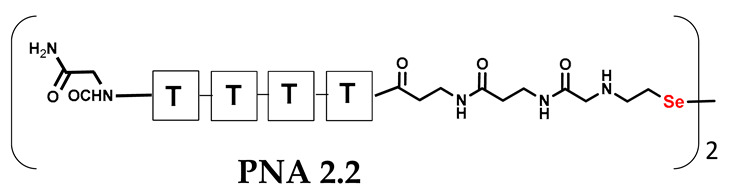
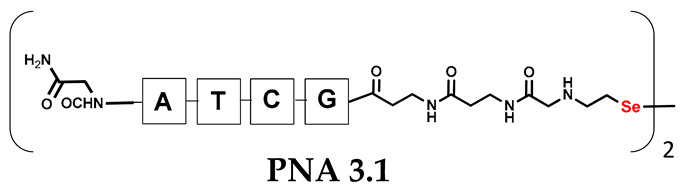	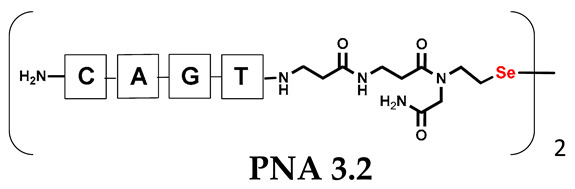
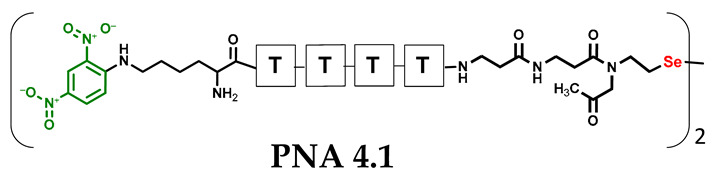	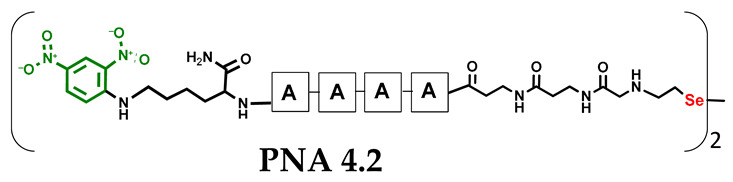
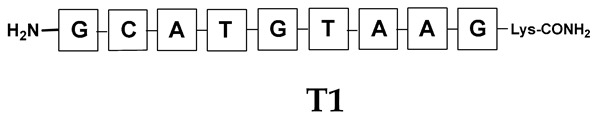	-
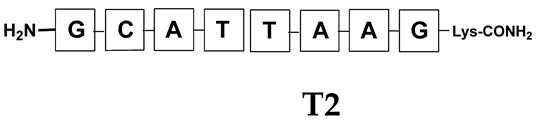	-
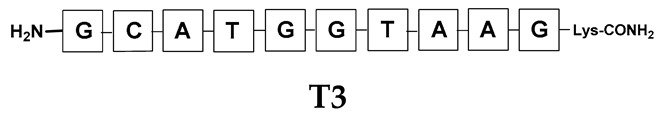	-
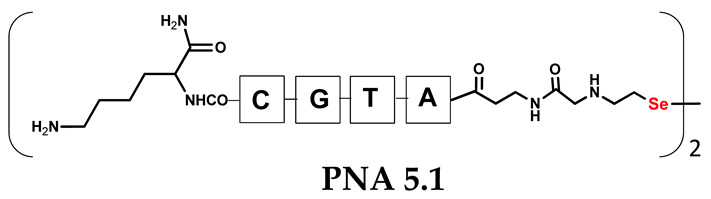	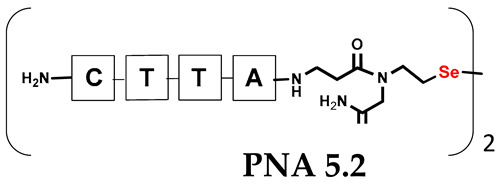
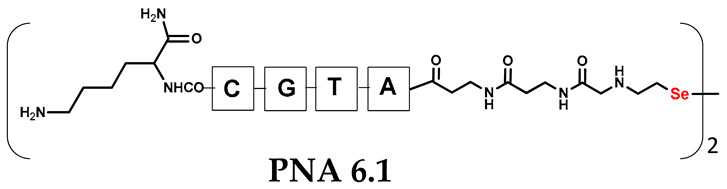	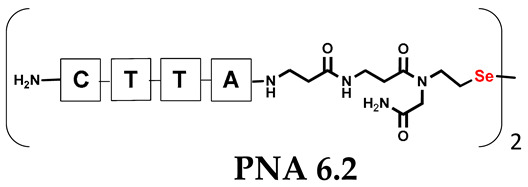
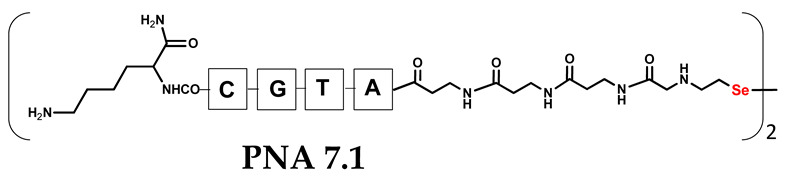	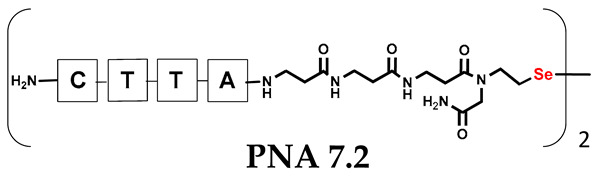

## Data Availability

Data are contained within the article and Supplementary Materials.

## Supplementary Materials

The following supporting information can be downloaded at https://www.mdpi.com/article/10.3390/biom13111676/s1, Figures S1–S85. Figure S1 1H NMR (CD3OD, 500 MHz, 300 K) spectrum of 2-(4-methoxybenzylseleno)ethylamine; Figure S2 13C NMR (CD3OD, 126 MHz, 300 K) spectrum of 2-(4-methoxybenzylseleno)ethylamine; Figure S3 ^77^Se NMR (CD_3_OD, 114 MHz, 300 K) spectrum of 2-(4-methoxybenzylseleno)ethylamine9; Figure S4. ESI-MS spectrum of N- (Se-Mob-2-selenoethyl)glycine obtained after reductive amination;Figure S5. ESI-MS spectrum obtained for purified N-Fmoc-(Se-Mob-2-selenoethyl)glycine; Figure S6. ESI-MS/MS spectrum obtained for purified N-Fmoc-(Se-Mob-2-selenoethyl)glycine; Figure S7 ^1^H NMR (CDCl_3_) 500 MHz, 300 K) spectrum obtained for purified N-Fmoc-(Se-Mob-2-selenoethyl)glycine; Figure S8 ^13^C NMR (CDCl_3_, 126 MHz, 300 K) spectrum obtained for purified N-Fmoc-(Se-Mob-2-selenoethyl)glycine; Figure S9 2D HSQC NMR spectrum obtained for purified N-Fmoc-(Se-Mob-2-selenoethyl)glycine; Figure S10 2D COSY NMR spectrum obtained for purified N-Fmoc-(Se-Mob-2-selenoethyl)glycine; Figure S11 Structure of PNA conjugate 1.1 Figure S12 ESI-MS spectrum obtained for PNA 1.1; Figure S13 HPLC chromatogram obtained for PNA 1.1; Figure S14 Structure of PNA conjugate 1.2; Figure S15 ESI-MS spectrum obtained for PNA 1.2; Figure S16 HPLC chromatogram obtained for PNA 1.2; Figure S17 LC-MS spectrum (TIC and XIC) obtained after irradiation of PNA conjugates 1.1 and 1.2 after 15 min of irradiation; Figure S18 ESI-MS spectrum obtained for the product after irradiation of PNA conjugates 1.1 and 1.2; Figure S19. Structure of PNA conjugate 2.1; Figure S20 LC-MS chromatogram and spectrum obtained for PNA conjugate 2.1; Figure S21 HPLC chromatogram obtained for PNA conjugate 2.1; Figure S22 Structure of PNA conjugate 2.2; Figure S23 ESI-MS spectrum obtained for PNA conjugate 2.2; Figure S24 HPLC chromatogram obtained for PNA conjugate 2.2; Figure S25 HPLC chromatogram of the mixture obtained after visible-light irradiation of PNA conjugate 2.1 and 2.2 with concentrations of substrates of 130 µM; Figure S26 HPLC chromatogram of the mixture obtained after visible-light irradiation of PNA conjugate 2.1 and 2.2 with concentrations of substrates of 2.5 µM; Figure S27 Structure of PNA conjugate 3.1; Figure S28 ESI-MS spectrum obtained for PNA conjugate 3.1; Figure S29 ESI-MS/MS (CE 20 eV) spectrum obtained for PNA conjugate 3.1; Figure S30 HPLC chromatogram obtained for PNA conjugate 3.1; Figure S31 Structure of PNA conjugate 3.2; Figure S32 ESI-MS spectrum obtained for PNA conjugate 3.2; Figure S33 ESI-MS/MS (CE 20 eV) spectrum obtained for PNA conjugate 3.2; Figure S34 HPLC chromatogram obtained for PNA conjugate 3.2; Figure S35 Structure of metathesis product obtained after visible-light irradiation of PNA conjugate 3.1 and 3.2; Figure S36 HPLC chromatogram obtained for the mixture of PNA conjugate 3.1 and 3.2 before irradiation; Figure S37 Chromatogram HPLC obtained after visible-light irradiation of PNA conjugate 3.1 and 3.2.; Figure S38 ESI-MS spectrum obtained for the mixture after visible-light irradiation of PNA conjugate 3.1 and 3.2 (the most abundant peaks correspond to target compound); Figure S39 Structure of PNA conjugate 4.1 containing 2,4-dinitrofluorophenyl chromophore; Figure S40 HPLC chromatogram obtained for PNA conjugate 4.1; Figure S41 ESI-MS spectrum obtained for PNA conjugate 4.1; Figure S42 Structure of PNA conjugate 4.2 containing 2,4-dinitrofluorophenyl chromophore; Figure S43 ESI-MS spectrum obtained for PNA conjugate 4.2; Figure S44 HPLC chromatogram obtained for PNA conjugate 4.2; Figure S45 Structure of the target product after visible-light irradiation of PNA conjugate 4.1 and 4.2; Figure S46 ESI-MS spectrum obtained for target the product after visible-light irradiation of PNA conjugate 4.1 and 4.2; Figure S47 Structure of PNA template T1; Figure S48 HPLC chromatogram obtained for PNA template T1; Figure S49 ESI-MS spectrum obtained for PNA template T1; Figure S50 ESI-MS/MS (CE 25 eV) spectrum obtained for PNA template T1; Figure S51 Structure of PNA template T2; Figure S52 ESI-MS spectrum obtained for PNA template T2; Figure S53 ESI-MS/MS (CE 25 eV) spectrum obtained for PNA template T2; Figure S54 HPLC chromatogram obtained for PNA template T2; Figure S55 Structure of PNA template T3; Figure S56 ESI-MS spectrum obtained for PNA template T3; Figure S57 ESI-MS/MS (CE 25 eV) spectrum obtained for PNA template T3; Figure S58 HPLC chromatogram obtained for PNA template T3; Figure S59 Structure of PNA conjugate 5.1; Figure S60 HPLC chromatogram obtained for PNA conjugate 5.1; Figure S61 ESI-MS spectrum obtained for PNA conjugate 5.1; Figure S62 ESI-MS/MS (CE 22eV) spectrum obtained for PNA conjugate 5.1; Figure S64 ESI-MS spectrum obtained for PNA conjugate 6.1; Figure S65 ESI-MS/MS (CE 25 eV) spectrum obtained for PNA conjugate 6.1; Figure S66 HPLC chromatogram obtained for PNA conjugate 6.1; Figure S67 Structure of PNA conjugate 7.1; Figure S68 HPLC chromatogram spectrum obtained for PNA conjugate 7.1; Figure S69 ESI-MS spectrum obtained for PNA conjugate 7.1; Figure S70 Structure of PNA conjugate 5.2; Figure S71 HPLC chromatogram obtained for PNA conjugate 5.2; Figure S72 ESI-MS spectrum obtained for PNA conjugate 5.2; Figure S73 ESI-MS/MS (CE 25 eV) spectrum obtained for PNA conjugate 5.2; Figure S74 Structure of PNA conjugate 6.2; Figure S75 HPLC chromatogram obtained for PNA conjugate 6.2; Figure S76 ESI-MS spectrum obtained for PNA conjugate 6.2; Figure S77 ESI-MS/MS (CE 25 eV) spectrum obtained for PNA conjugate 6.2; Figure S78 Structure of PNA conjugate 7.2; Figure S79 HPLC chromatogram obtained for PNA conjugate 7.2; Figure S80 ESI-MS spectrum obtained for PNA conjugate 7.2; Figure S81 HPLC chromatograms obtained for 3-component mixtures consisting of PNA conjugate 6.1, 6.2 and PNA template T1, T2 and T3, respectively, after visible-light irradiation for 60 min; Figure S82 MS spectrum (expanded area) obtained for 3-component mixtures consisting of PNA conjugate 6.1, 6.2 and PNA template, T2 after visible-light irradiation for 60 min. Expanded isotopic pattern for the signal corresponding to the metathesis product and its comparison with the simulated one (charge 4+). Figure S83. HPLC chromatogram obtained after irradiation of non-complemenatry PNA conjugates. Figure S84 Melting curve obtained for metathesis product after irradiation of PNA 3.1 and 3.2 Figure S85 Melting curve obtained for 3-component system consisting of template 2, PNA 6.1 and 6.2
