# Supplementary material for: Visible Light-Induced Templated Metathesis of Peptide–Nucleic Acid Conjugates with a Diselenide Bridge
Source: Biomolecules. 2023 Nov 20;13(11):1676. doi: 10.3390/biom13111676 (PMC10669671; doi:10.3390/biom13111676)
Supplement: Supplementary file 1 [file biomolecules-13-01676-s001.zip › biomolecules-2713183-supplementary.pdf]

# VISIBLE LIGHT-INDUCED TEMPLATED METATHESIS OF PEPTIDE NUCLEIC ACID CONJUGATES WITH A DISELENIDE BRIDGE

Mateusz Waliczek*, Wiktoria Gancarz, Paulina Pochwała, Özge Pehlivan, Piotr Stefanowicz*

*Faculty of Chemistry, University of Wrocław, Joliot-Curie 14, 50-383 Wrocław ,Poland.*

*E-mail: mateusz.waliczek2@uwr.edu.pl; piotr.stefanowicz@uwr.edu.pl*

## LIST OF FIGURES

Fig. S 1 $^{1}$H NMR ($CD_3OD$, 500 MHz, 300 K) spectrum of 2-(4-methoxybenzylseleno)ethylamine....... 7
Fig. S 2 $^{13}$C NMR (CD3OD, 126 MHz, 300 K) spectrum of 2-(4-methoxybenzylseleno)ethylamine .. 8
Fig. S 3 $^{77}$Se NMR (CD3OD, 114 MHz, 300 K) spectrum of 2-(4-methoxybenzylseleno)ethylamine ....... 8
Fig. S 4. ESI-MS spectrum of N- (Se-Mob-2-selenoethyl)glycine obtained after reductive amination . 9
Fig. S 5. ESI-MS spectrum obtained for purified N-Fmoc-(Se-Mob-2-selenoethyl)glycine .................. 9
Fig. S 6. ESI-MS/MS spectrum obtained for purified N-Fmoc-(Se-Mob-2-selenoethyl)glycine ......... 10
Fig. S 7 $^{1}$H NMR ($CDCl_3$) 500 MHz, 300 K) spectrum obtained for purified N-Fmoc-(Se-Mob-2-selenoethyl)glycine.......... 10
Fig. S 8 $^{13}$C NMR ($CDCl_3$, 126 MHz, 300 K) spectrum obtained for purified N-Fmoc-(Se-Mob-2-selenoethyl)glycine.......... 11
Fig. S 9 2D HSQC NMR spectrum obtained for purified N-Fmoc-(Se-Mob-2-selenoethyl)glycine... 12
Fig. S 10 2D COSY NMR spectrum obtained for purified N-Fmoc-(Se-Mob-2-selenoethyl)glycine.. 13
Fig. S 11 Structure of PNA conjugate 1.1 .......... 13
Fig. S 12 ESI-MS spectrum obtained for PNA 1.1 .......... 14
Fig. S 13 HPLC chromatogram obtained for PNA 1.1.......... 14
Fig. S 14 Structure of PNA conjugate 1.2 .......... 15
Fig. S 15 ESI-MS spectrum obtained for PNA 1.2 .......... 15
Fig. S 16 HPLC chromatogram obtained for PNA 1.2.......... 16
Fig. S 17 LC-MS spectrum (TIC and XIC) obtained after irradiation of PNA conjugates 1.1 and 1.2 after 15 min of irradiation. .......... 16
Fig. S 18 ESI-MS spectrum obtained for the product after irradiation of PNA conjugates 1.1 and 1.2. .......... 17
Fig. S 19. Structure of PNA conjugate 2.1 .......... 17
Fig. S 20 LC-MS chromatogram and spectrum obtained for PNA conjugate 2.1 .......... 18
Fig. S 21 HPLC chromatogram obtained for PNA conjugate 2.1 .......... 18
Fig. S 22 Structure of PNA conjugate 2.2 .......... 19
Fig. S 23 ESI-MS spectrum obtained for PNA conjugate 2.2 .......... 19
Fig. S 24 HPLC chromatogram obtained for PNA conjugate 2.2 .......... 20
Fig. S 25 HPLC chromatogram of the mixture obtained after visible-light irradiation of PNA conjugate 2.1 and 2.2 with concentrations of substrates of 130 μM .......... 20
Fig. S 26 HPLC chromatogram of the mixture obtained after visible-light irradiation of PNA conjugate 2.1 and 2.2 with concentrations of substrates of 2.5 μM .......... 21
Fig. S 27 Structure of PNA conjugate 3.1 .......... 21
Fig. S 28 ESI-MS spectrum obtained for PNA conjugate 3.1 .......... 22

Fig. S 29 ESI-MS/MS (CE 20 eV) spectrum obtained for PNA conjugate 3.1 .................................... 22
Fig. S 30 HPLC chromatogram obtained for PNA conjugate 3.1 ........................................................ 23
Fig. S 31 Structure of PNA conjugate 3.2 ......................................................................................... 23
Fig. S 32 ESI-MS spectrum obtained for PNA conjugate 3.2 ............................................................. 24
Fig. S 33 ESI-MS/MS (CE 20 eV) spectrum obtained for PNA conjugate 3.2 .................................... 24
Fig. S 34 HPLC chromatogram obtained for PNA conjugate 3.2 ........................................................ 25
Fig. S 35 Structure of metathesis product obtained after visible-light irradiation of PNA conjugate 3.1 and 3.2 ........................................................................................................................................ 25
Fig. S 36 HPLC chromatogram obtained for the mixture of PNA conjugate 3.1 and 3.2 before irradiation. ........................................................................................................................................ 26
Fig. S 37 Chromatogram HPLC obtained after visible-light irradiation of PNA conjugate 3.1 and 3.2. ........................................................................................................................................ 26
Fig. S 38 ESI-MS spectrum obtained for the mixture after visible-light irradiation of PNA conjugate 3.1 and 3.2 (the most abundant peaks correspond to target compound)............................................... 27
Fig. S 39 Structure of PNA conjugate 4.1 containing 2,4-dinitrofluorophenyl chromophore ............. 27
Fig. S 40 HPLC chromatogram obtained for PNA conjugate 4.1 ........................................................ 28
Fig. S 41 ESI-MS spectrum obtained for PNA conjugate 4.1 ............................................................. 28
Fig. S 42 Structure of PNA conjugate 4.2 containing 2,4-dinitrofluorophenyl chromophore ............. 29
Fig. S 43 ESI-MS spectrum obtained for PNA conjugate 4.2 ............................................................. 29
Fig. S 44 HPLC chromatogram obtained for PNA conjugate 4.2 ........................................................ 29
Fig. S 45 Structure of the target product after visible-light irradiation of PNA conjugate 4.1 and 4.2. 30
Fig. S 46 ESI-MS spectrum obtained for target the product after visible-light irradiation of PNA conjugate 4.1 and 4.2 ........................................................................................................................ 30
Fig. S 47 Structure of PNA template T1 ........................................................................................... 31
Fig. S 48 HPLC chromatogram obtained for PNA template T1 .......................................................... 31
Fig. S 49 ESI-MS spectrum obtained for PNA template T1 ................................................................ 31
Fig. S 50 ESI-MS/MS (CE 25 eV) spectrum obtained for PNA template T1 ....................................... 32
Fig. S 51 Structure of PNA template T2 ........................................................................................... 32
Fig. S 52 ESI-MS spectrum obtained for PNA template T2 ................................................................ 32
Fig. S 53 ESI-MS/MS (CE 25 eV) spectrum obtained for PNA template T2 ....................................... 33
Fig. S 54 HPLC chromatogram obtained for PNA template T2 .......................................................... 33
Fig. S 55 Structure of PNA template T3 ........................................................................................... 33
Fig. S 56 ESI-MS spectrum obtained for PNA template T3 ................................................................ 34
Fig. S 57 ESI-MS/MS (CE 25 eV) spectrum obtained for PNA template T3 ....................................... 34
Fig. S 58 HPLC chromatogram obtained for PNA template T3 .......................................................... 35
Fig. S 59 Structure of PNA conjugate 5.1 ......................................................................................... 35
Fig. S 60 HPLC chromatogram obtained for PNA conjugate 5.1 ........................................................ 36
Fig. S 61 ESI-MS spectrum obtained for PNA conjugate 5.1 ............................................................. 36
Fig. S 62 ESI-MS/MS (CE 22eV) spectrum obtained for PNA conjugate 5.1 ...................................... 37
Fig. S 63 Structure of PNA conjugate 6.1 ......................................................................................... 37
Fig. S 64 ESI-MS spectrum obtained for PNA conjugate 6.1 ............................................................. 38
Fig. S 65 ESI-MS/MS (CE 25 eV) spectrum obtained for PNA conjugate 6.1 .................................... 38
Fig. S 66 HPLC chromatogram obtained for PNA conjugate 6.1 ........................................................ 39
Fig. S 67 Structure of PNA conjugate 7.1 ......................................................................................... 39
Fig. S 68 HPLC chromatogram spectrum obtained for PNA conjugate 7.1 ......................................... 40
Fig. S 69 ESI-MS spectrum obtained for PNA conjugate 7.1 ............................................................. 40
Fig. S 70 Structure of PNA conjugate 5.2 ......................................................................................... 41
Fig. S 71 HPLC chromatogram obtained for PNA conjugate 5.2 ........................................................ 41

Fig. S 72 ESI-MS spectrum obtained for PNA conjugate 5.2 .......... 42
Fig. S 73 ESI-MS/MS (CE 25 eV) spectrum obtained for PNA conjugate 5.2 .......... 42
Fig. S 74 Structure of PNA conjugate 6.2 .......... 43
Fig. S 75 HPLC chromatogram obtained for PNA conjugate 6.2 .......... 43
Fig. S 76 ESI-MS spectrum obtained for PNA conjugate 6.2 .......... 44
Fig. S 77 ESI-MS/MS (CE 25 eV) spectrum obtained for PNA conjugate 6.2 .......... 44
Fig. S 78 Structure of PNA conjugate 7.2 .......... 45
Fig. S 79 HPLC chromatogram obtained for PNA conjugate 7.2 .......... 45
Fig. S 80 ESI-MS spectrum obtained for PNA conjugate 7.2 .......... 46
Fig. S 81 HPLC chromatograms obtained for 3-component mixtures consisting of PNA conjugate 6.1, 6.2 and PNA template T1, T2 and T3, respectively, after visible-light irradiation for 60 min .......... 46
Fig. S 82 MS spectrum (expanded area) obtained for 3-component mixtures consisting of PNA conjugate 6.1, 6.2 and PNA template, T2 after visible-light irradiation for 60 min. Expanded isotopic pattern for the signal corresponding to the metathesis product and its comparison with the simulated one (charge 4+) .......... 47
Fig. S 83. HPLC chromatogram obtained after irradiation of non-complemenatry PNA conjugates ... 47
Fig. S 84 Melting curve obtained for metathesis product after irradiation of PNA 3.1 and 3.2 .......... 48
Fig. S 85 Melting curve obtained for 3-component system consisting of template 2, PNA 6.1 and 6.248

## GENERAL INFORMATION

*Mass spectrometry analysis*

Mass spectra were measured at the Mass Spectrometry Laboratory at the Department of Chemistry, University of Wroclaw. A Brucker qTOF Compact instrument was used for this purpose. The apparatus is equipped with an electrospray ion source (ESI) and a hybrid analyzer consisting of a quadrupole combined with a time-of-flight (TOF) analyzer. Its resolution is about 30,000 FWHM. Each measurement was preceded by a calibration process using the quadrature method. Fragmentation experiments were carried out in a CID-type collision chamber, using argon, and the collision energy was optimized in the range of 15-30 eV each time. The analyzed samples were prepared by dissolving about 100 $\mu$g of product in a mixture of ACN/H2O (50 : 50) + 0.1% HCOOH. For the Fmoc derivative, the sample was dissolved in methanol and several microliters of 10mM NaCl were added. The solvents were of "LC-MS" purity. The measurement range was 200-3000 m/z. Spectra were collected in positive ion mode.

*Purification and characterization of PNA conjugates.*

All the products obtained on solid support were purified after cleavage purified by preparative reversed-phase HPLC on a Vydac C18 column (22 mm x 9 250 mm), using the following solvent systems: S1 0.1% aqueous TFA, S2 80% acetonitrile + 0.1% TFA, linear

gradient from 5 to 70% of B for 50 min, flow rate 7.0 ml/min, UV detection at 254 nm. The resulting fractions were collected and subjected to a lyophilization process. The identities of the products were confirmed by MS analysis using the above-described Shimadzu IT-TOF mass spectrometer equipped with an electrospray (ESI) ionization source. The purity of peptides was analyzed using a Nexera (Shimadzu) HPLC system with a UV detection (PDA - 254 nm) and an Aeris Peptide XB-C18 column (100 mm × 2.1 mm) 3.6 μm bead diameter. The separation conditions were described in the LC-MS section.

*NMR analysis*

$^{1}H$ NMR, $^{13}C\{^{1}H\}$ NMR, and $^{77}Se$ NMR spectra were recorded on a high field Bruker 500 MHz spectrometer equipped with a broadband inverse gradient probe head. Spectra were referenced to the residual solvent signal ($CDCl_3$ at 7.24 ppm or MeOD at 4.87 ppm). The deuterated solvents were purchased from Sigma-Aldrich.

*PNA thermal stability analysis*

The melting temperatures of PNA conjugates were measures using UV-Vis Spectrophotometer (Agilent Technologies Cary 60 UV-Vis). Spectra were recorded in water from 400 to 200 nm, ramping every 2 $^{o}C$.

## SYNTHETIC PROTOCOLS

*Synthesis of 1,2-bis(4-methoxybenzyl)diselane*

(Reaction was carried out under nitrogen atmosphere) Absolute ethanol (30 mL) was added to selenium powder (0.6 g, 7.6 mmol) and sodium borohydride (0.2 g, 5.3 mmol) and the mixture was stirred in an ice bath until the vigorous reaction was complete. The reaction mixture was then heated under reflux for 1.5 h. 4-methoxybenzyl chloride (0.68 mL, 5.0 mmol) was added and the solution was allowed to reflux for 4 hours. The obtained solution was cooled down to room temperature, water was added and the aqueous layer was extracted with chloroform (2x). The organic layer was dried over anhydrous $MgSO_4$ and the solvent was removed on a rotary evaporator. (0.95 g, 95%)

*Synthesis of 2-(4-methoxybenzylseleno)ethylamine*

Sodium borohydride (0.4 g, 10.6 mmol) was added portion-wise to a solution of 1,2-bis(4-methoxybenzyl)diselane (1.0g, 2.5 mmol) in EtOH/DMF (30 mL, 1:1, v/v) and the reaction mixture was stirred for 2 hours at room temperature. 2-bromoethylamine hydrobromide (1.3 g, 6.25 mmol) was then dissolved in EtOH (5 mL) and added dropwise at 0 °C. The solution was warmed to room temperature and stirred overnight. The reaction was carried out under a nitrogen atmosphere. The solvent was removed under reduced pressure. The obtained product was dissolved in a saturated aqueous solution of $NaHCO_3$ and the aqueous layer was extracted with ethyl acetate (2x). The organic layer was washed with brine and dried over anhydrous $MgSO_4$. The solvent was removed on a rotary evaporator. (0.55 g, yield 90%)

**$^{1}$H NMR**: $^{1}$H NMR (500 MHz, $CDCl_3$, 300 K): δ 1,56 (s, 2H, H(1)), 2,58 (t, 2H, H(2)), 2,85 (k, 2H, H(3)), 3,75 (s, 2H, H(4)), 3,79 (s, 3H, H(5)), 6,82 (m, 2H, H(6)), 7,20 (m, 2H, H(7))

**$^{13}$C NMR** (126 MHz, CDCl3, 298 K ) δ (ppm) 25,03; 25,53; 40,64; 54,30; 113,52; 129,62; 131,31; 158,65

*Synthesis of (Se-Mob-2-selenoethyl)glycine*

Into a round-bottom flask was introduced 300 mg of *Se*-Mob-selenocysteamine obtained according to the procedure described in subsection 4.3.1, which was dissolved in 15 ml of methanol. Then 102 mg of glyoxalic acid (1.1 mmol) was added. The resulting solution was stirred on a magnetic stirrer. In the next step, sodium cyanoborohydride was added, and stirring continued for 30 minutes. After this time, the solvent was evaporated to dryness in a stream of nitrogen. The final product obtained was subjected to MS analysis and used directly in the next step without purification.

**TLC**: $R_{f:}$ 0,15 (3% MeOH in $CHCl_3$)

**ESI-MS** - *m/z* $[M+H]^+$: 304,0500, calculated for *m/z* $[M+H]^+$: 304,0446

*Synthesis of N-Fmoc-(Se-Mob-2-selenoethyl)glycine*

The product obtained in the previous step was dissolved in 7 ml of distilled water and 8 ml of acetone, and then 402 $\mu$l DIPEA was added. Stirring the solution on a magnetic stirrer, 373 mg of Fmoc-OS dissolved in 5 ml of acetone was then added. The resulting solution was stirred overnight. After this time, the acetone was evaporated in a stream of nitrogen, after which 8 ml of distilled water was added. The solution was acidified with potassium bisulfate (VI) ($KHSO_4$) to pH 3 and then extracted 3 times with ethyl acetate (15ml). The collected organic layers were washed with brine and then scrubbed over anhydrous magnesium sulfate. After filtering the drying agent, the solvent was evaporated in a stream of nitrogen. Flash chromatography with the usage of silica gel (12g column) was used to purify the crude product. The oil was dissolved in 2 ml of chloroform, and a mixture consisting of 1% methanol in chloroform (+ 0.1% acetic acid) was used as the eluent. The fractions were controlled at 254 nm. The obtained final product was analyzed by MS, NMR, and HPLC analysis to confirm its structure and purity.

**TLC**: $R_f$: 0,25 (3% MeOH in $CHCl_3$)

**ESI-MS:** *m/z* $[M+Na]^+$: 548,0915, calculated for *m/z* $[M+Na]^+$: 548,0946

**ESI-MS/MS**: *m/z* $[M+H]^+$: 297,9960, calculated *m/z* $[M+H]^+$: 298,1073; m/z $[M+Na]^+$: 326,0272, calculated *m/z* $[M+Na]^+$: 326,0259.

**HPLC**: 13,7 min (gradient 5-90%B in 15min)

**$^1$H NMR** (500 MHz, $CDCl_3$, 300 K): δ 2,46 (t, 2H, H(1)), 3,26 (t, 2H, H(2)), 3,63 (s, 3H, H(3)), 3,76 (s, 2H, H(4)), 3,85 (s, 2H, H(5)), 4,23 (t, 1H, H(6)), 4,45 (d, 2H, H(7)), 6,79 (m, 2H, H(8)), 7,19 (m, 2H, H(9)), 7,52 (d, 8H, H(10)).

**$^{13}$C NMR** (126 MHz, CDCl3, 298 K ) δ (ppm) 20,98; 26,58; 47,07; 49,26; 50,74; 55,06; 67,36; 113,76; 120,06; 124,75;127,08; 127,79, 129,96; 141,35; 143,73; 155.38; 156,27; 158,58; 173,95

# ANALYTICAL DATA

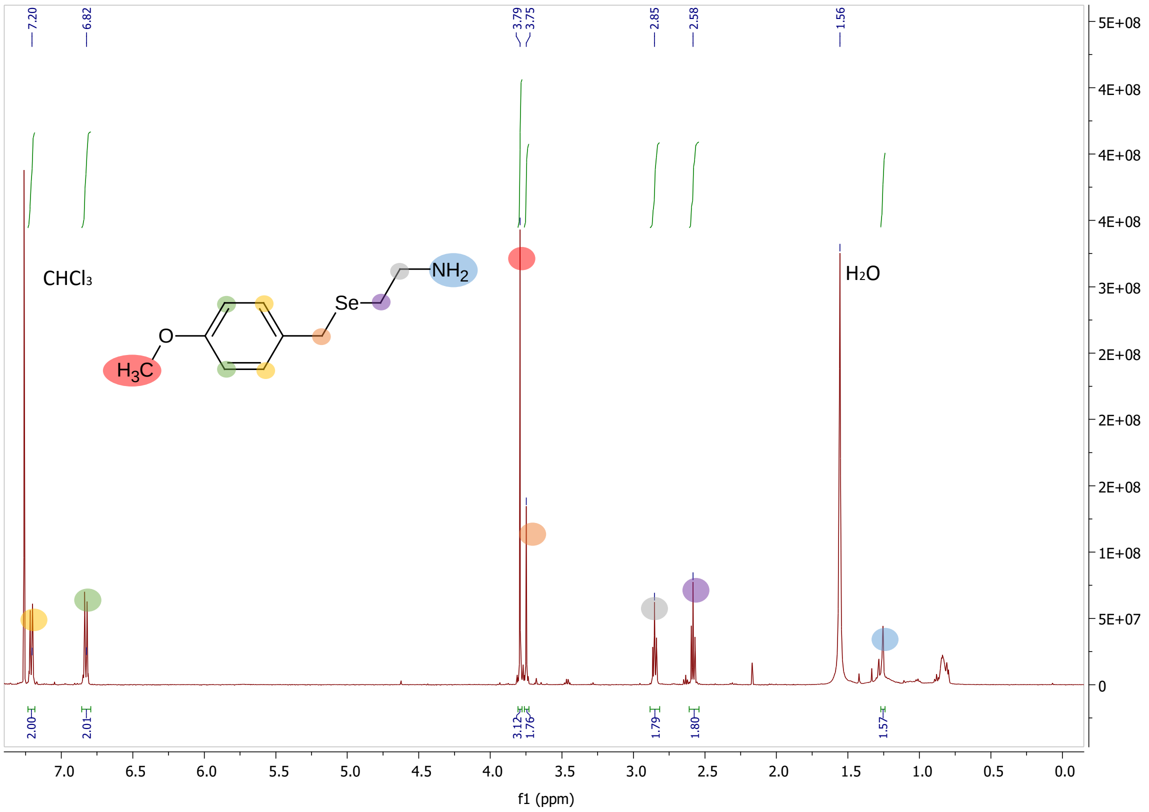


*Fig. S 1 $^{1}$H NMR ($CD_3OD$, 500 MHz, 300 K) spectrum of 2-(4-methoxybenzylseleno)ethylamine*

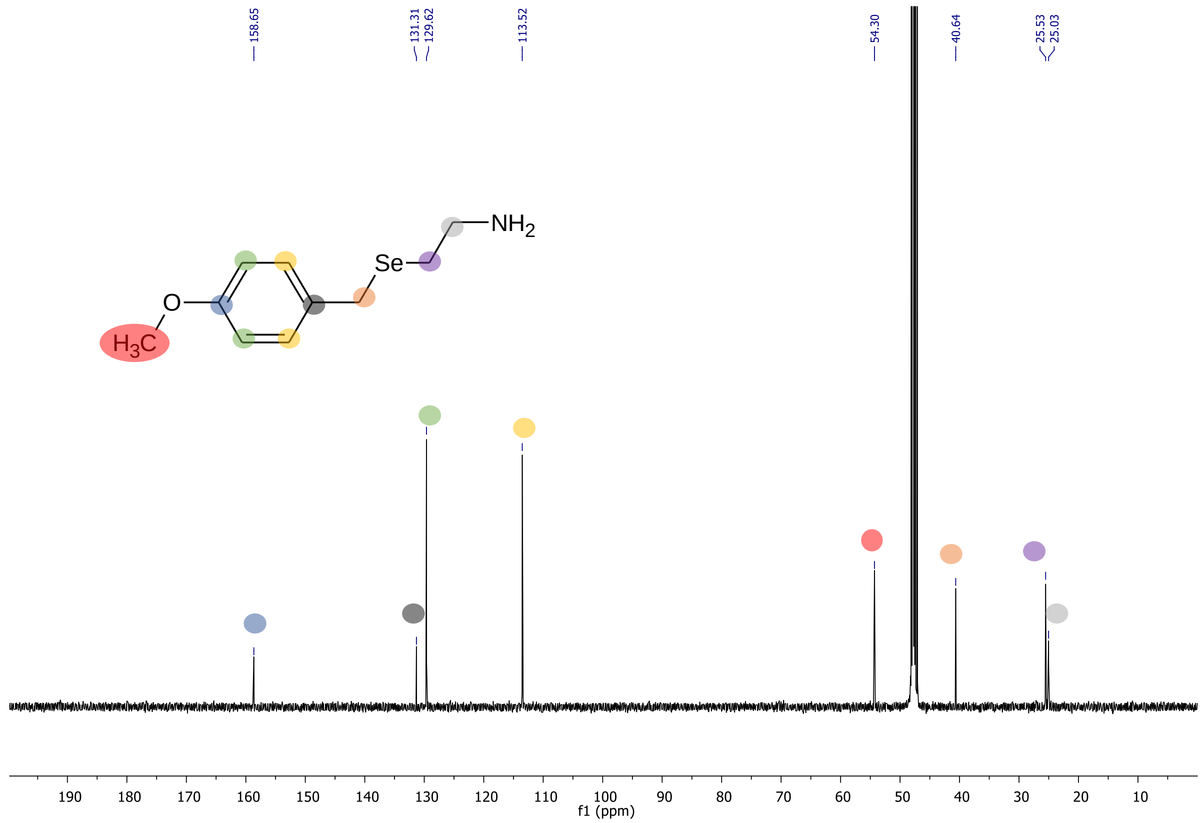


*Fig. S 2* $^{13}$*C NMR (CD3OD, 126 MHz, 300 K) spectrum of 2-(4-methoxybenzylseleno)ethylamine*

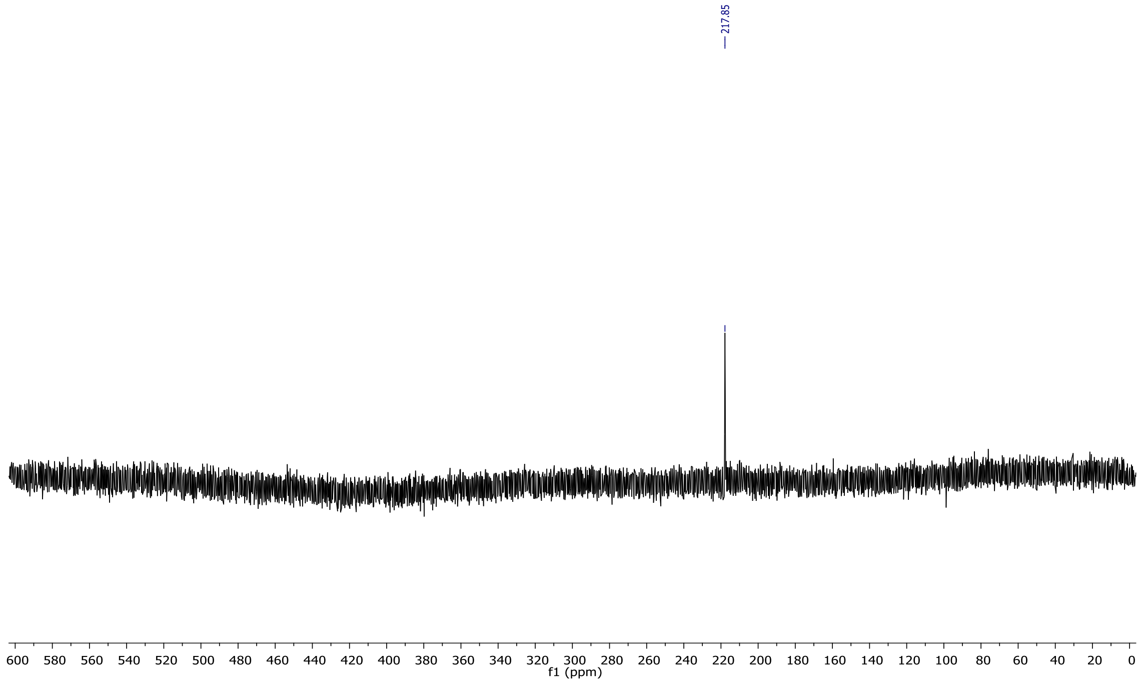


*Fig. S 3* $^{77}$*Se NMR (CD3OD, 114 MHz, 300 K) spectrum of 2-(4-methoxybenzylseleno)ethylamine*

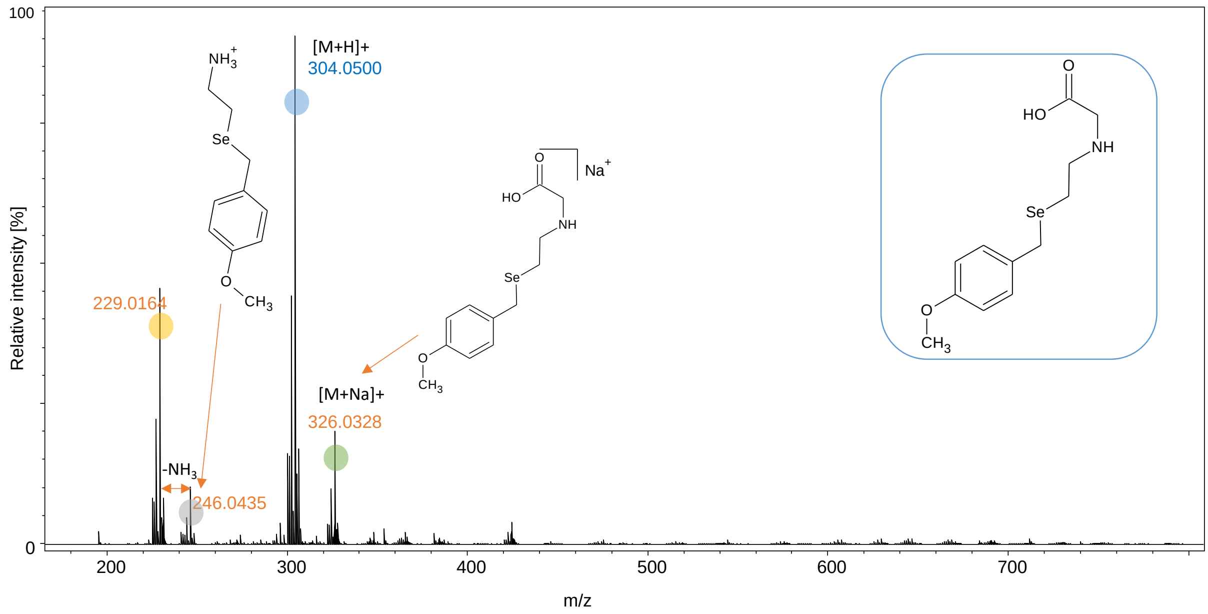


*Fig. S 4. ESI-MS spectrum of N- (Se-Mob-2-selenoethyl)glycine obtained after reductive amination*

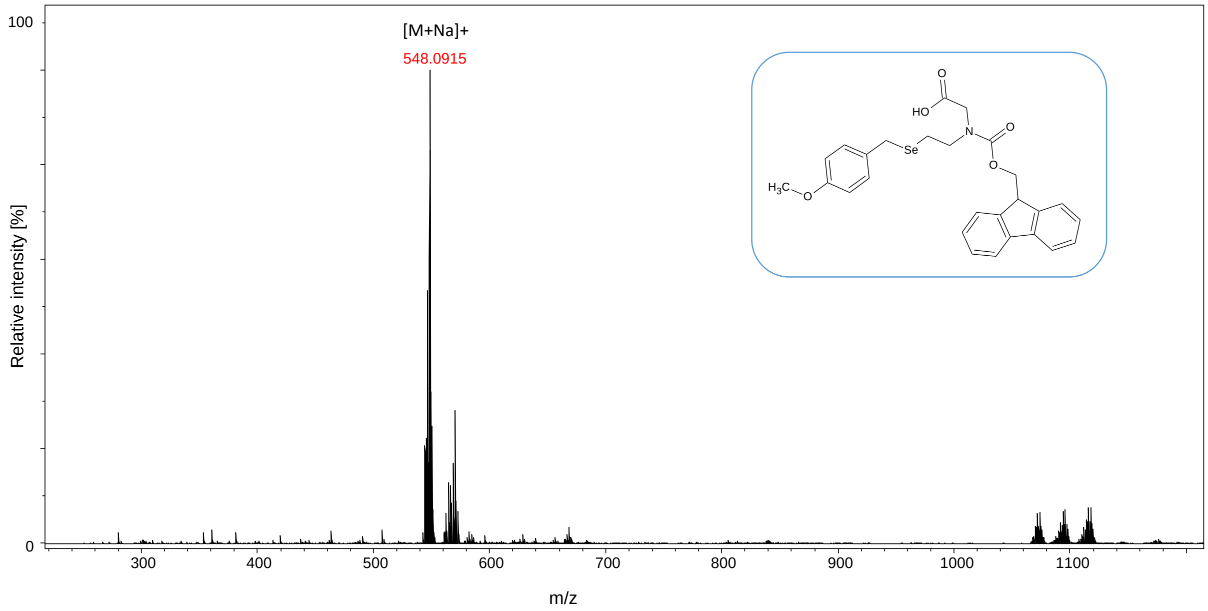


*Fig. S 5. ESI-MS spectrum obtained for purified N-Fmoc-(Se-Mob-2-selenoethyl)glycine*

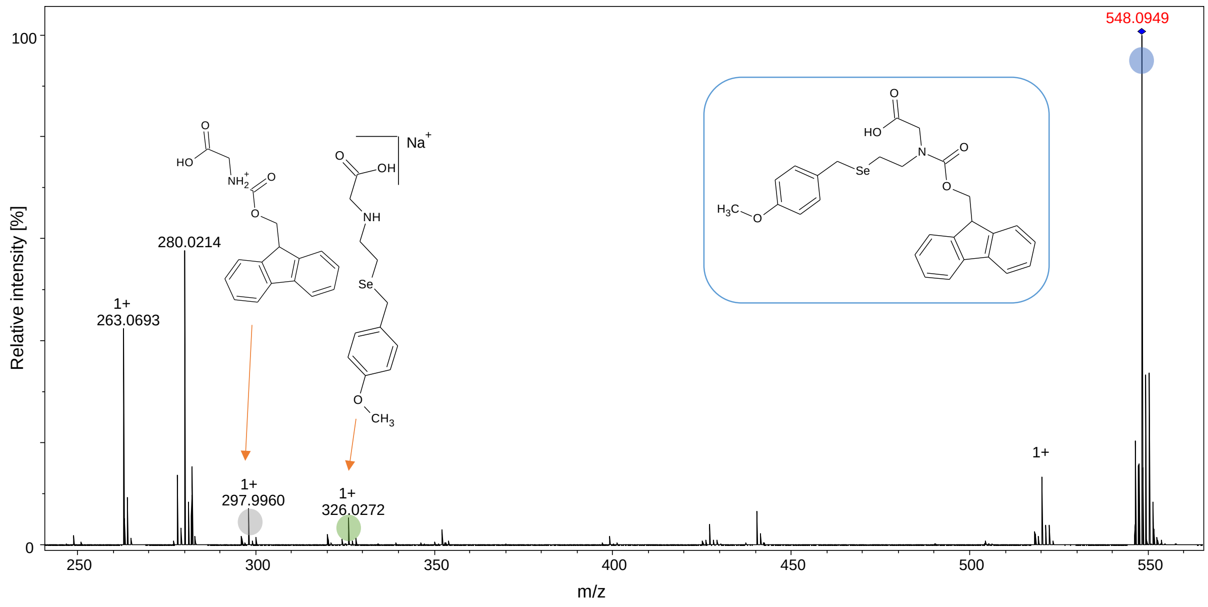


*Fig. S 6. ESI-MS/MS spectrum obtained for purified N-Fmoc-(Se-Mob-2-selenoethyl)glycine*

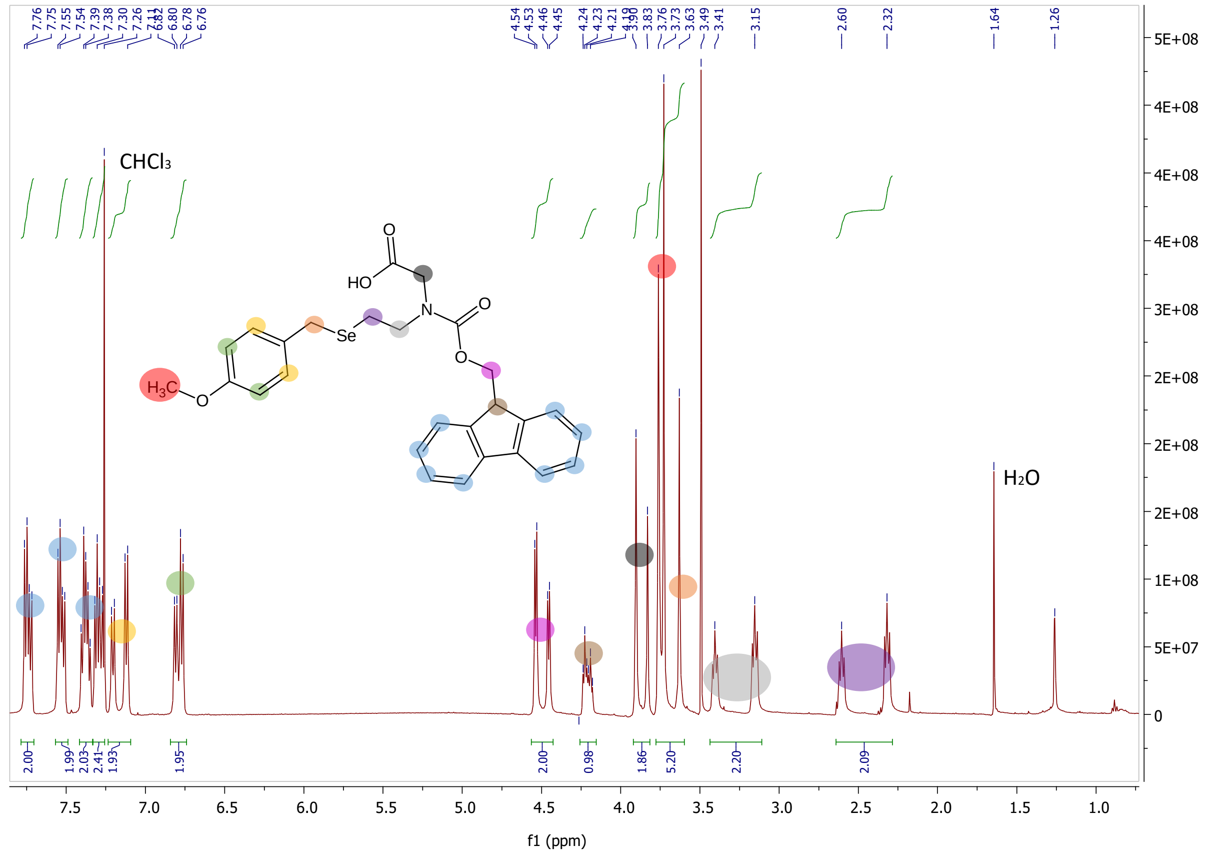


*Fig. S 7 $^{1}$H NMR ($CDCl_3$) 500 MHz, 300 K) spectrum obtained for purified N-Fmoc-(Se-Mob-2-selenoethyl)glycine*

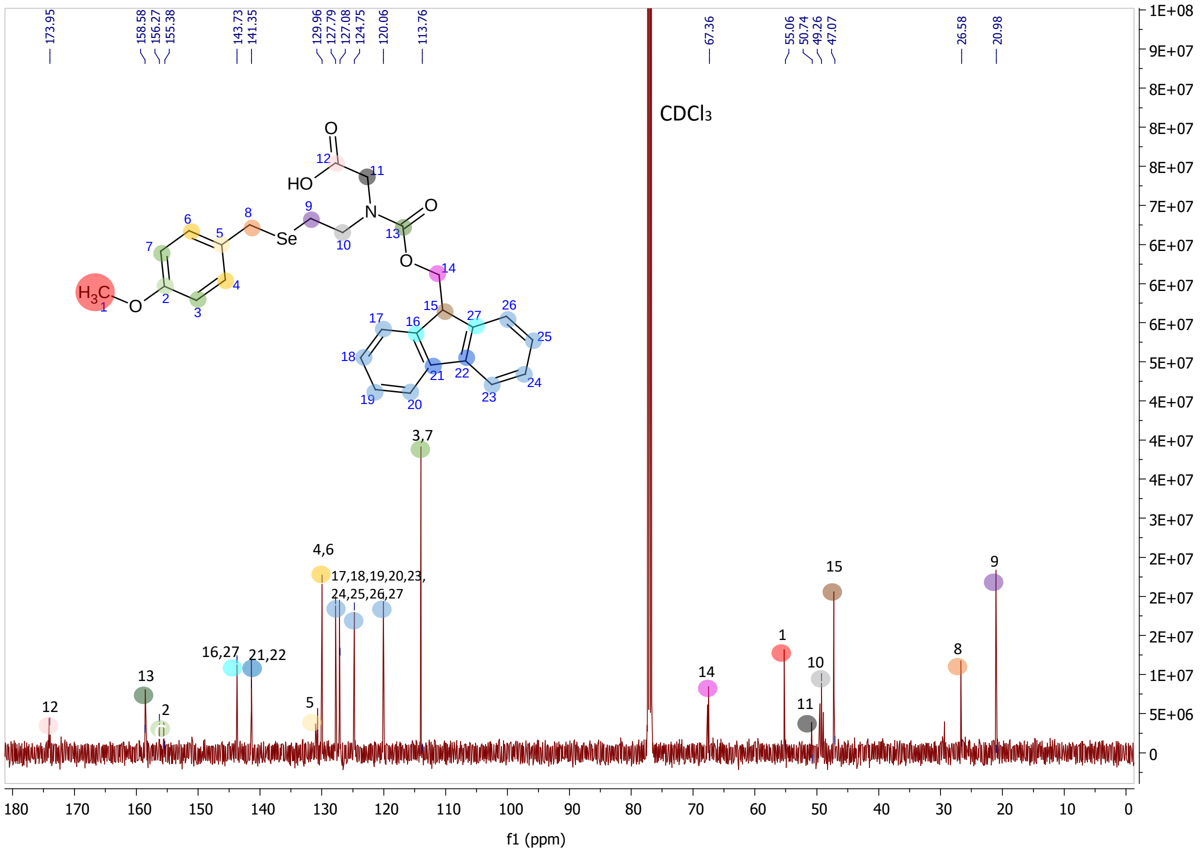

*Fig. S 8 $^{13}$C NMR ($CDCl_3$, 126 MHz, 300 K) spectrum obtained for purified N-Fmoc-(Se-Mob-2-selenoethyl)glycine*

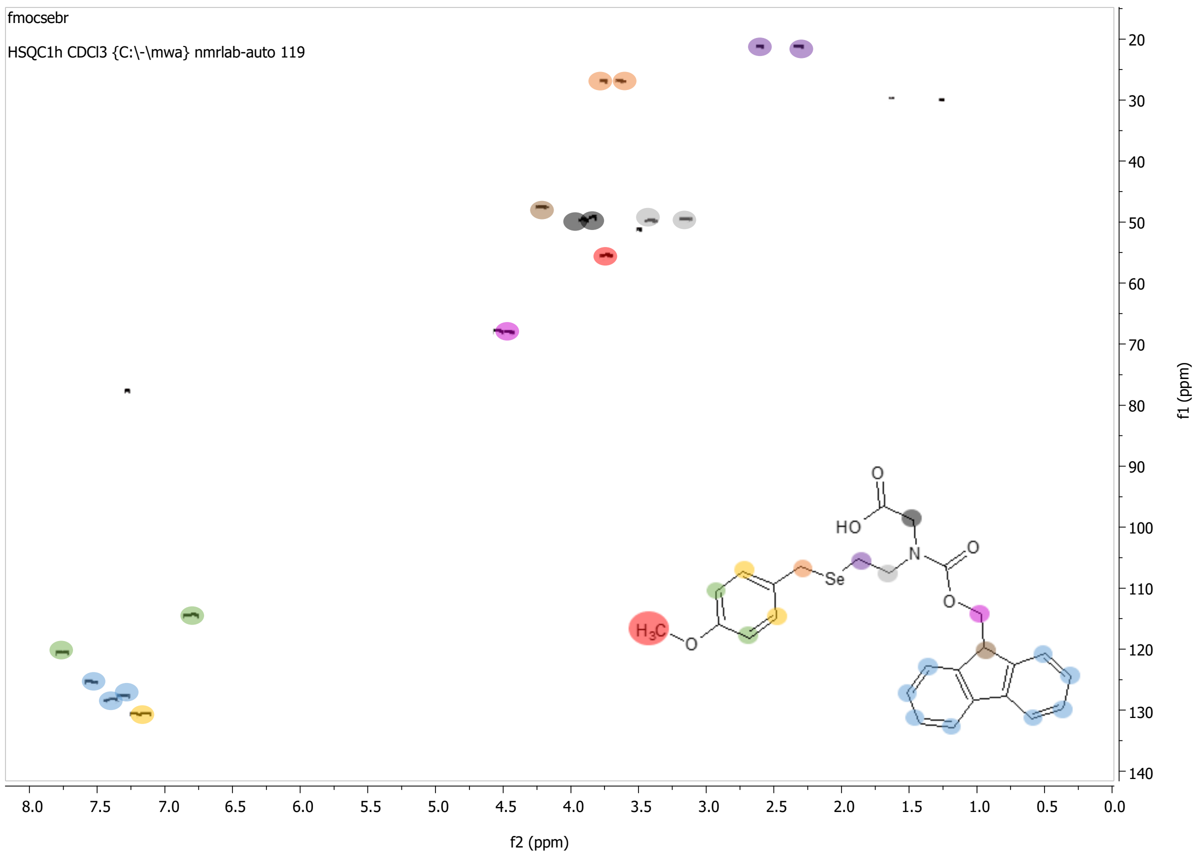


*Fig. S 9  2D HSQC NMR spectrum obtained for purified N-Fmoc-(Se-Mob-2-selenoethyl)glycine*

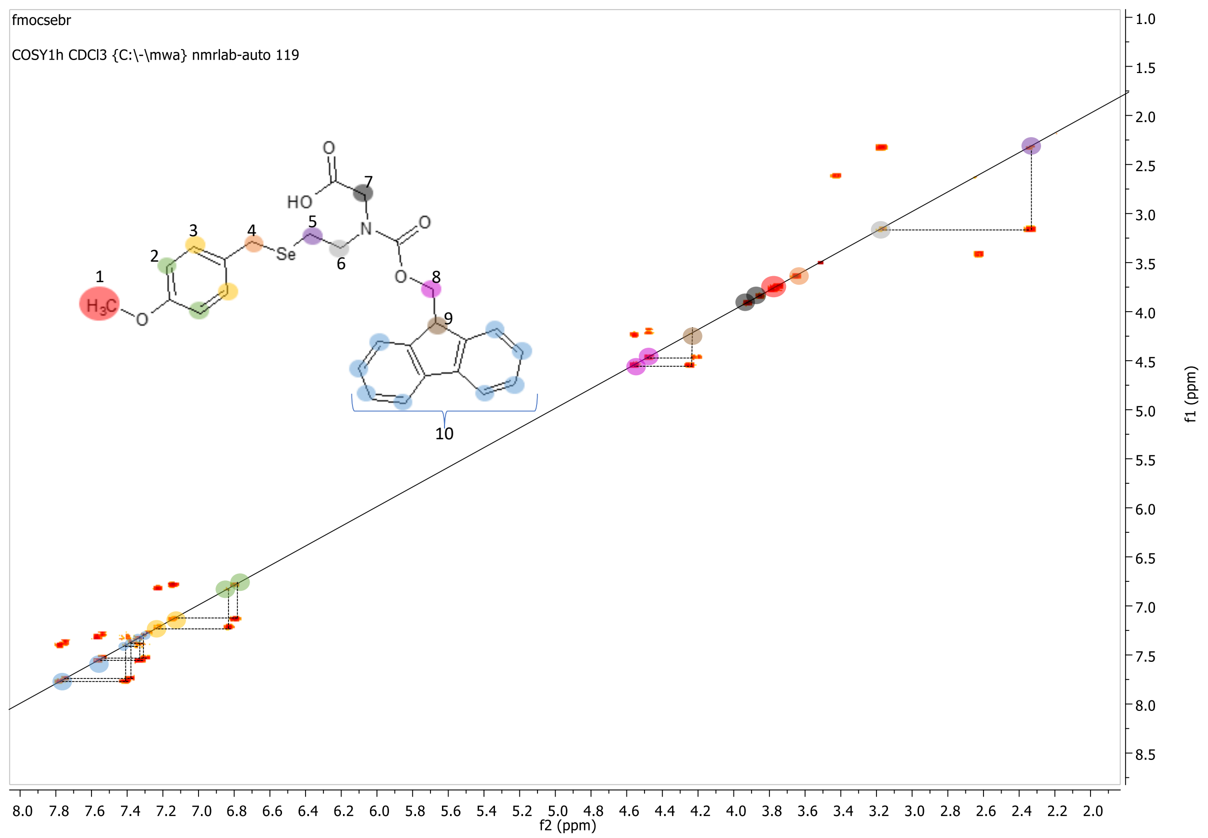


*Fig. S 10 2D COSY NMR spectrum obtained for purified N-Fmoc-(Se-Mob-2-selenoethyl)glycine*

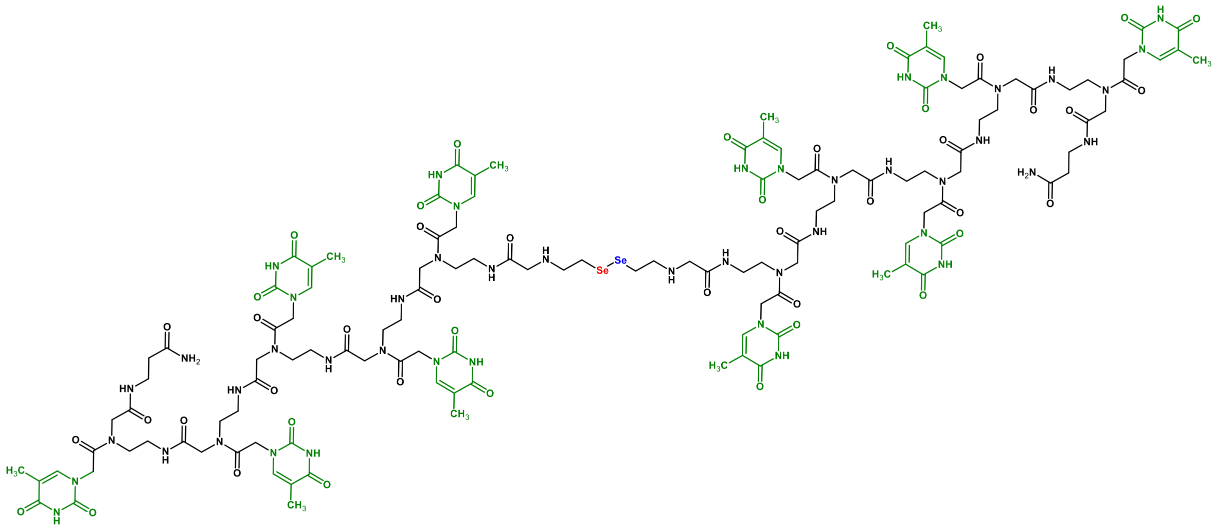

*Fig. S 11 Structure of PNA conjugate 1.1*

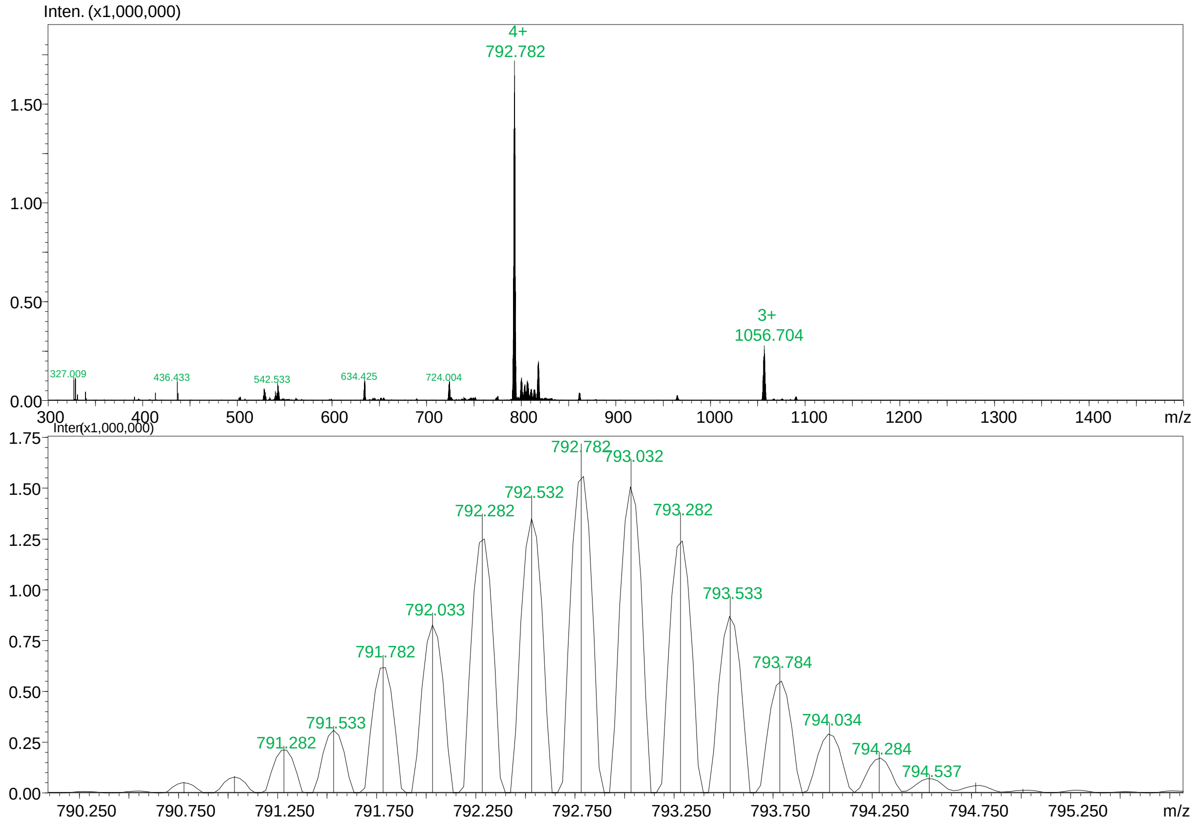


*Fig. S 12 ESI-MS spectrum obtained for PNA 1.1*

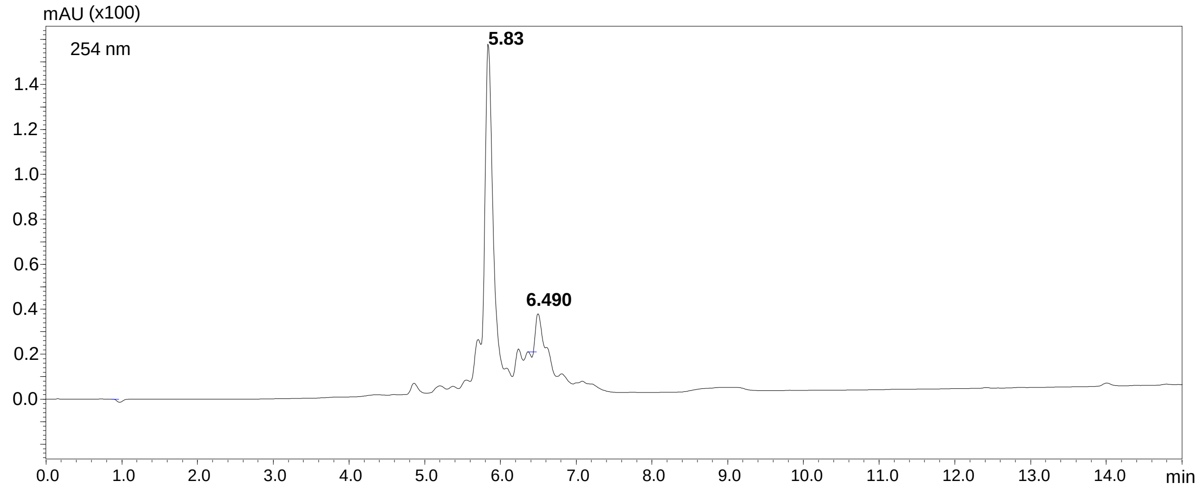


*Fig. S 13 HPLC chromatogram obtained for PNA 1.1*

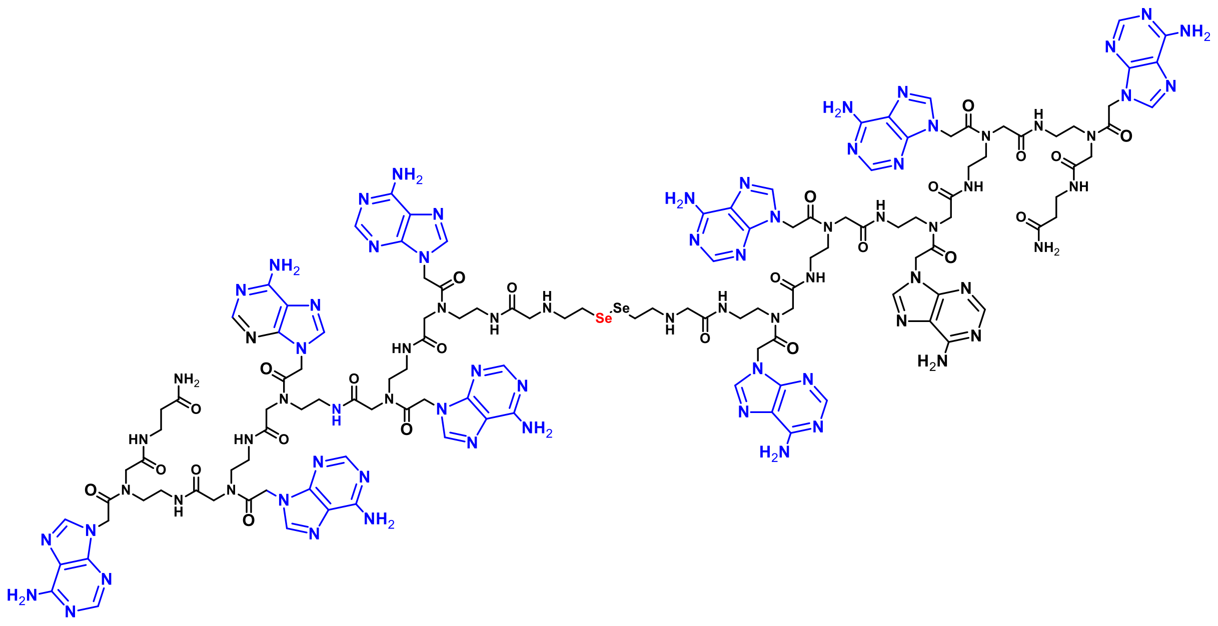

*Fig. S 14 Structure of PNA conjugate 1.2*

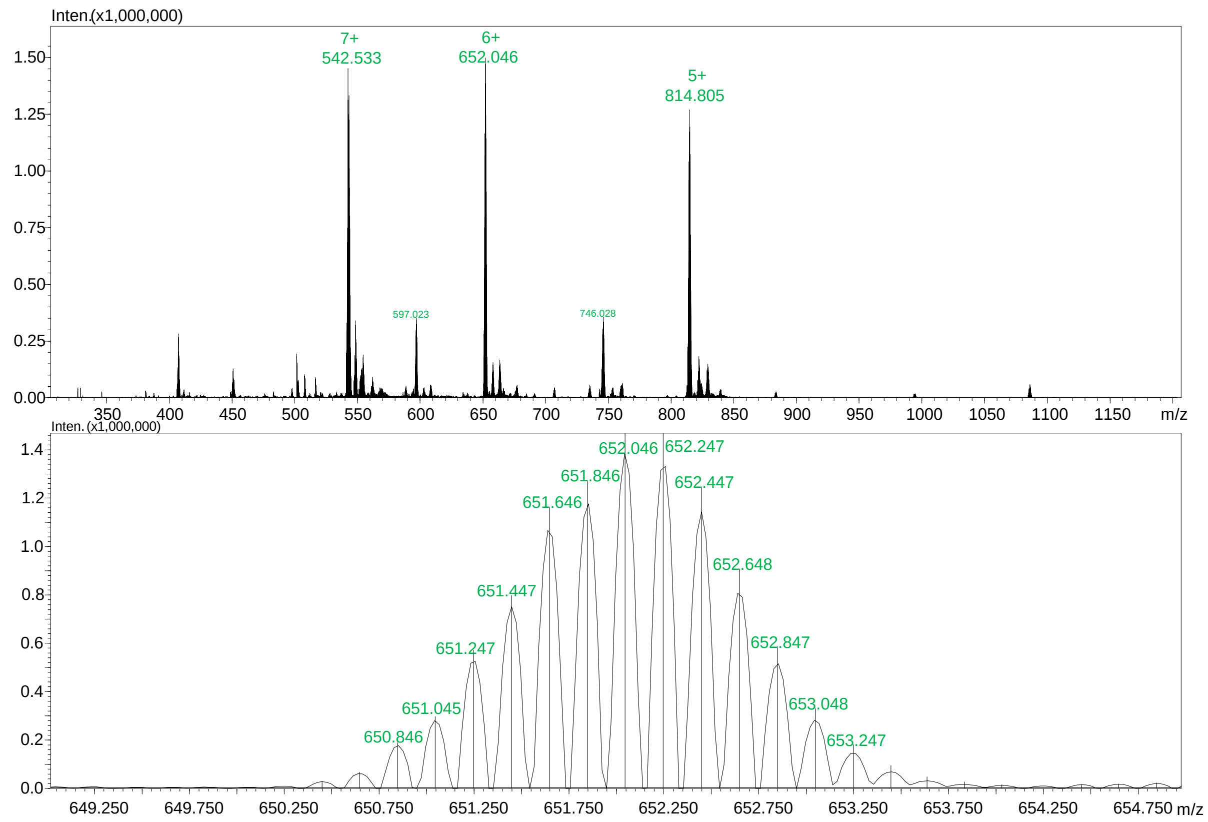


*Fig. S 15 ESI-MS spectrum obtained for PNA 1.2*

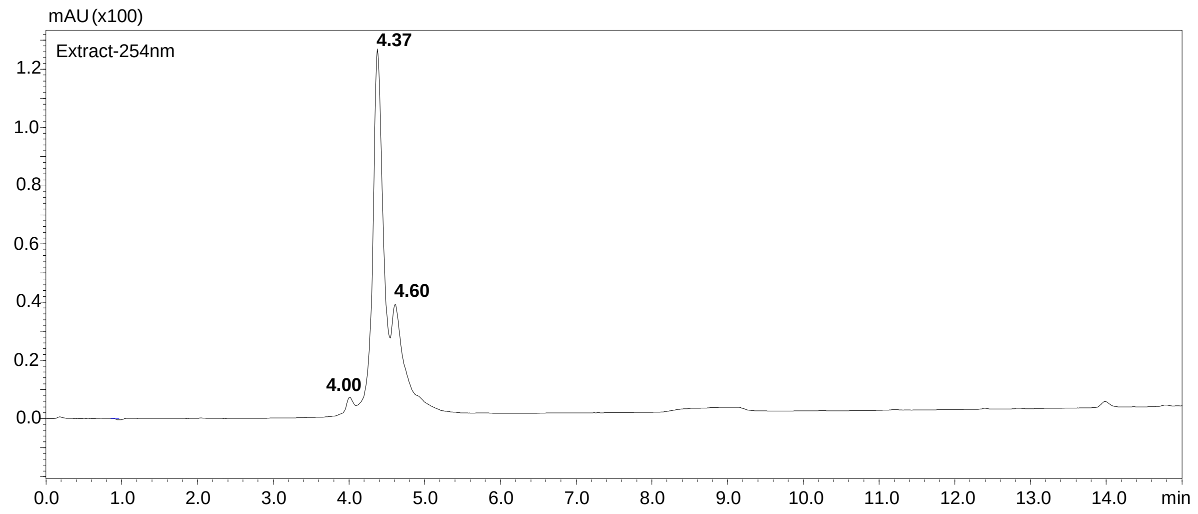


*Fig. S 16 HPLC chromatogram obtained for PNA 1.2*

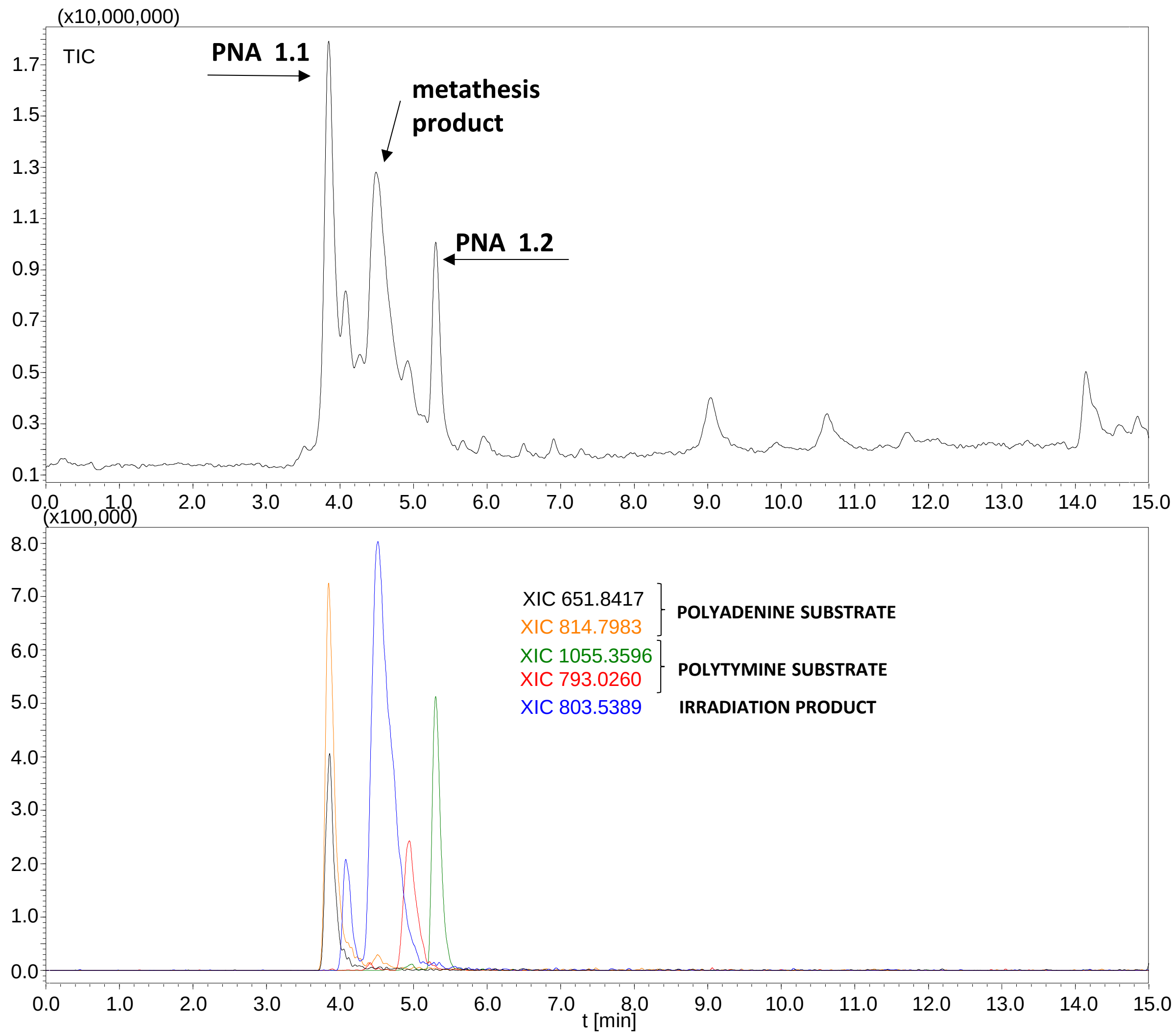


*Fig. S 17 LC-MS spectrum (TIC and XIC) obtained after irradiation of PNA conjugates 1.1 and 1.2 after 15 min of irradiation.*

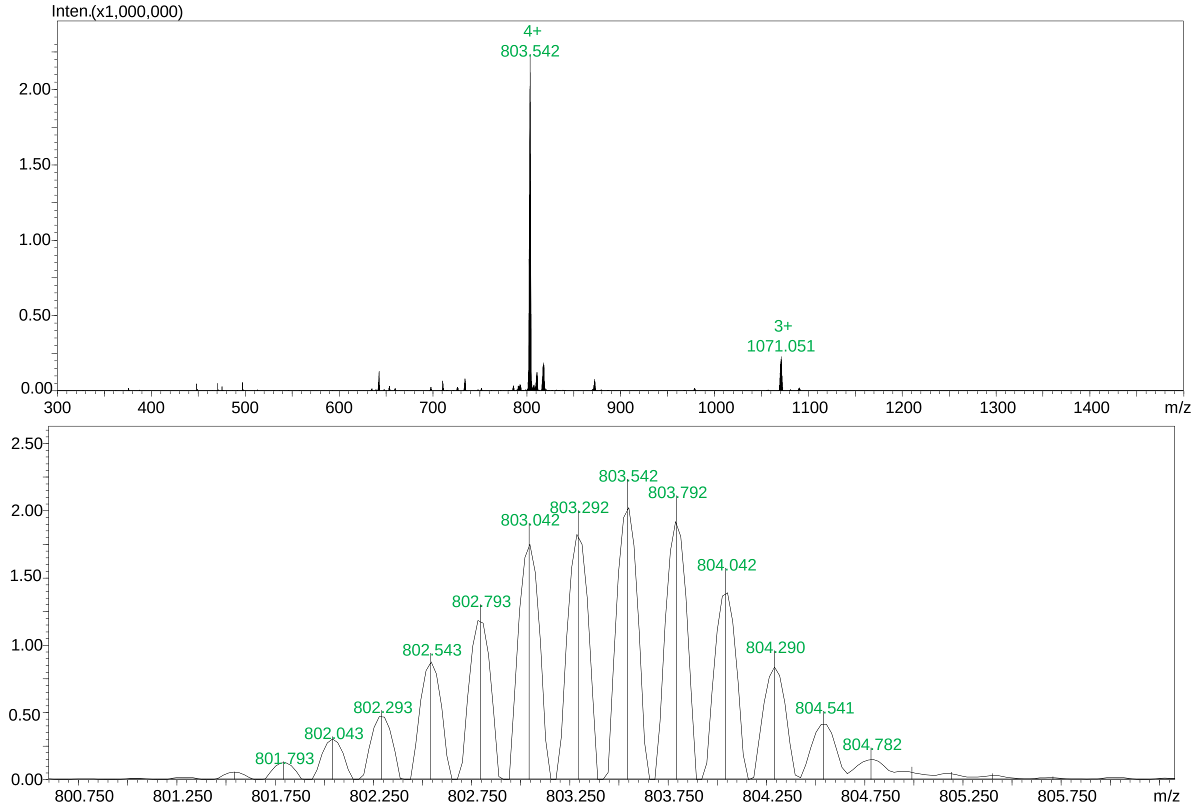


*Fig. S 18 ESI-MS spectrum obtained for the product after irradiation of PNA conjugates 1.1 and 1.2.*

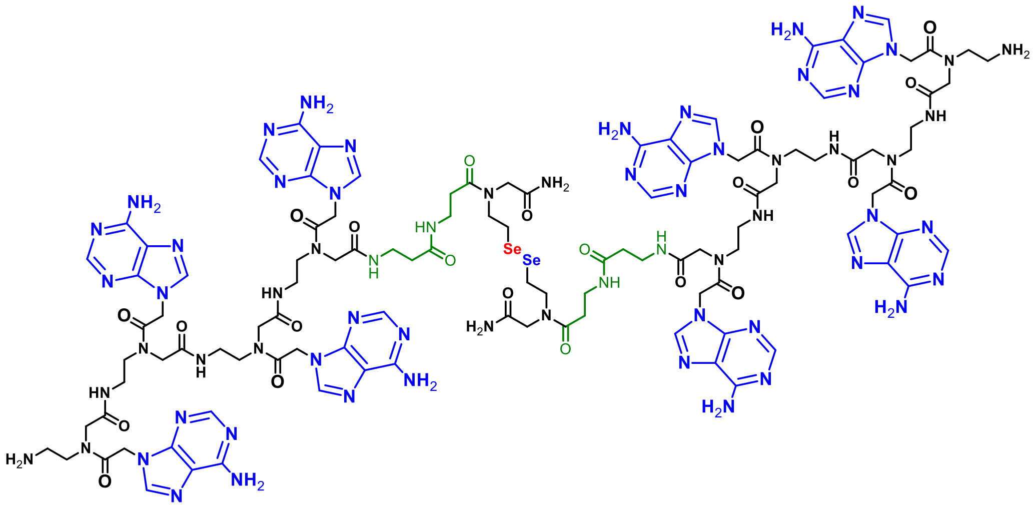

*Fig. S 19. Structure of PNA conjugate 2.1*

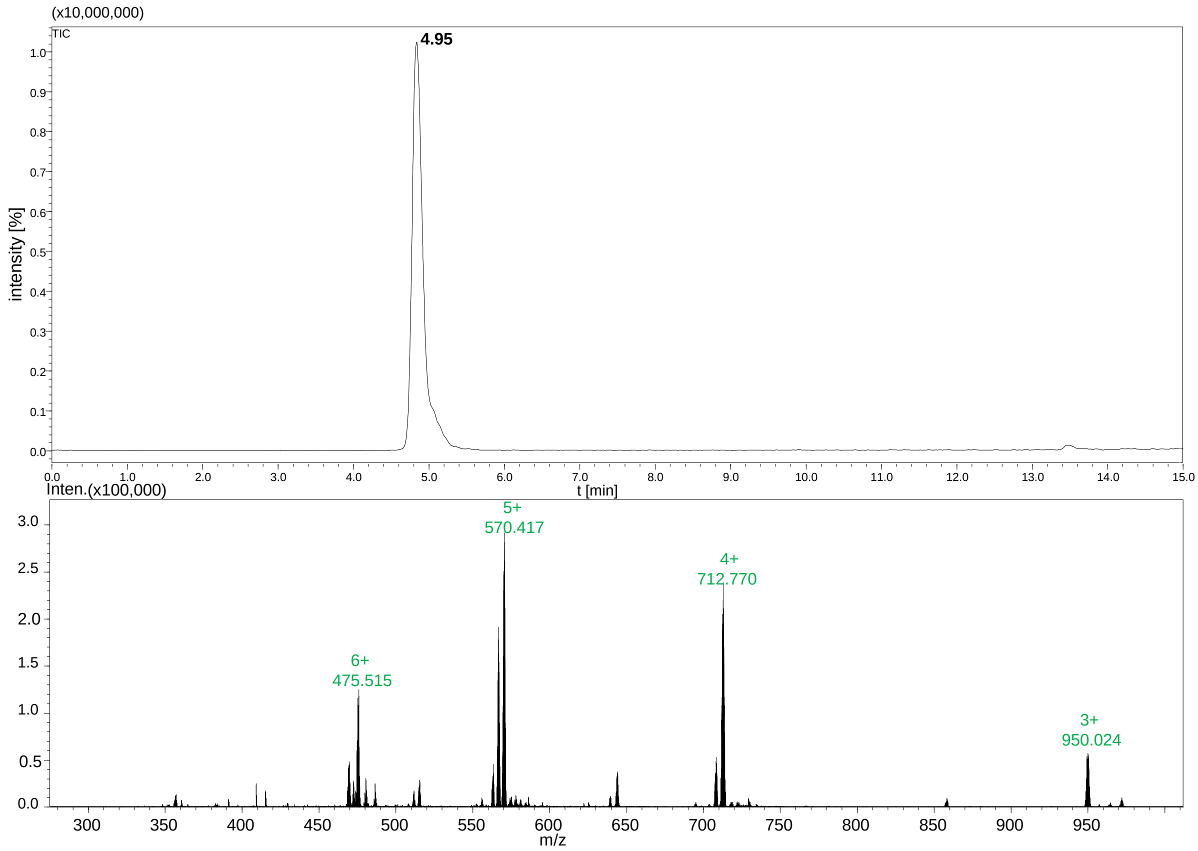


*Fig. S 20 LC-MS chromatogram and spectrum obtained for PNA conjugate 2.1*

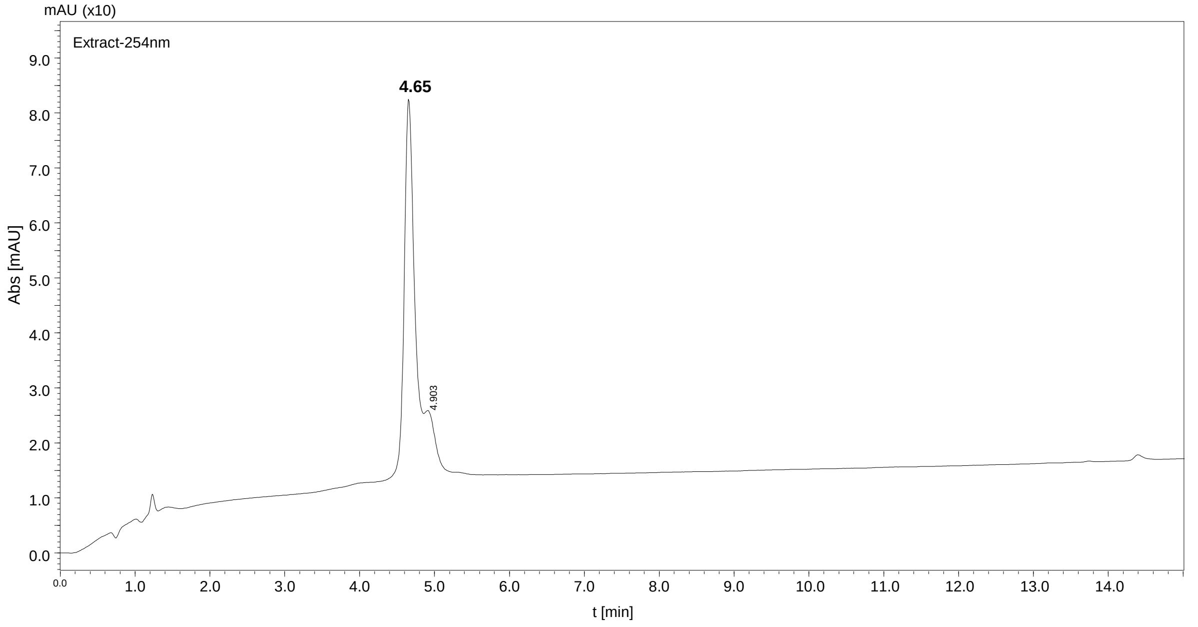


*Fig. S 21 HPLC chromatogram obtained for PNA conjugate 2.1*

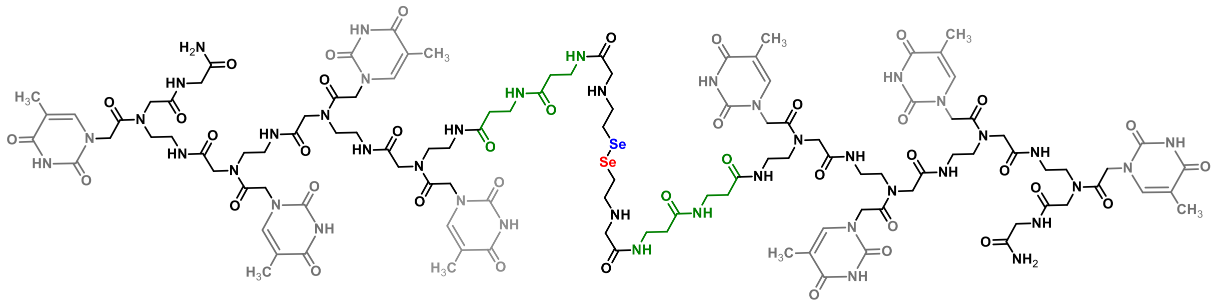

*Fig. S 22 Structure of PNA conjugate 2.2*

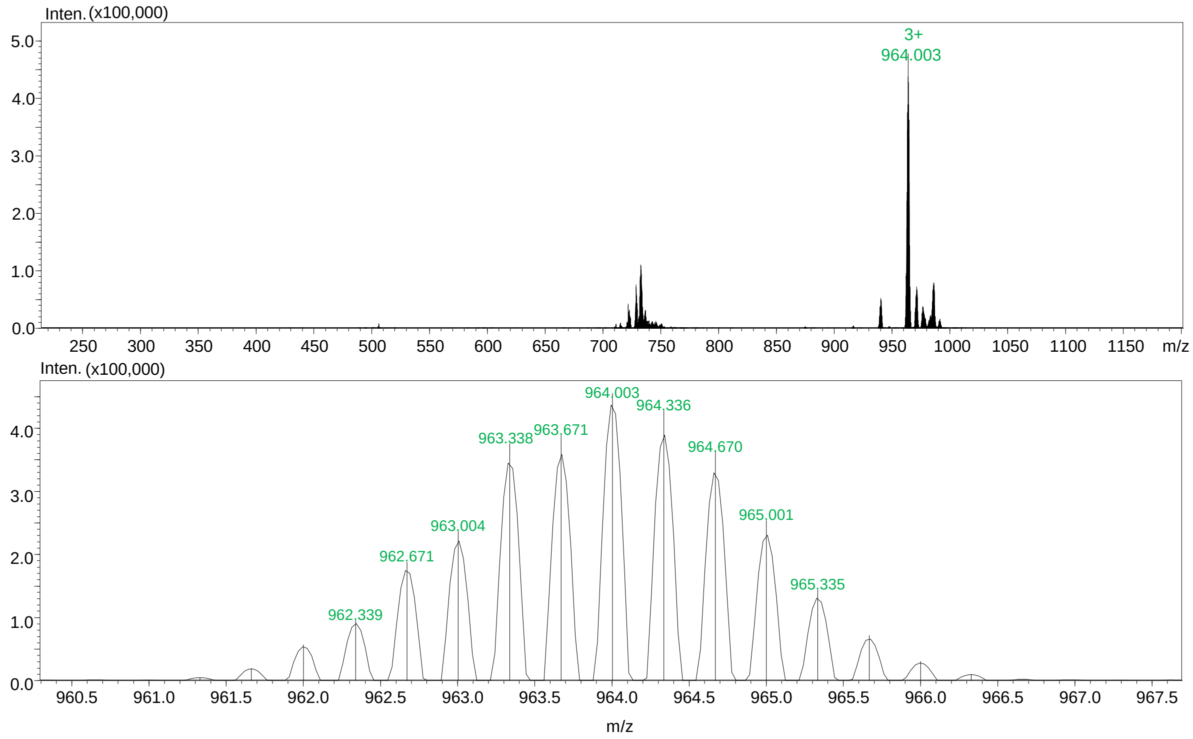


*Fig. S 23 ESI-MS spectrum obtained for PNA conjugate 2.2*

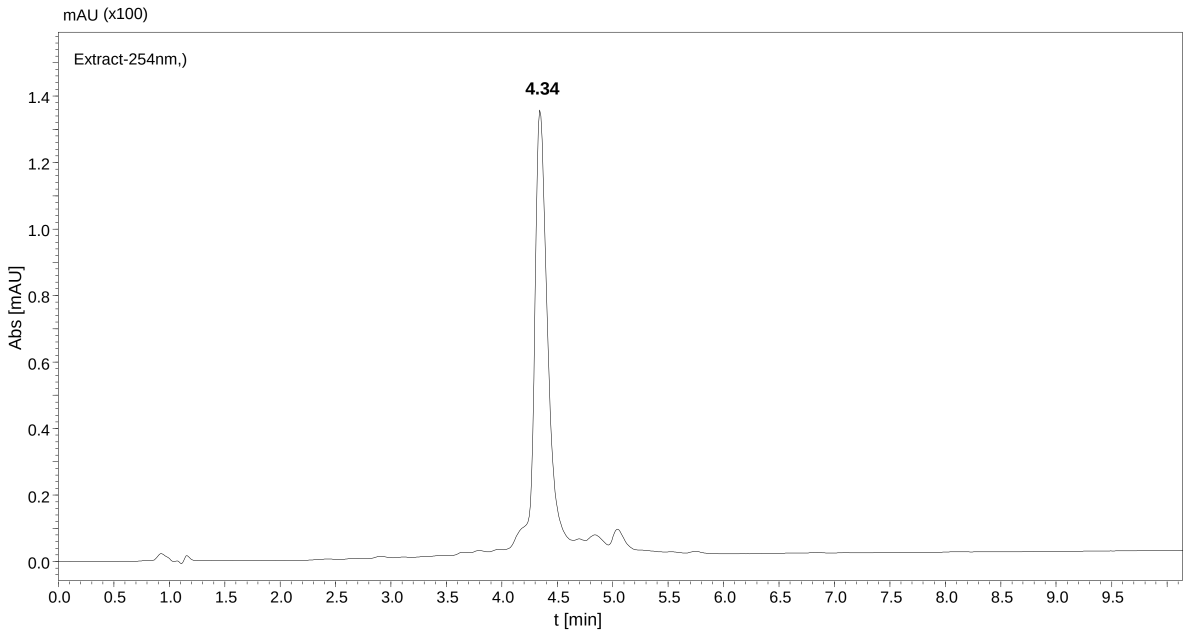


*Fig. S 24 HPLC chromatogram obtained for PNA conjugate 2.2*

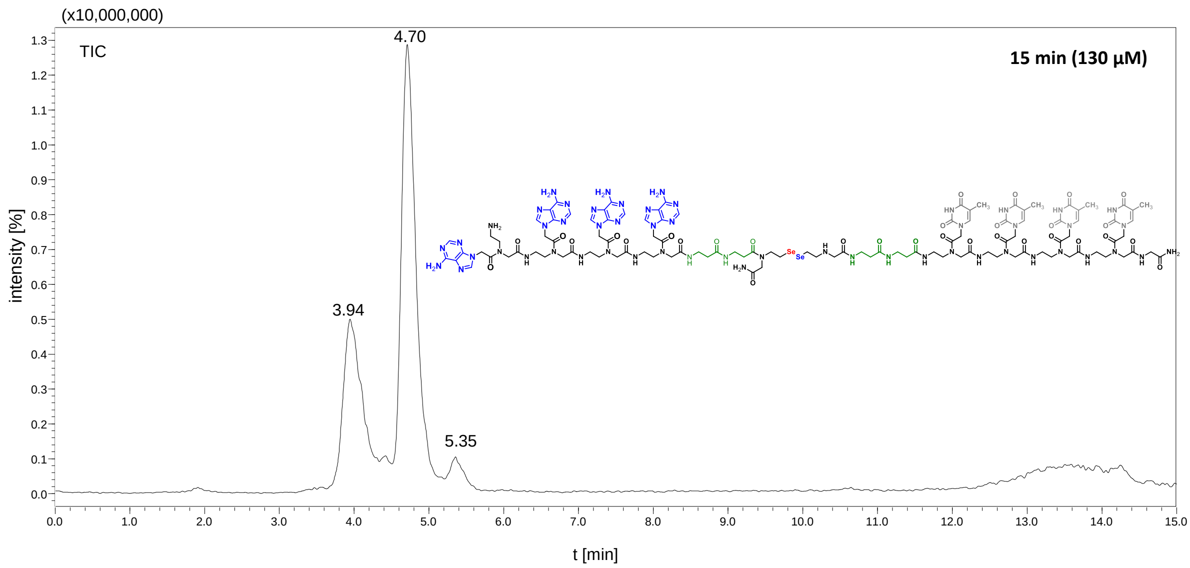


*Fig. S 25 HPLC chromatogram of the mixture obtained after visible-light irradiation of PNA conjugate 2.1 and 2.2 with concentrations of substrates of 130 µM*

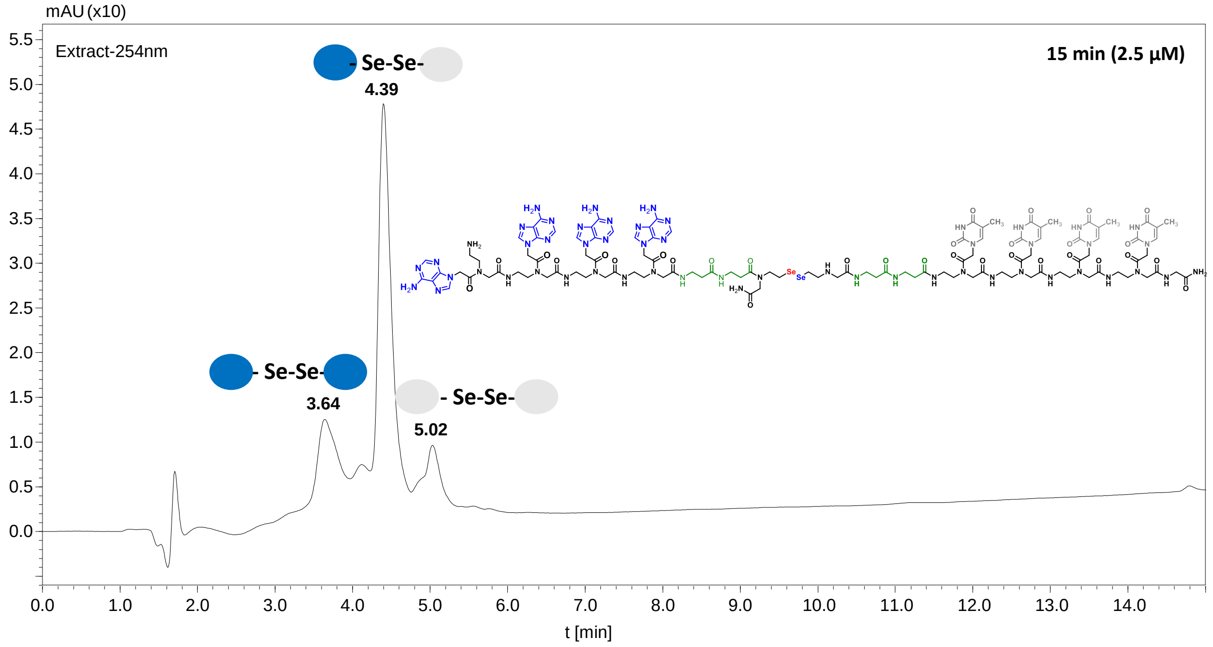


*Fig. S 26 HPLC chromatogram of the mixture obtained after visible-light irradiation of PNA conjugate 2.1 and 2.2 with concentrations of substrates of 2.5 µM*

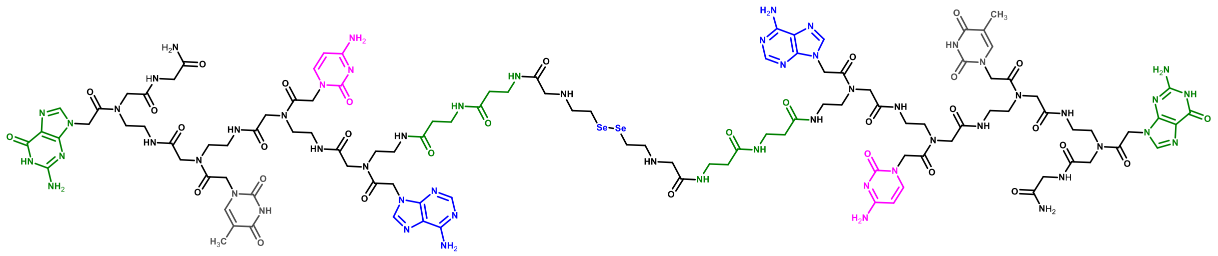

*Fig. S 27 Structure of PNA conjugate 3.1*

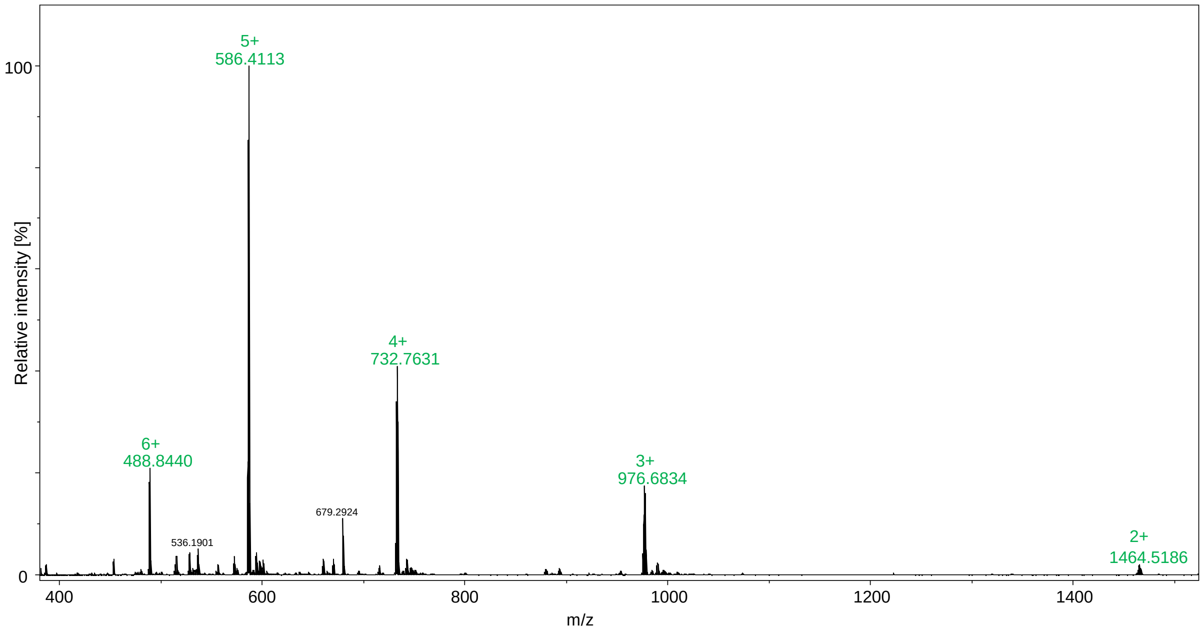


*Fig. S 28 ESI-MS spectrum obtained for PNA conjugate 3.1*

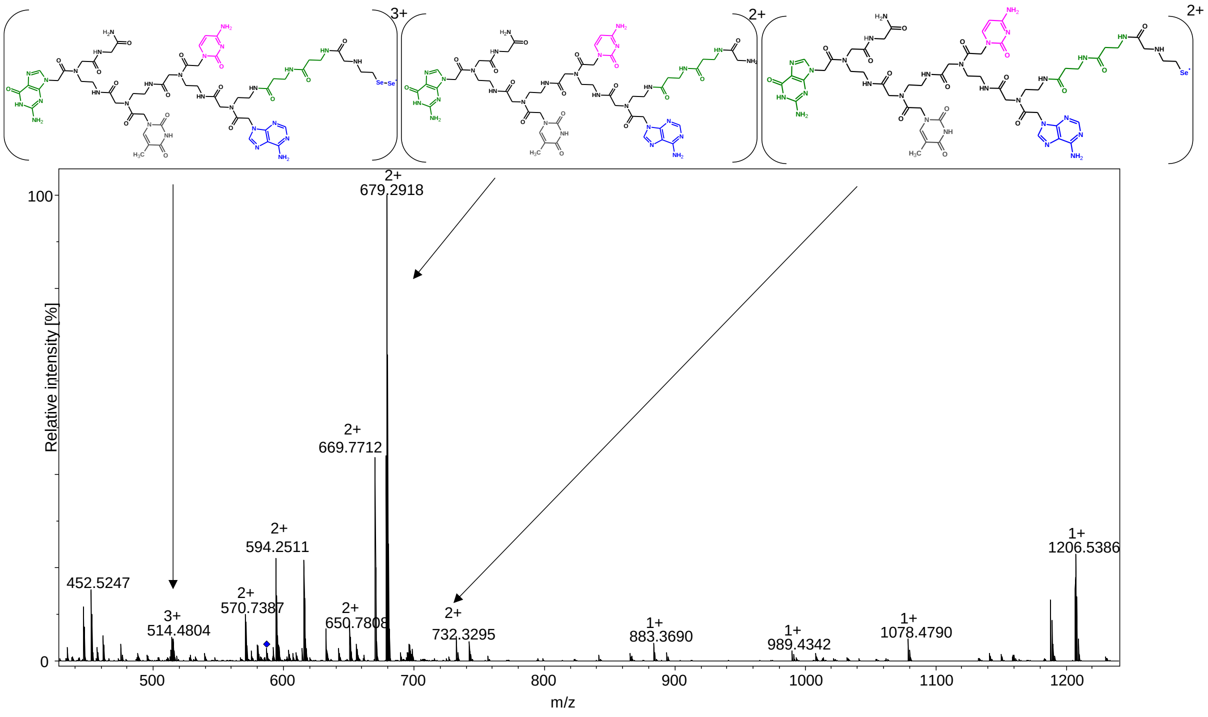


*Fig. S 29 ESI-MS/MS (CE 20 eV) spectrum obtained for PNA conjugate 3.1*

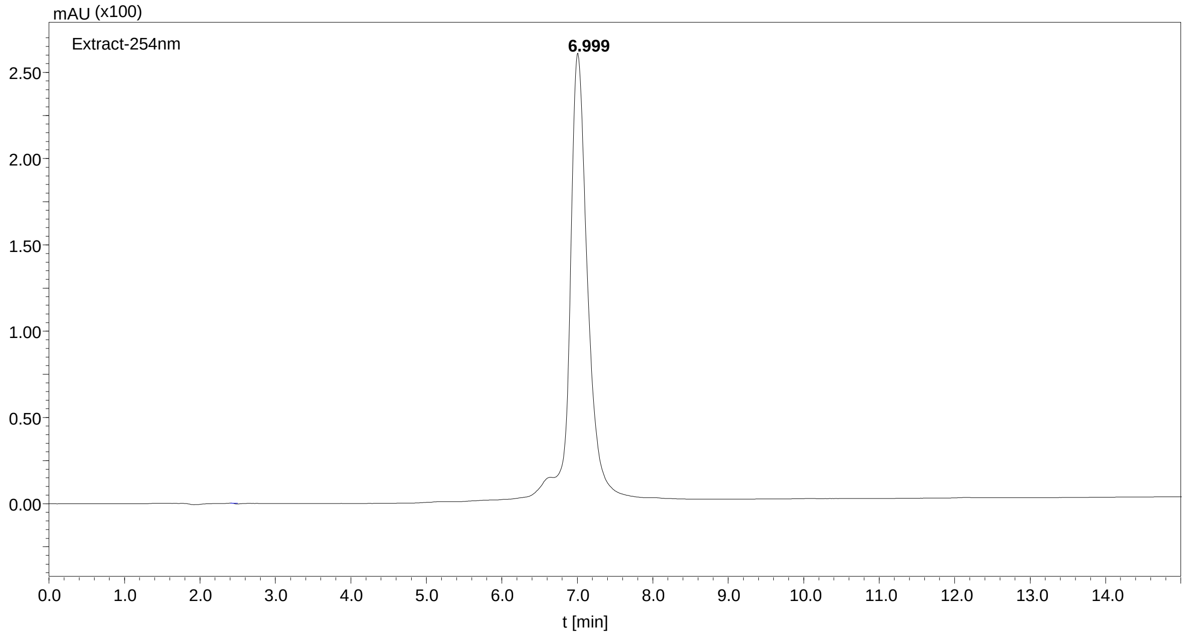


*Fig. S 30 HPLC chromatogram obtained for PNA conjugate 3.1*

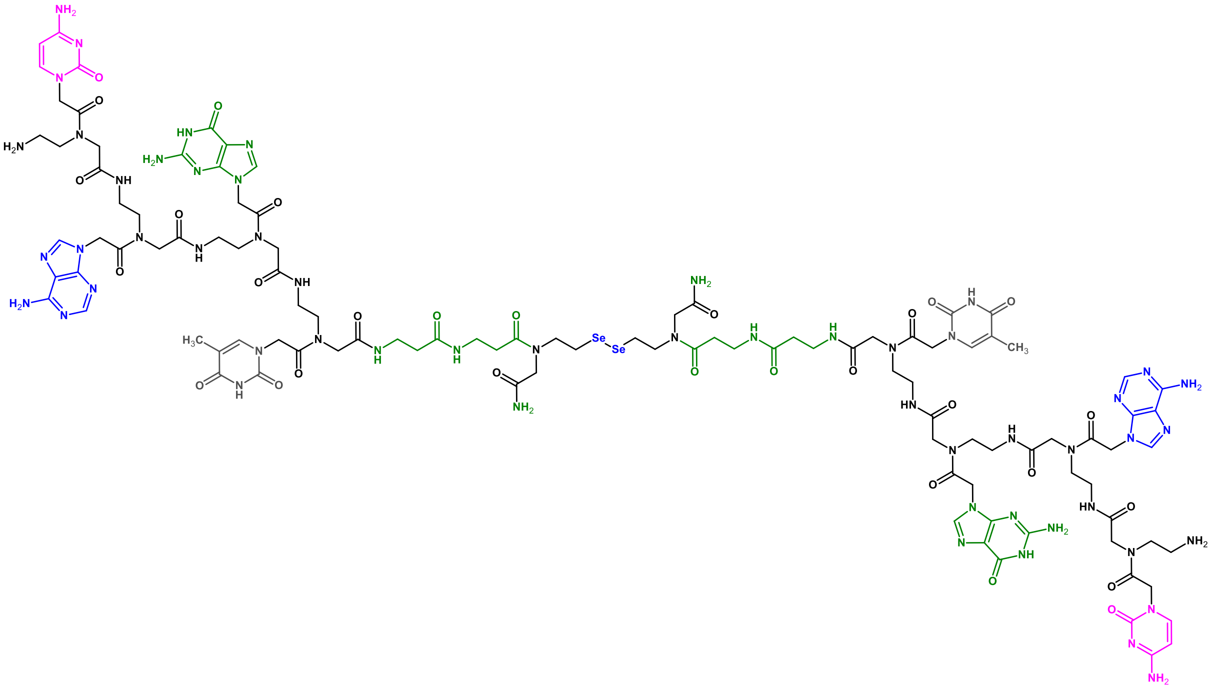

*Fig. S 31 Structure of PNA conjugate 3.2*

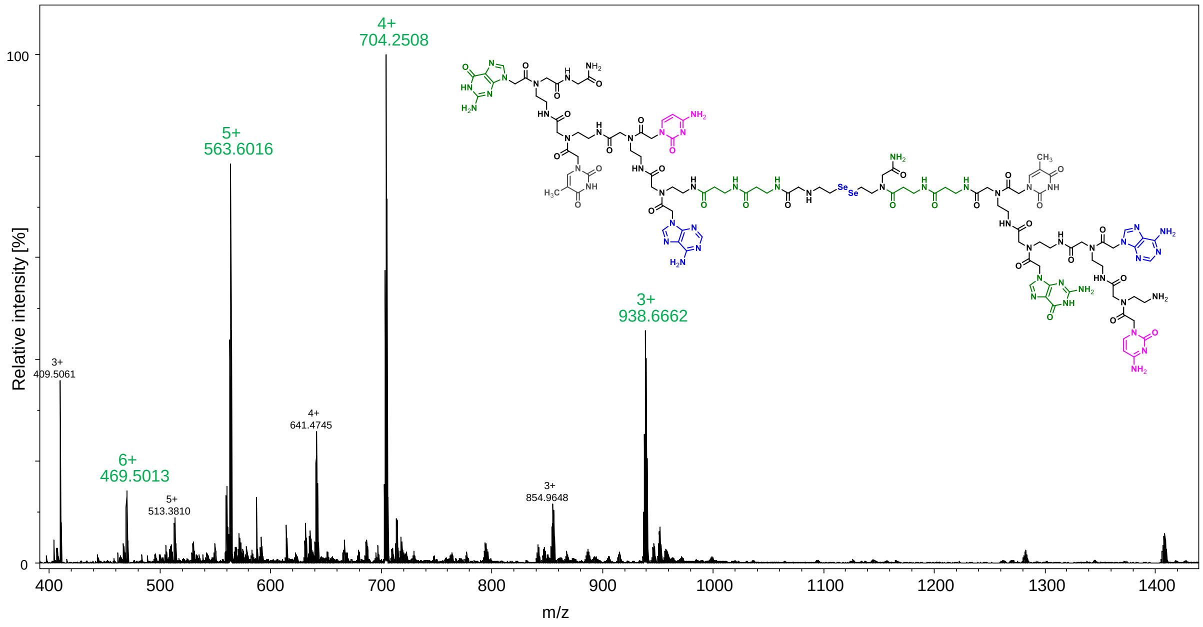


*Fig. S 32 ESI-MS spectrum obtained for PNA conjugate 3.2*

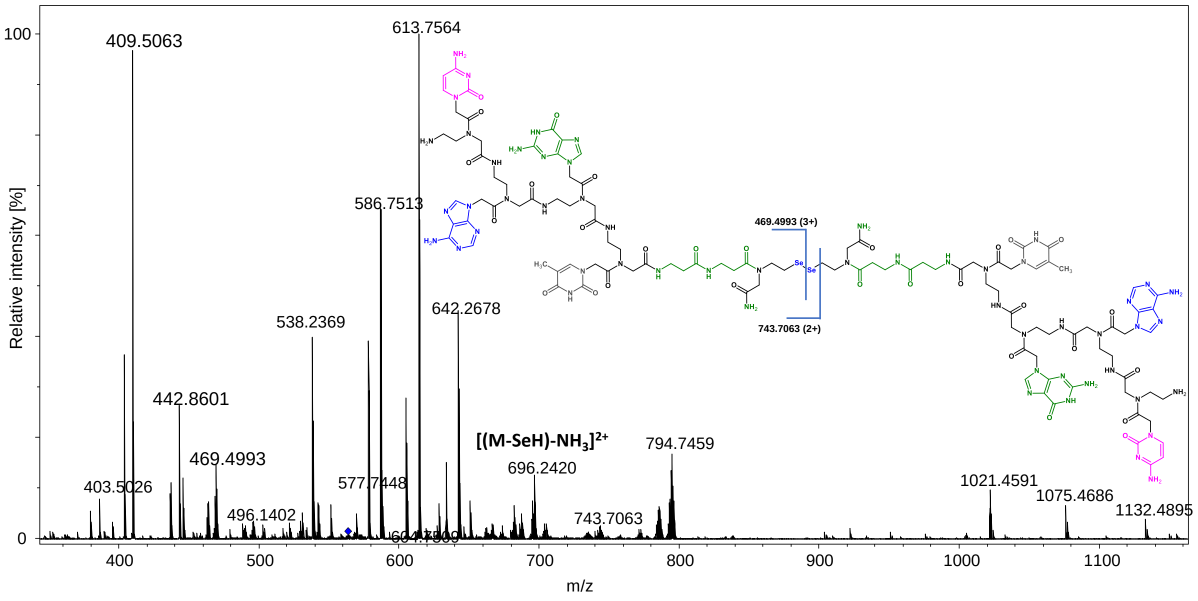


*Fig. S 33 ESI-MS/MS (CE 20 eV) spectrum obtained for PNA conjugate 3.2*

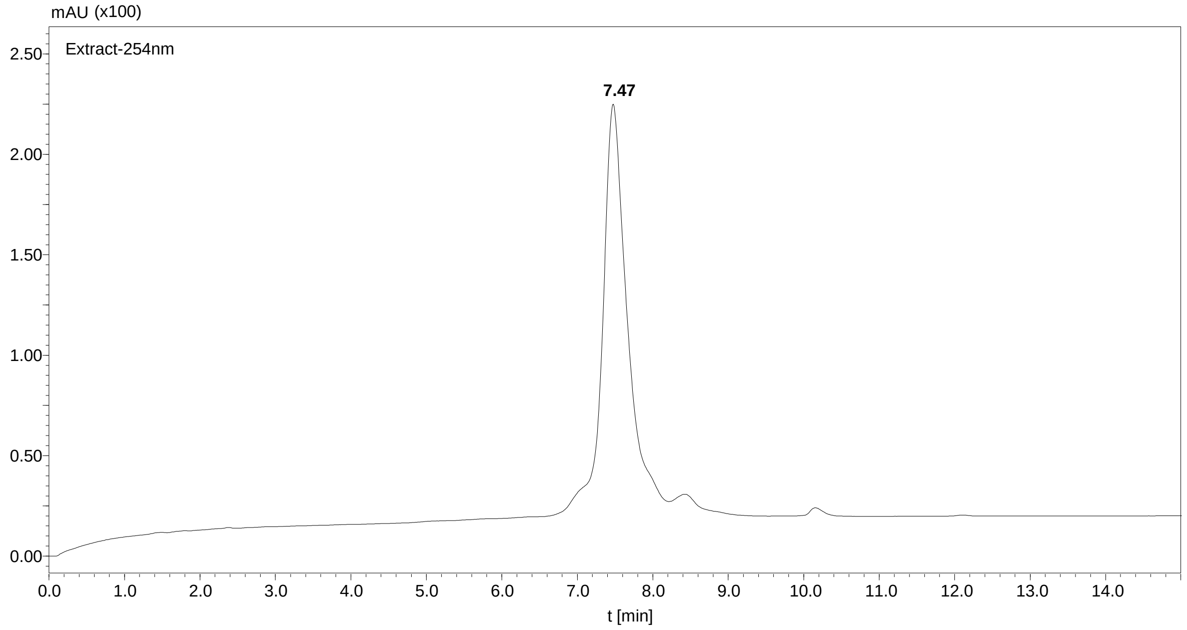


*Fig. S 34 HPLC chromatogram obtained for PNA conjugate 3.2*

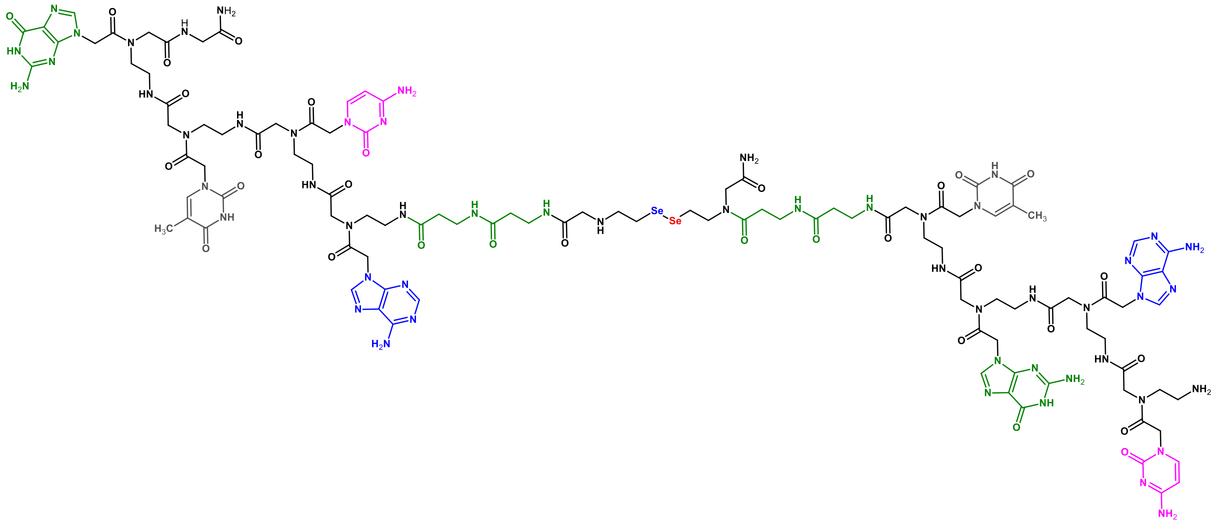

*Fig. S 35 Structure of metathesis product obtained  after visible-light irradiation of PNA conjugate 3.1 and 3.2*

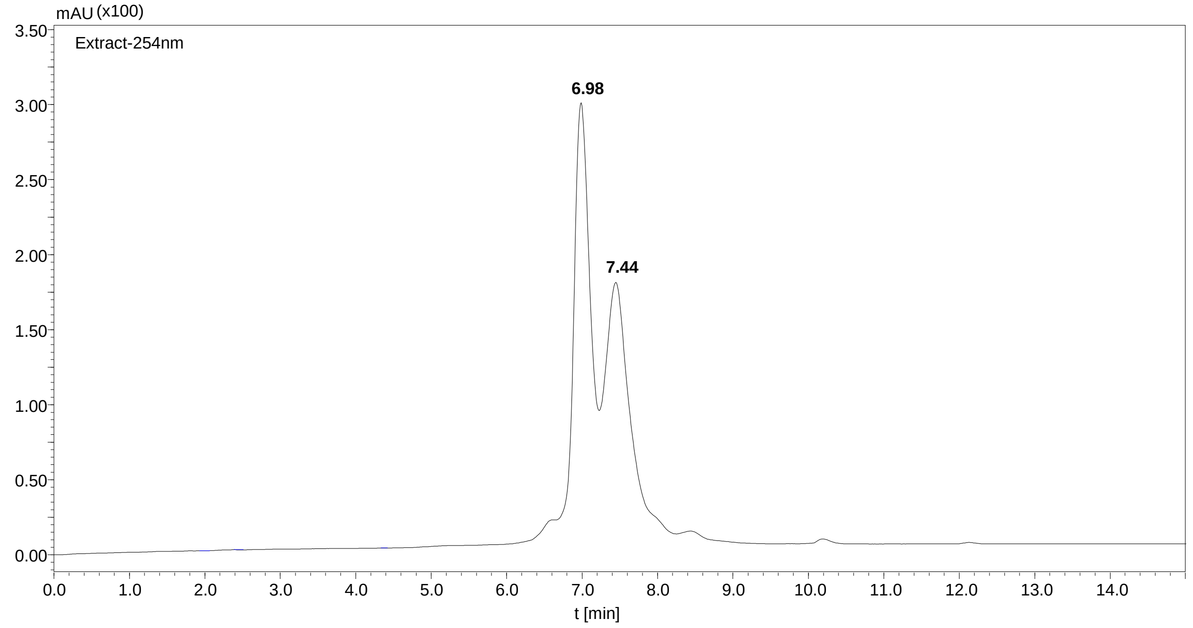


*Fig. S 36 HPLC chromatogram obtained for the mixture of PNA conjugate 3.1 and 3.2 before irradiation.*

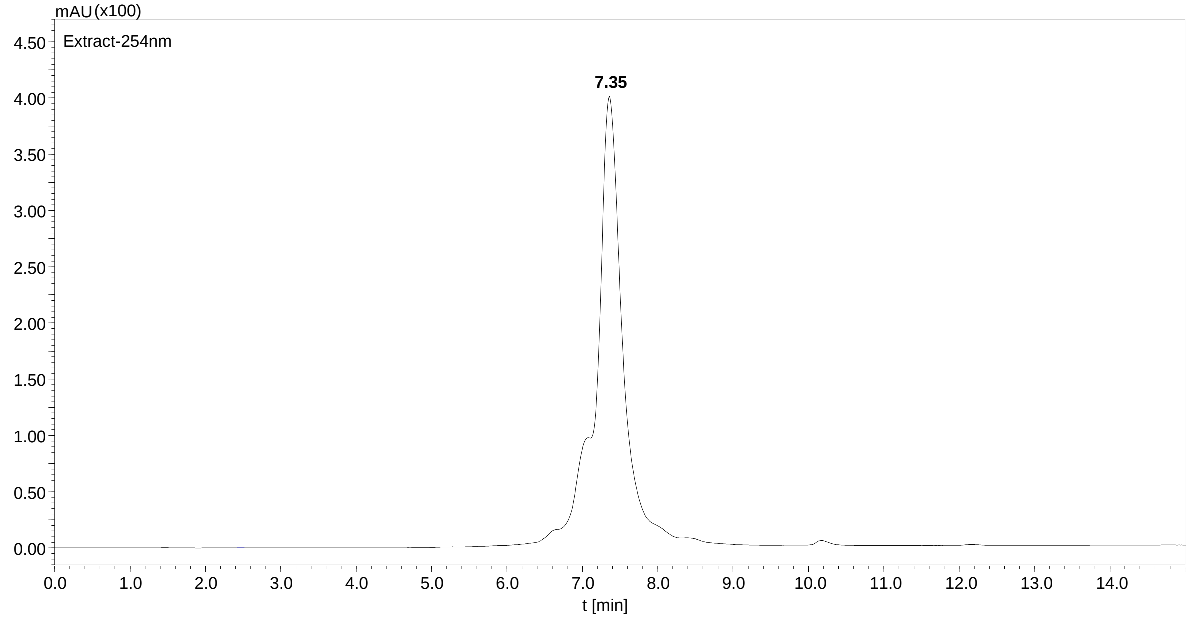


*Fig. S 37 Chromatogram HPLC obtained after visible-light irradiation of PNA conjugate 3.1 and 3.2.*

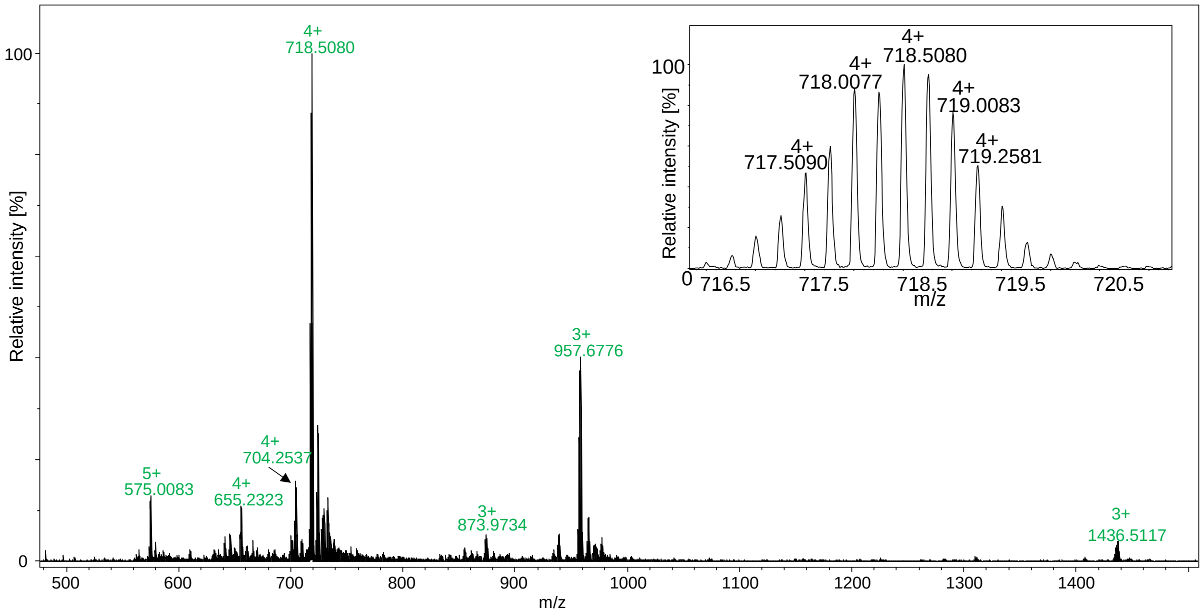


*Fig. S 38 ESI-MS spectrum obtained for the mixture after visible-light irradiation of PNA conjugate 3.1 and 3.2 (the most abundant peaks correspond to target compound)*

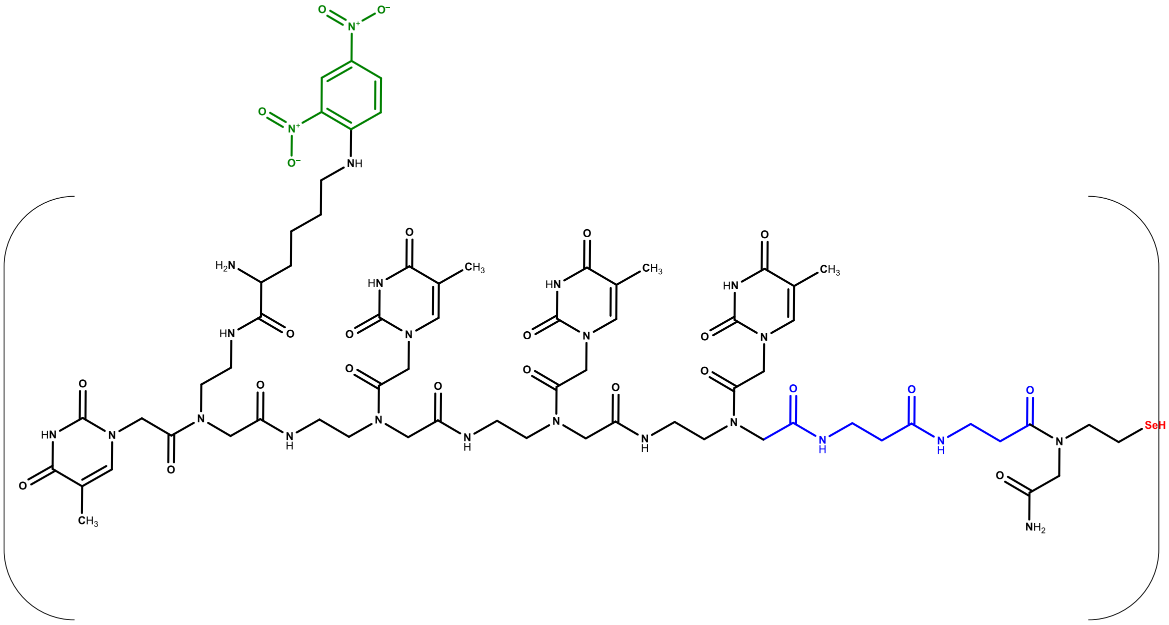

2

*Fig. S 39 Structure of PNA conjugate 4.1 containing 2,4-dinitrofluorophenyl chromophore*

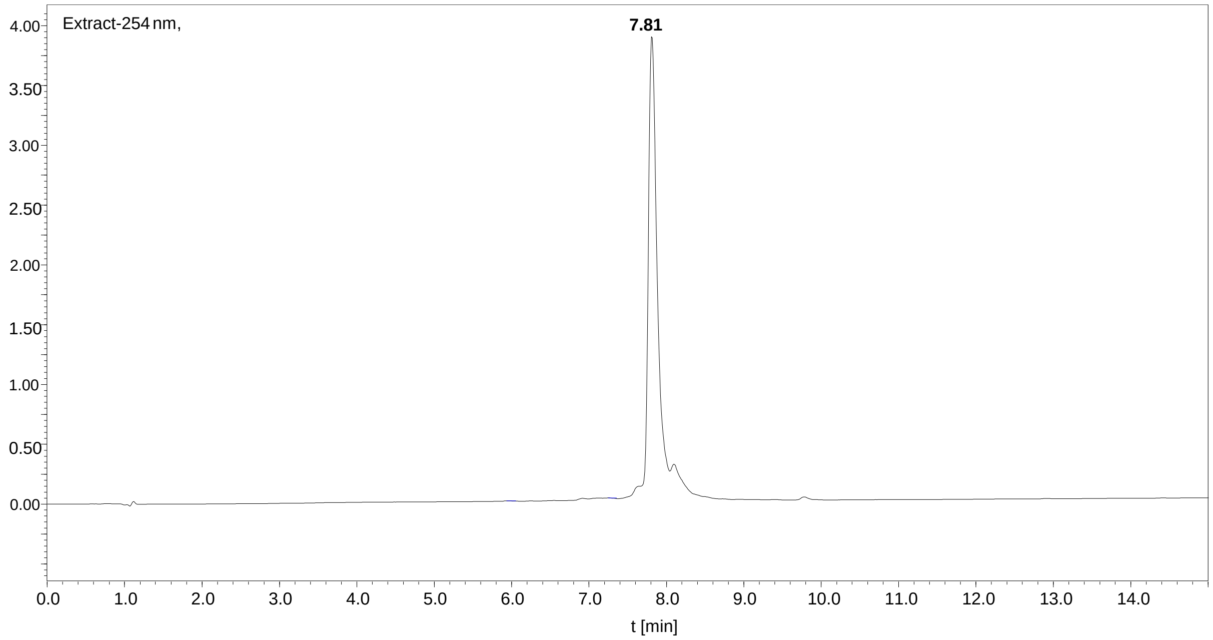


*Fig. S 40 HPLC chromatogram obtained for PNA conjugate 4.1*

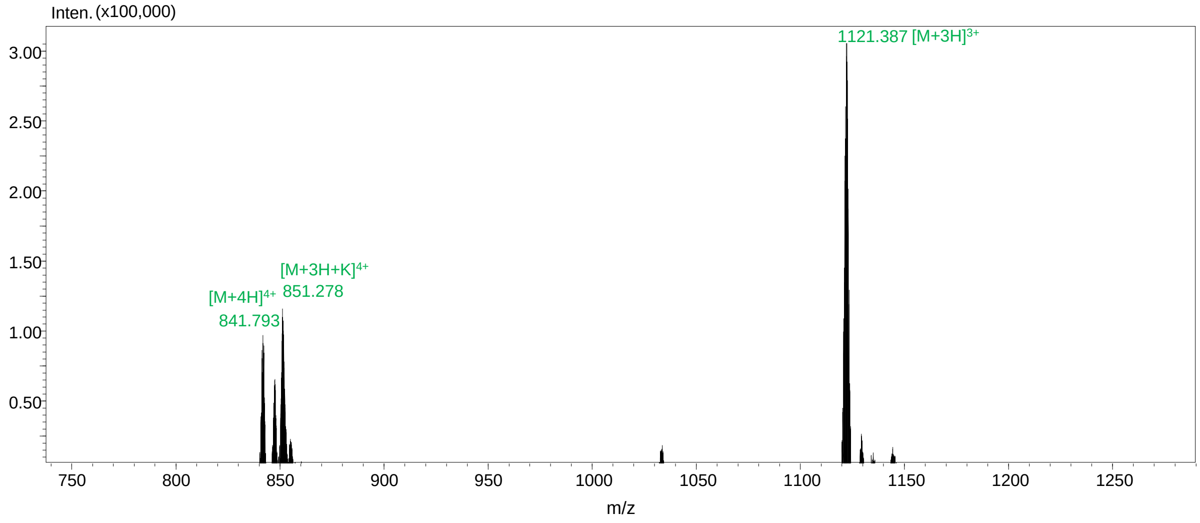


*Fig. S 41 ESI-MS spectrum obtained for PNA conjugate 4.1*

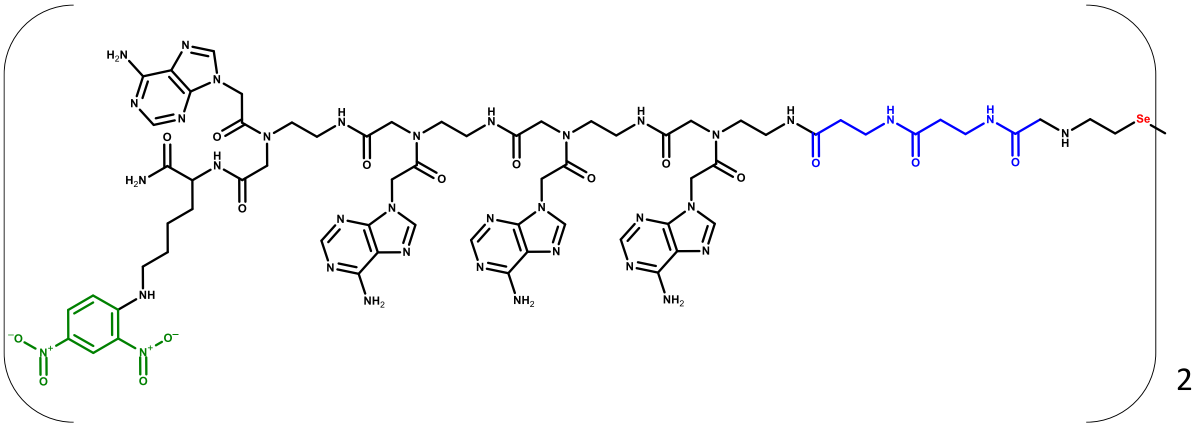

*Fig. S 42 Structure of PNA conjugate 4.2 containing 2,4-dinitrofluorophenyl chromophore*

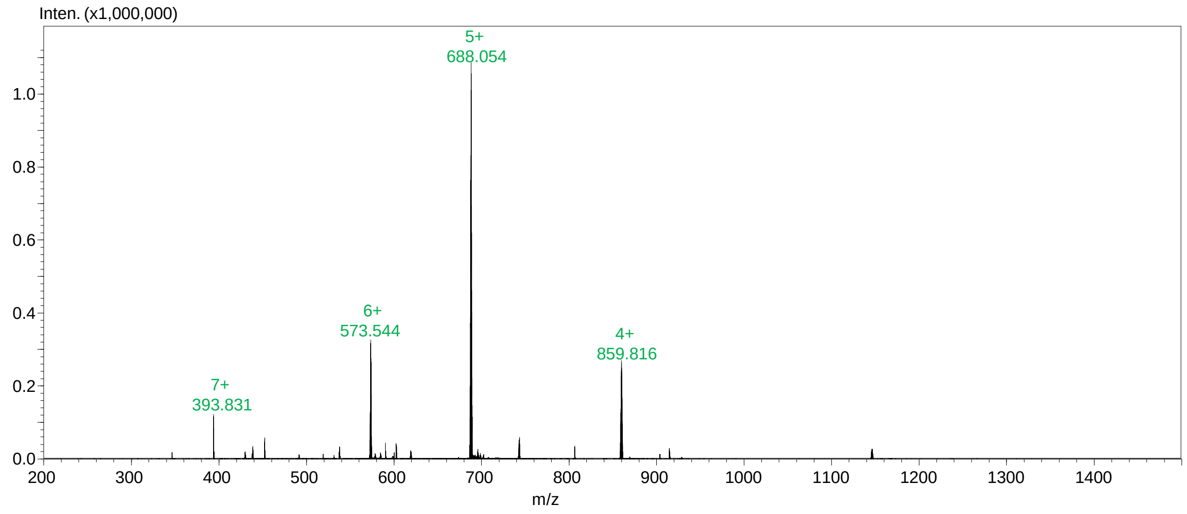


*Fig. S 43 ESI-MS spectrum obtained for PNA conjugate 4.2*

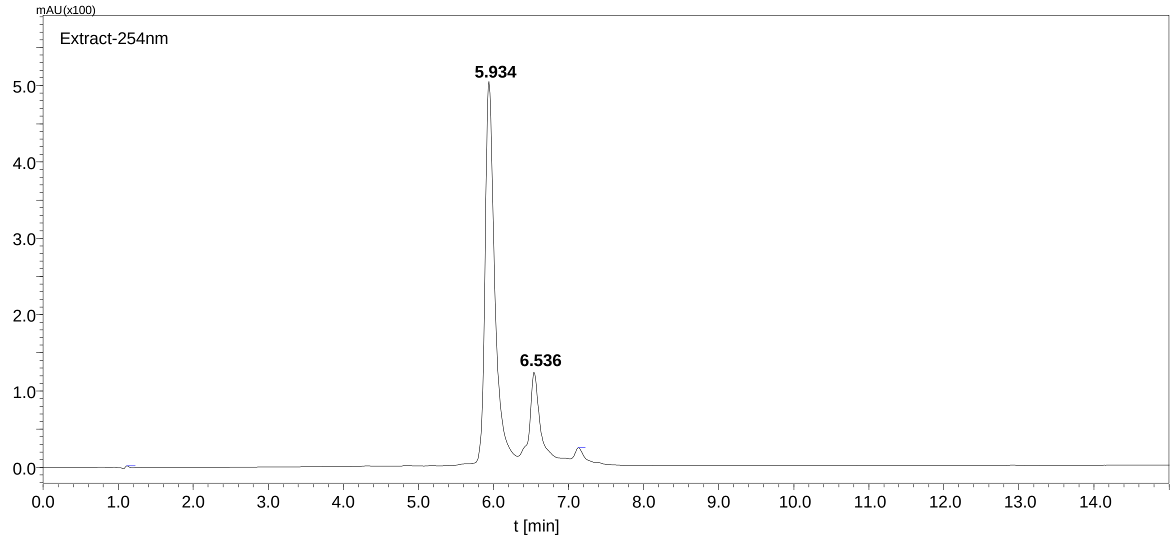


*Fig. S 44 HPLC chromatogram obtained for PNA conjugate 4.2*

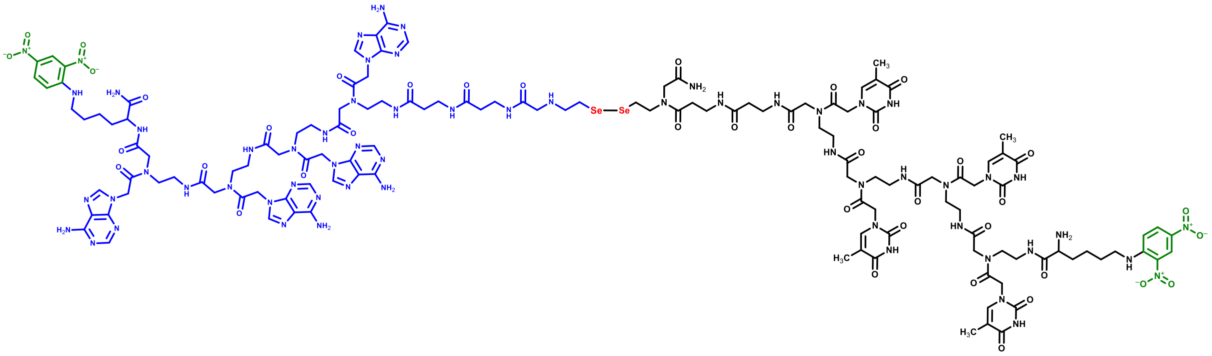

*Fig. S 45 Structure of the target product after visible-light irradiation of PNA conjugate 4.1 and 4.2*

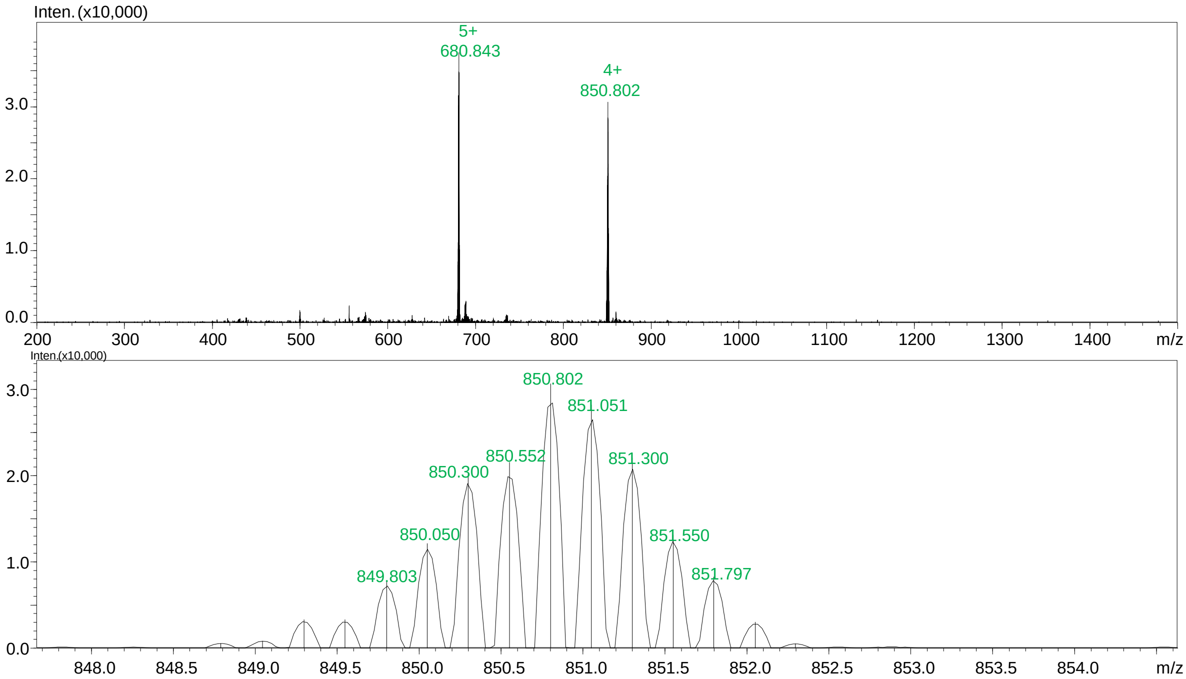


*Fig. S 46 ESI-MS spectrum obtained for target the product after visible-light irradiation of PNA conjugate 4.1 and 4.2*

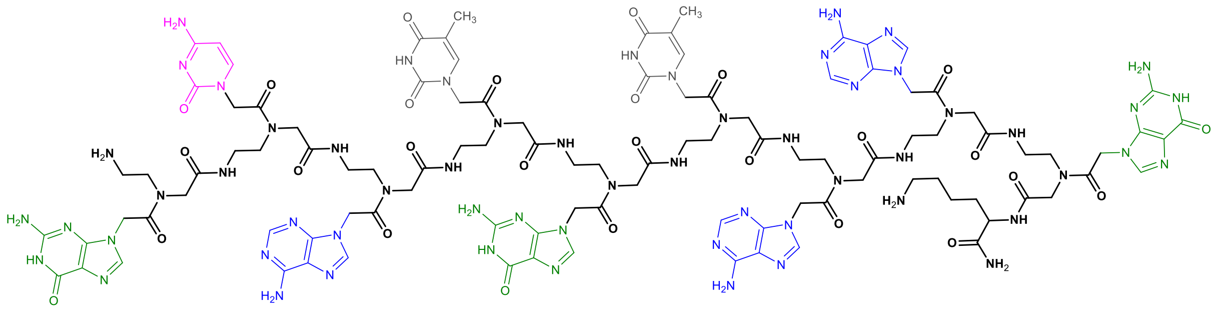

*Fig. S 47 Structure of PNA template T1*

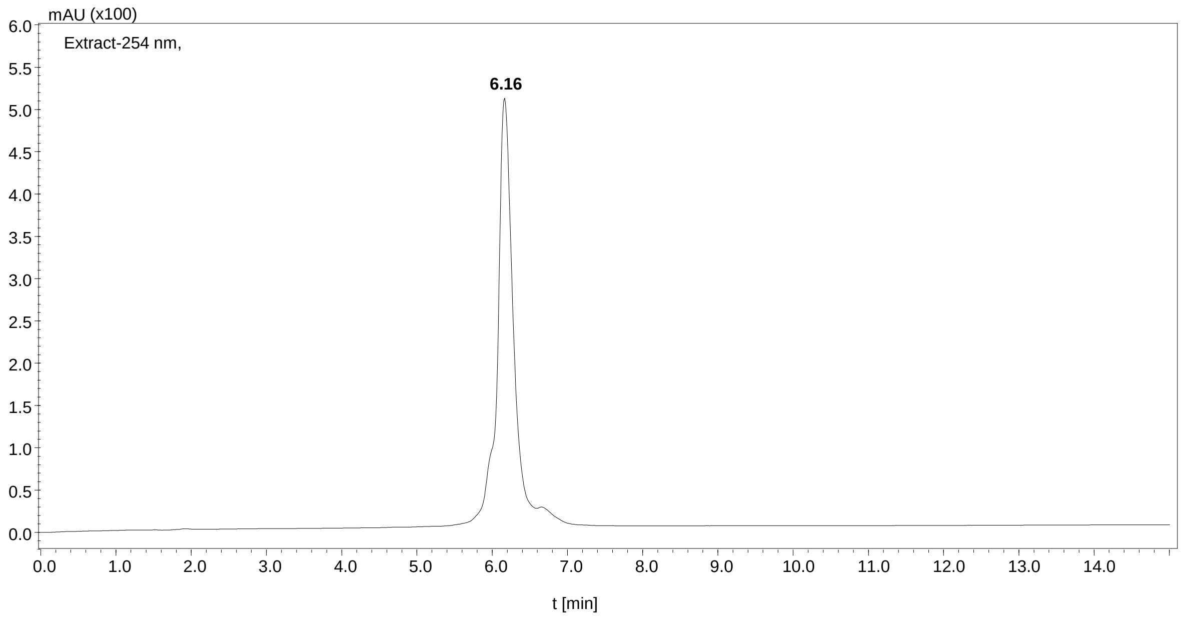


*Fig. S 48 HPLC chromatogram obtained for PNA template T1*

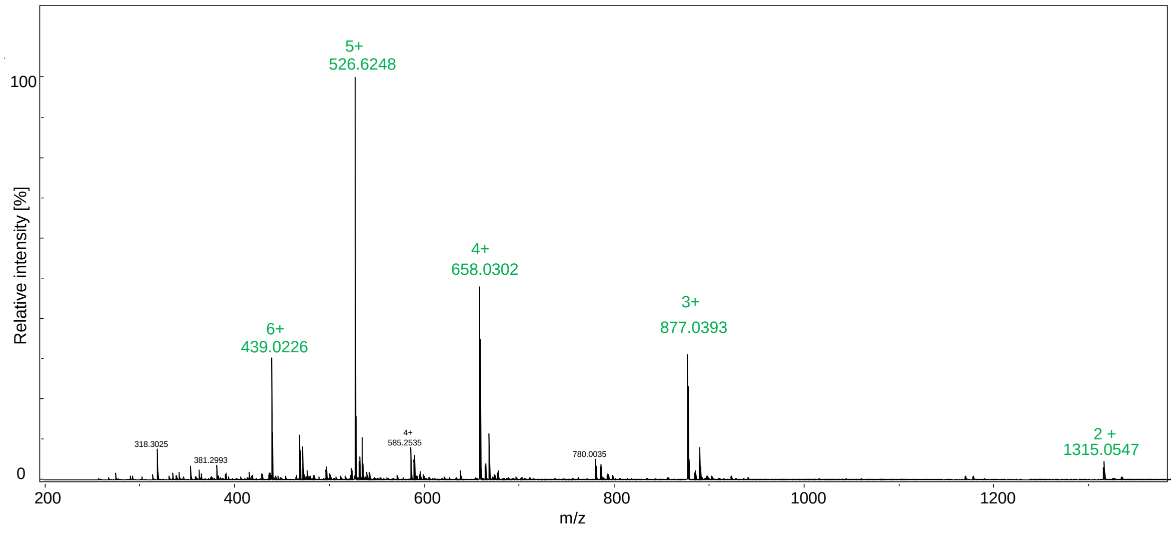


*Fig. S 49 ESI-MS spectrum obtained for PNA template T1*

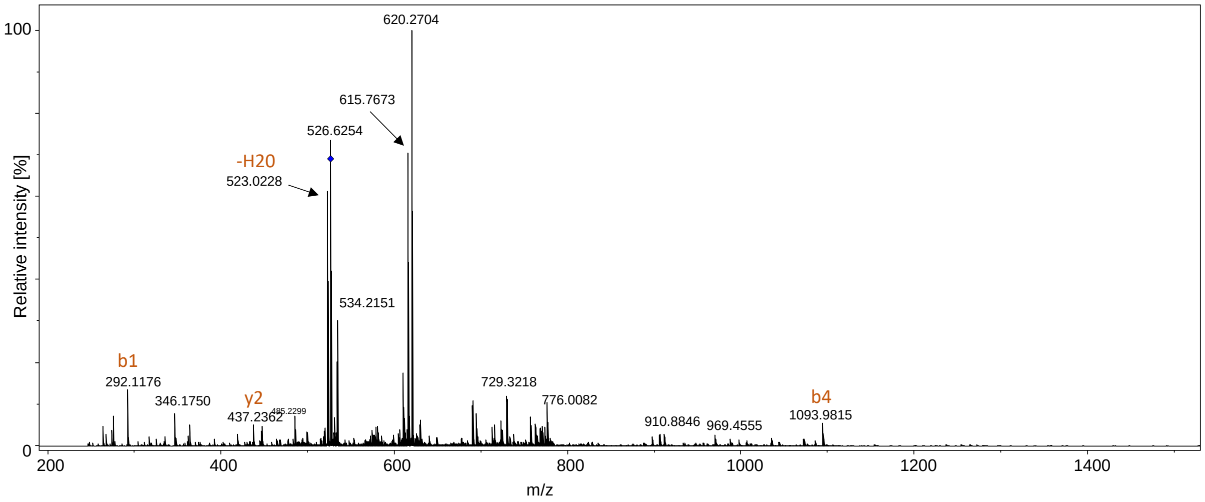


*Fig. S 50 ESI-MS/MS (CE 25 eV) spectrum obtained for PNA template T1*

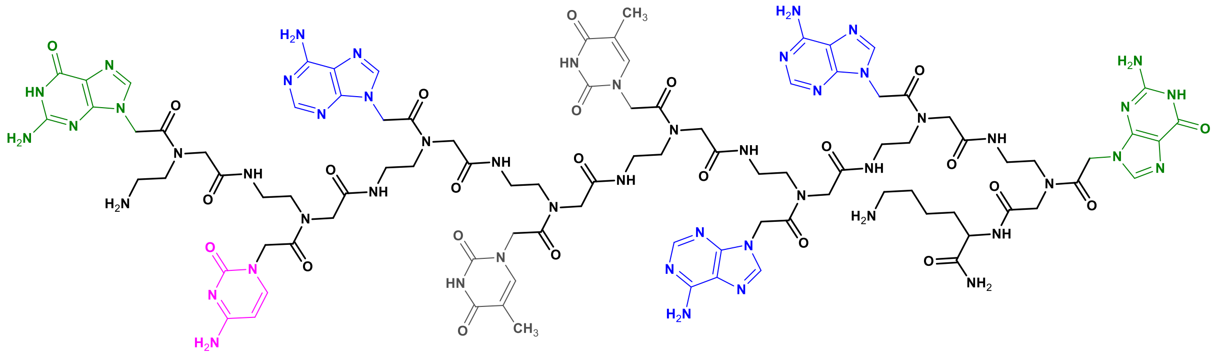

*Fig. S 51 Structure of PNA template T2*

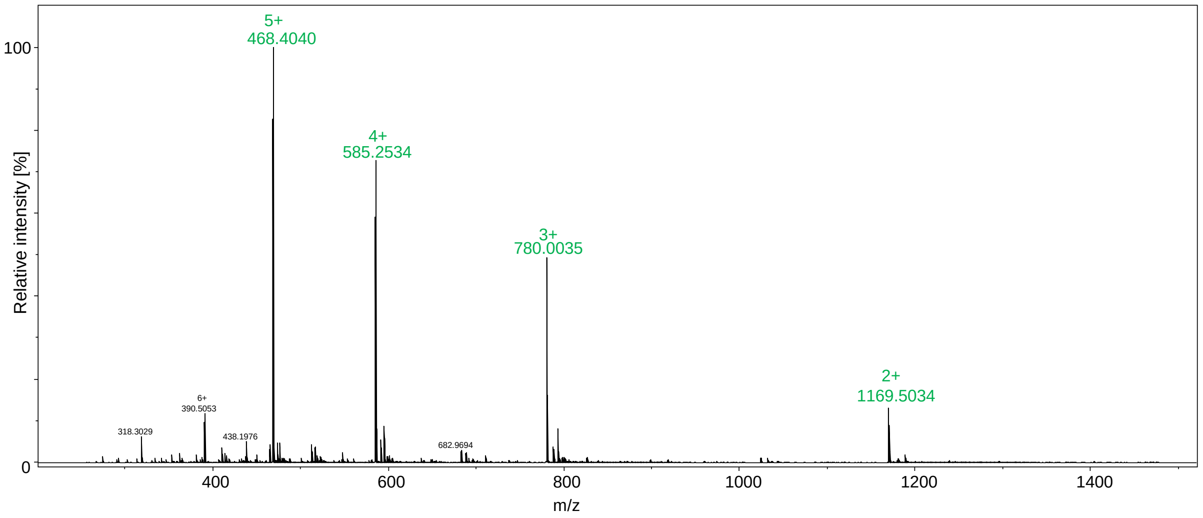


*Fig. S 52 ESI-MS spectrum obtained for PNA template T2*

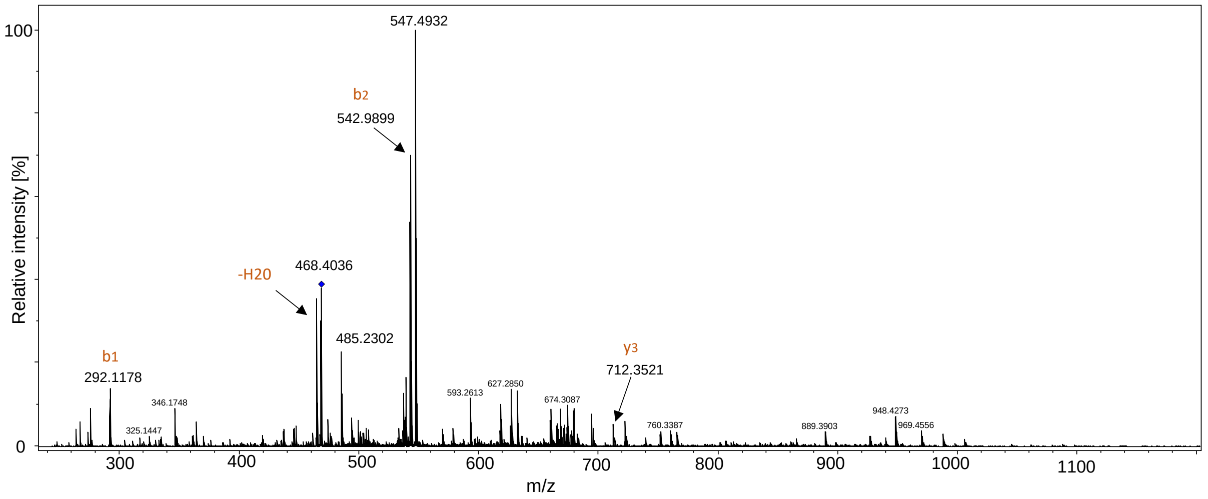


*Fig. S 53 ESI-MS/MS (CE 25 eV) spectrum obtained for PNA template T2*

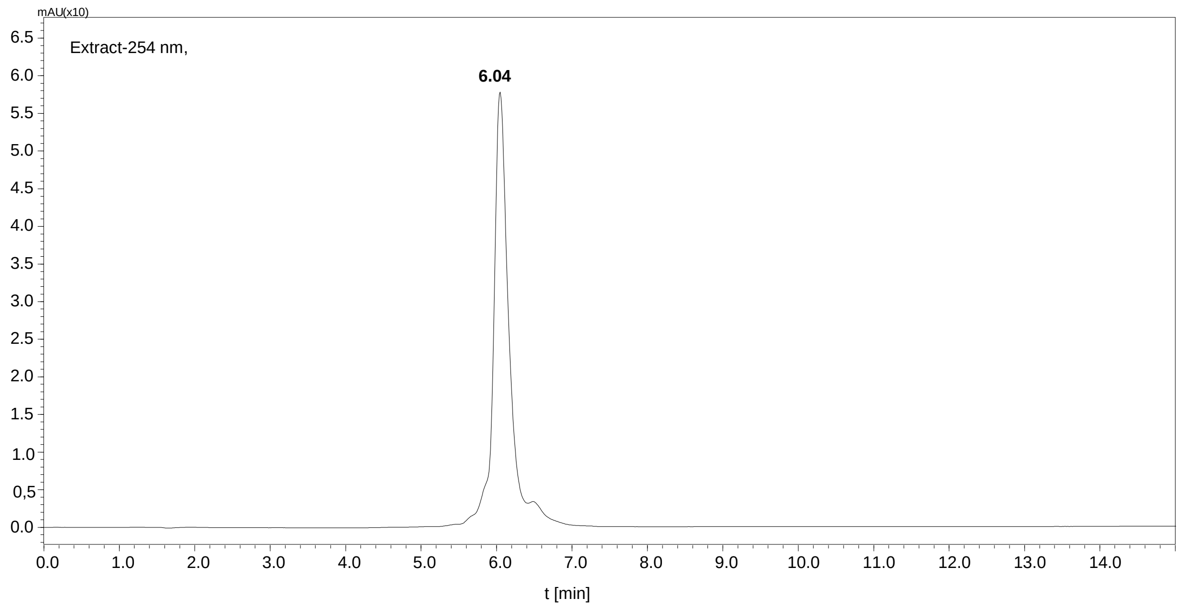


*Fig. S 54 HPLC chromatogram obtained for PNA template T2*

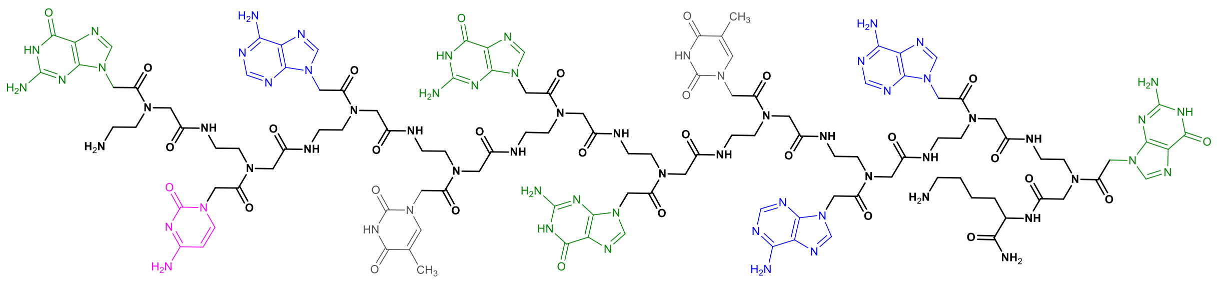

*Fig. S 55 Structure of PNA template T3*

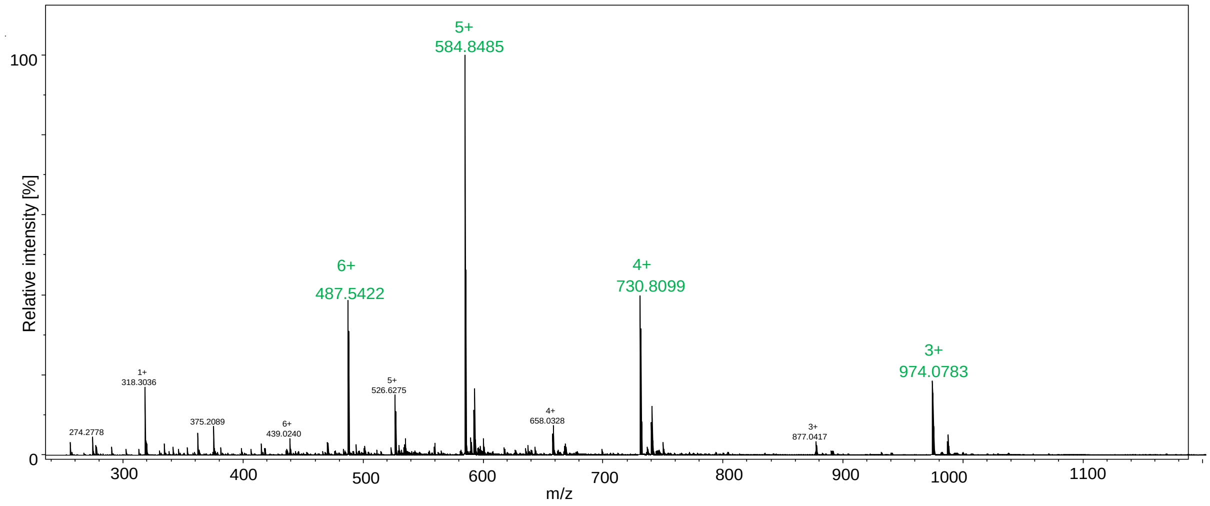


*Fig. S 56 ESI-MS spectrum obtained for PNA template T3*

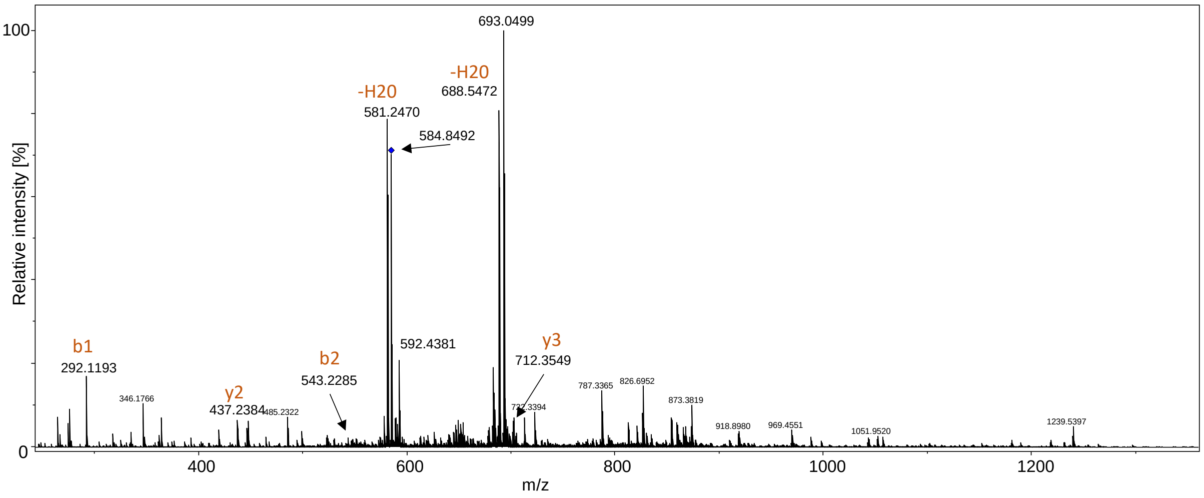


*Fig. S 57 ESI-MS/MS (CE 25 eV) spectrum obtained for PNA template T3*

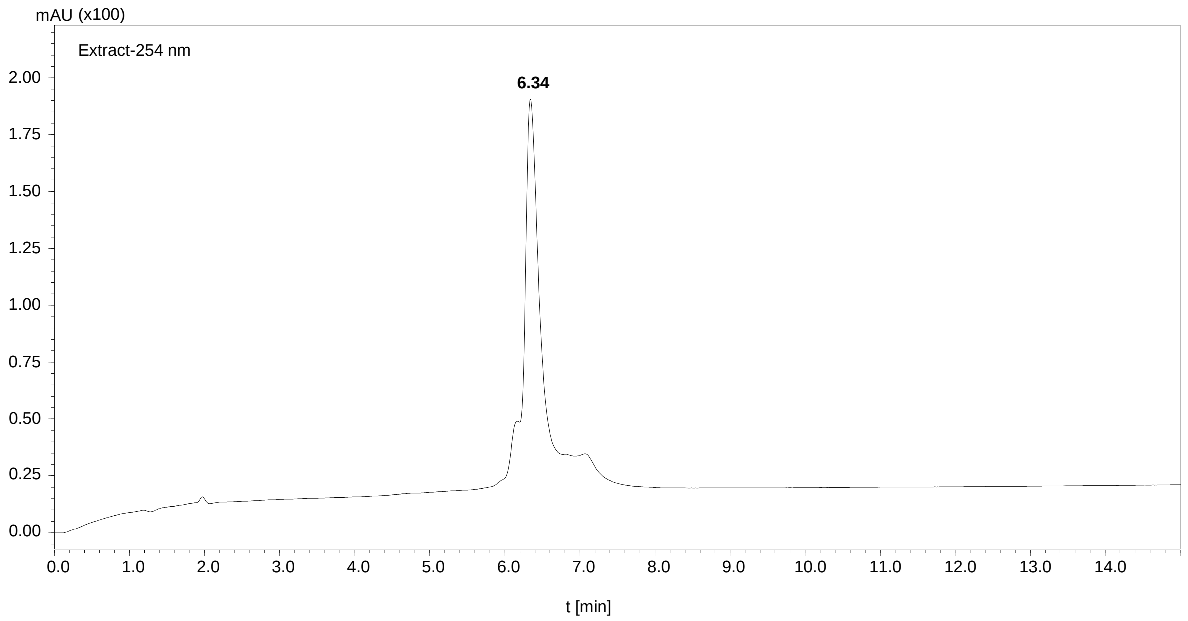


*Fig. S 58 HPLC chromatogram obtained for PNA template T3*

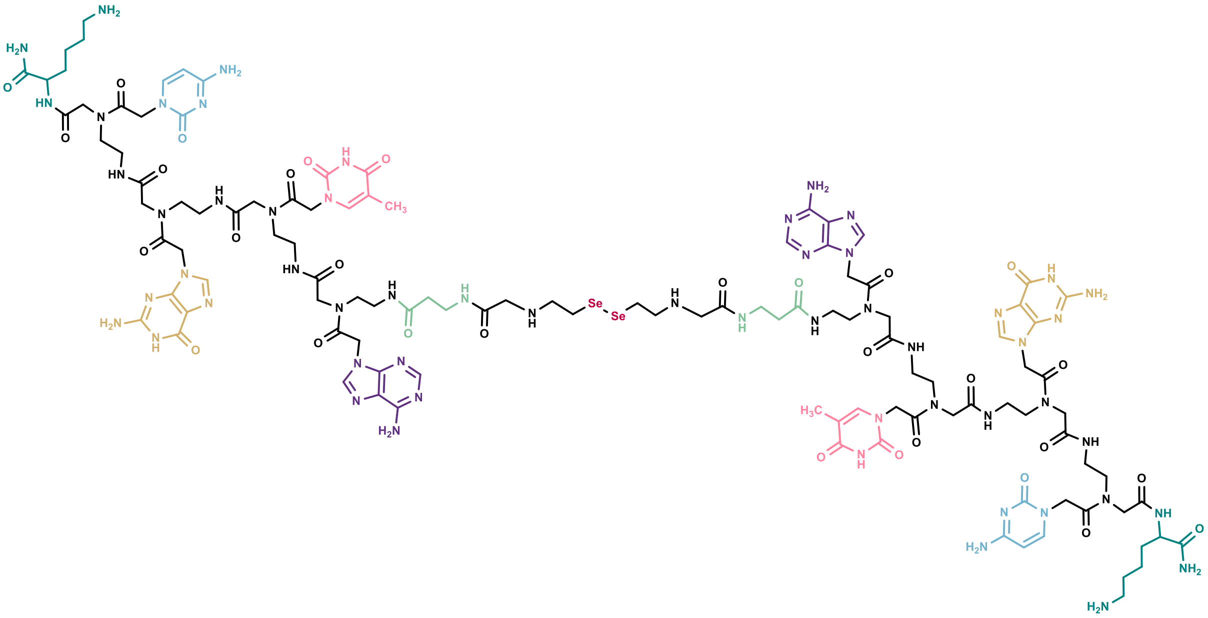

*Fig. S 59 Structure of PNA conjugate 5.1*

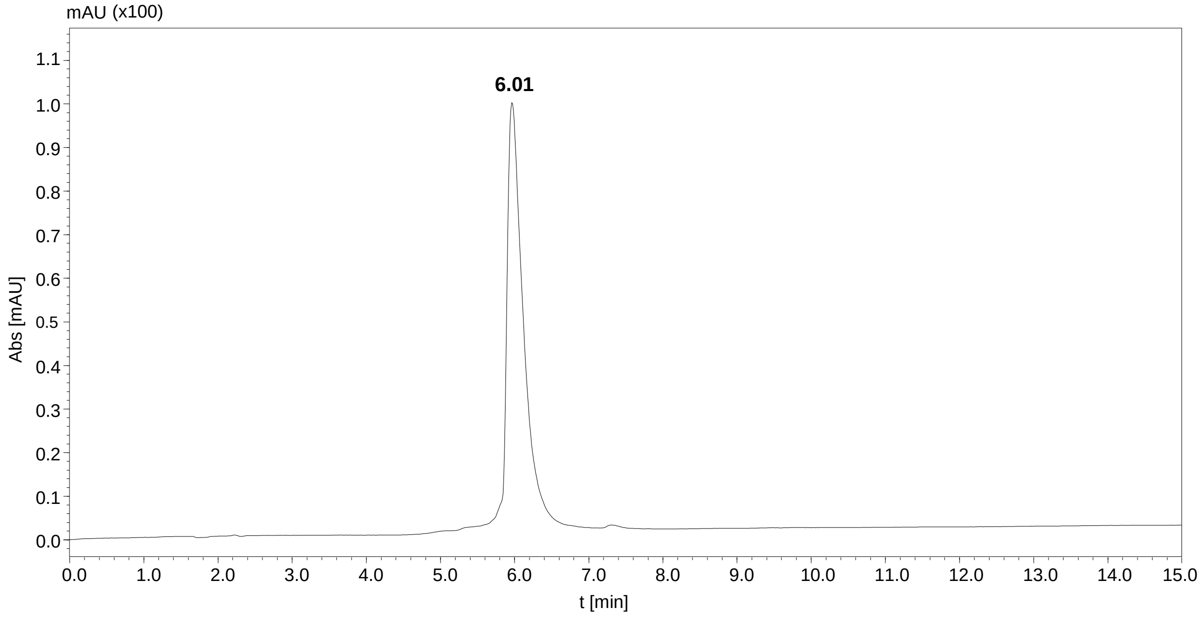


*Fig. S 60 HPLC chromatogram obtained for PNA conjugate 5.1*

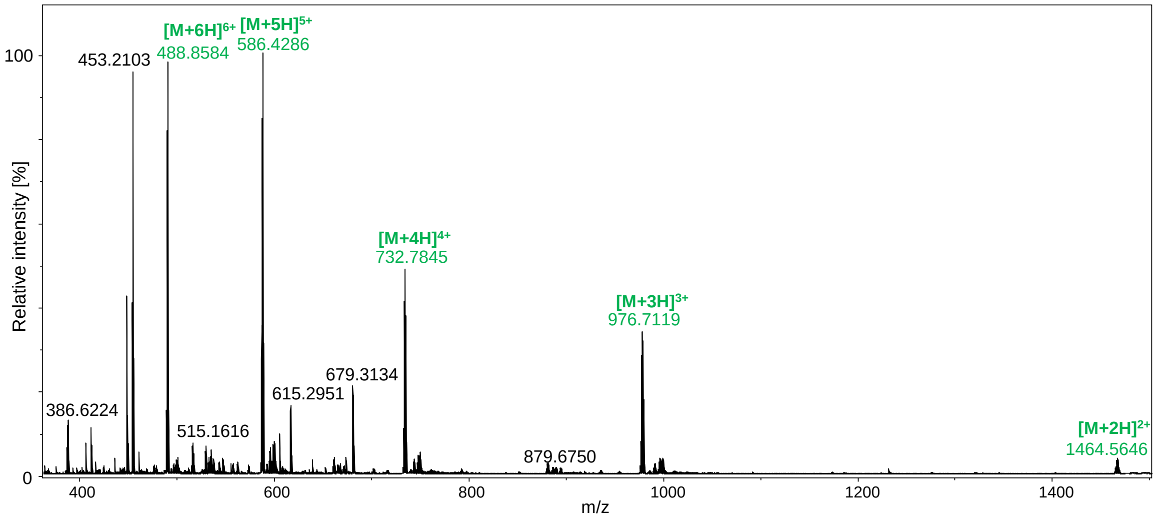


*Fig. S 61 ESI-MS spectrum obtained for PNA conjugate 5.1*

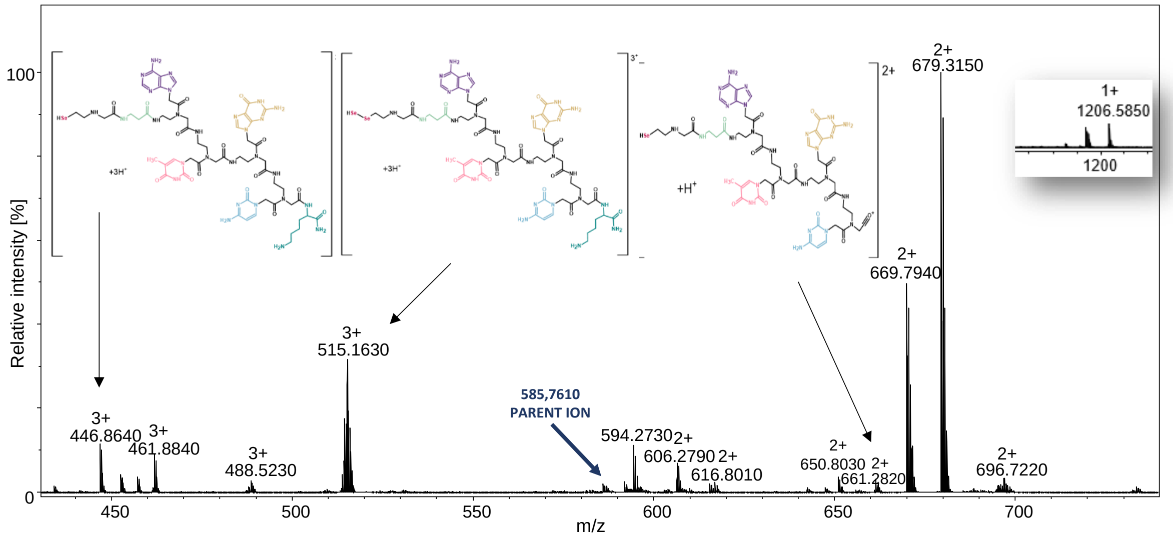


*Fig. S 62 ESI-MS/MS (CE 22eV) spectrum obtained for PNA conjugate 5.1*

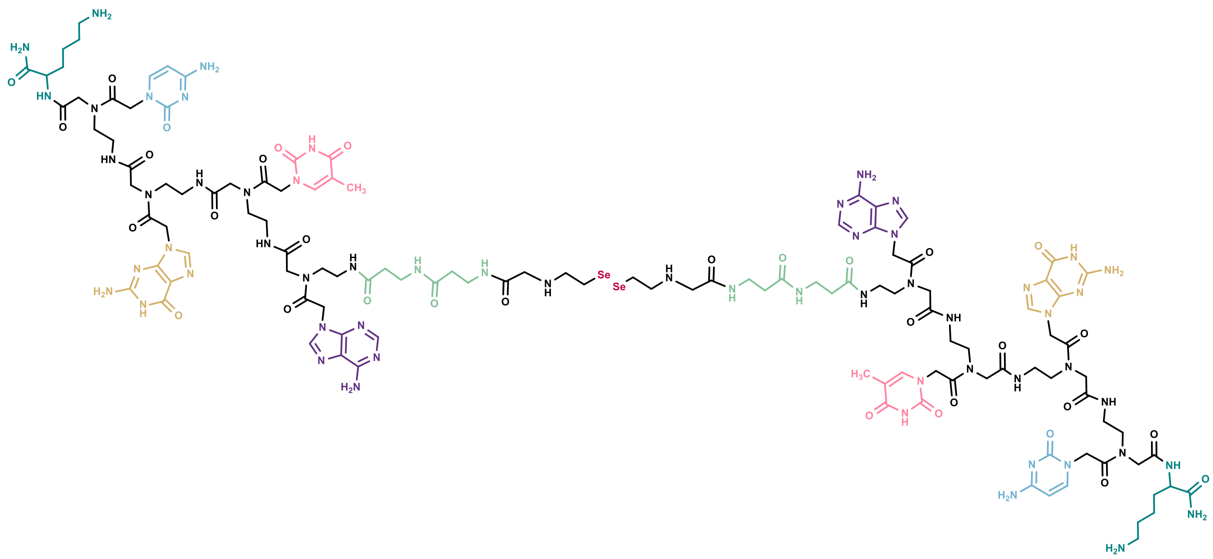

*Fig. S 63 Structure of PNA conjugate 6.1*

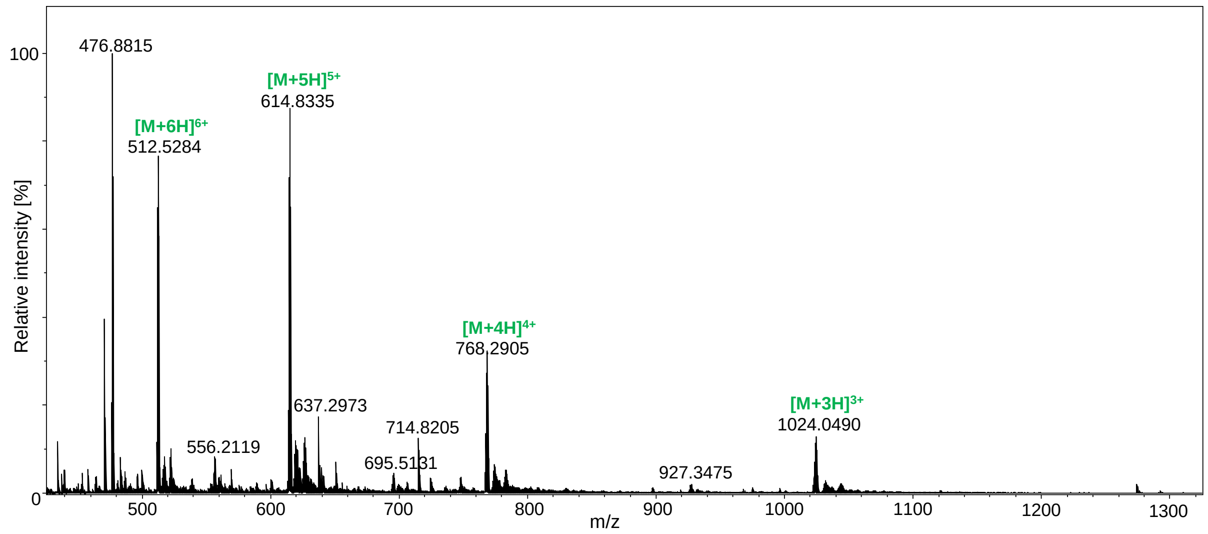


*Fig. S 64 ESI-MS spectrum obtained for PNA conjugate 6.1*

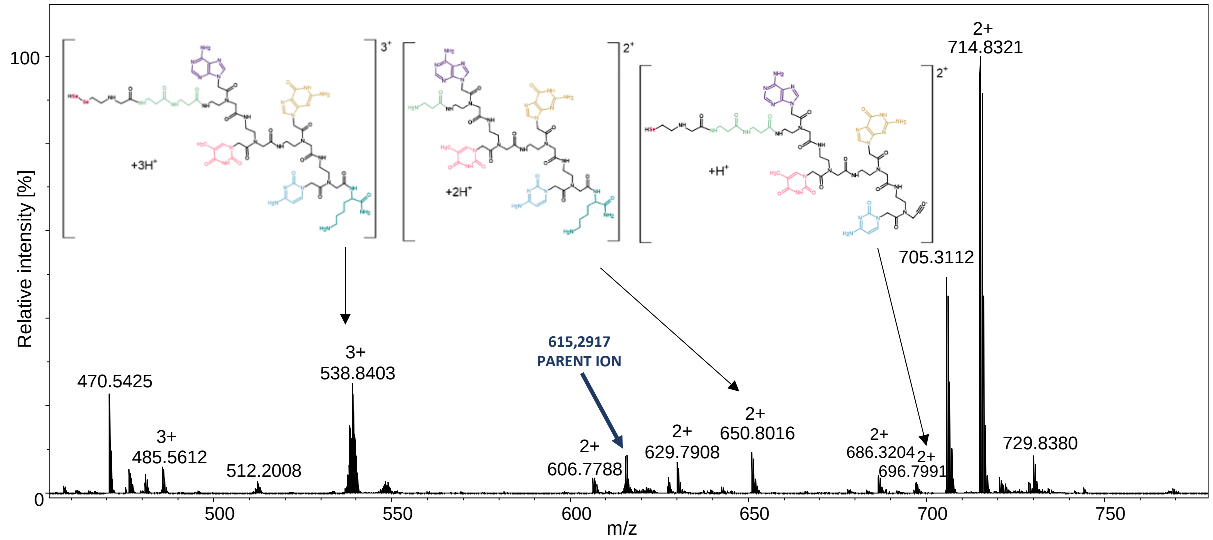


*Fig. S 65 ESI-MS/MS (CE 25 eV) spectrum obtained for PNA conjugate 6.1*

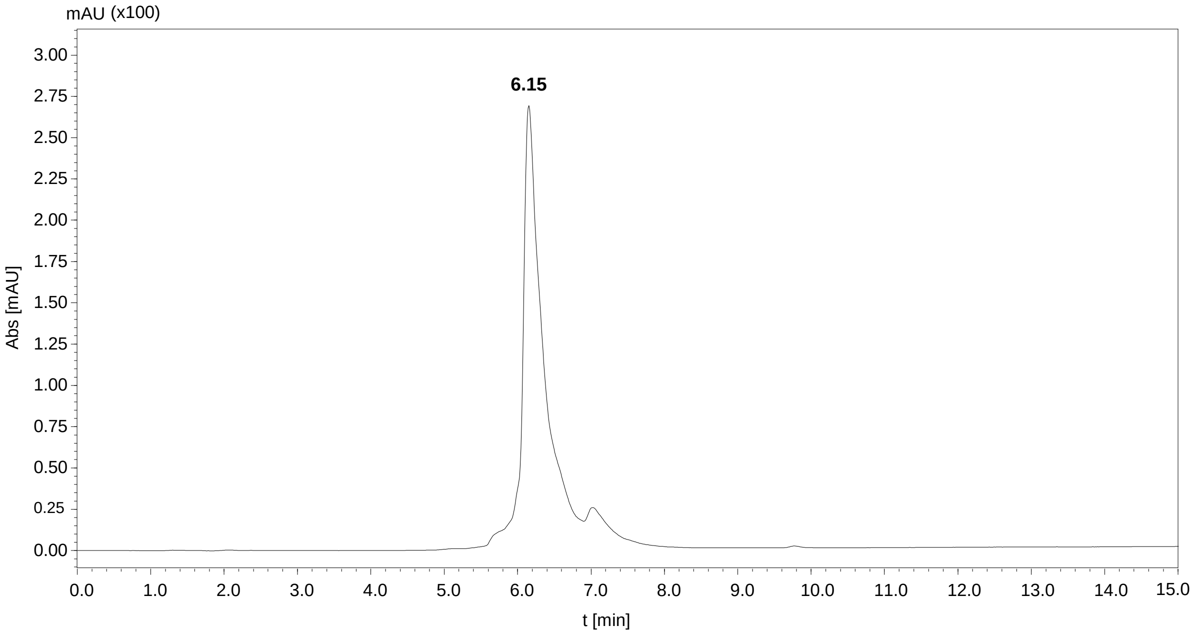


*Fig. S 66 HPLC chromatogram obtained for PNA conjugate 6.1*

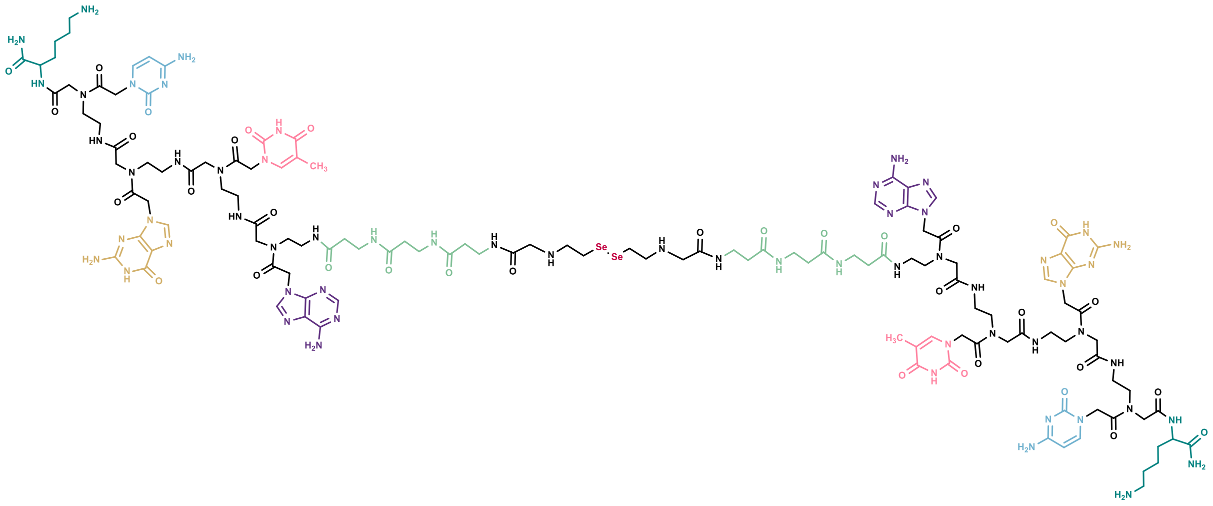

*Fig. S 67 Structure of PNA conjugate 7.1*

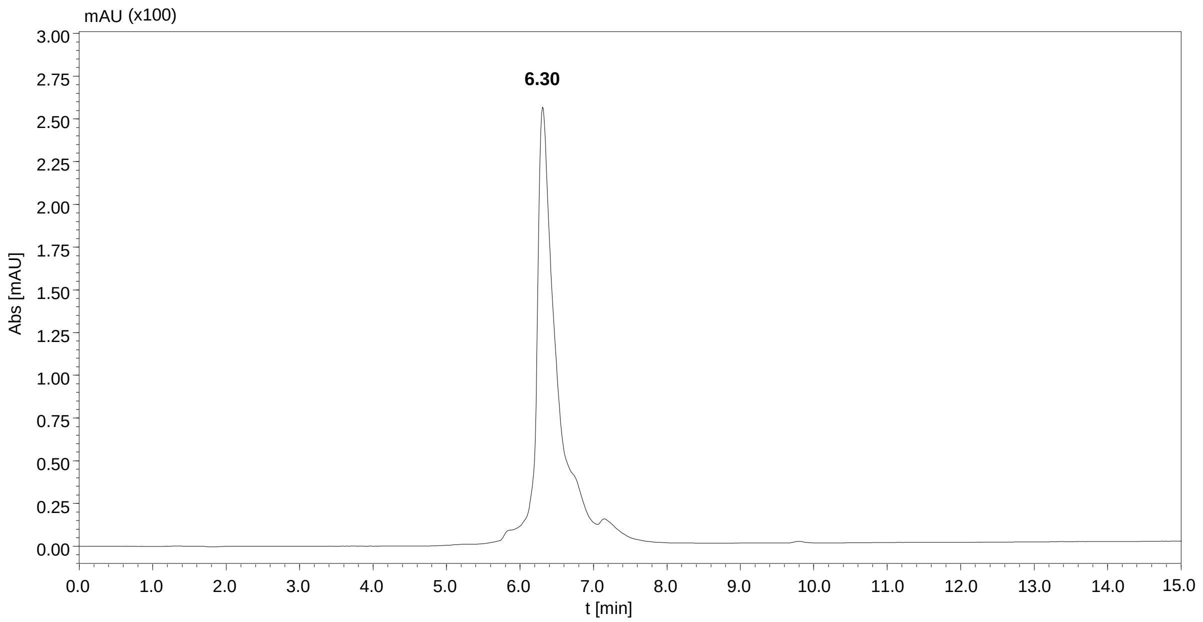


*Fig. S 68 HPLC chromatogram spectrum obtained for PNA conjugate 7.1*

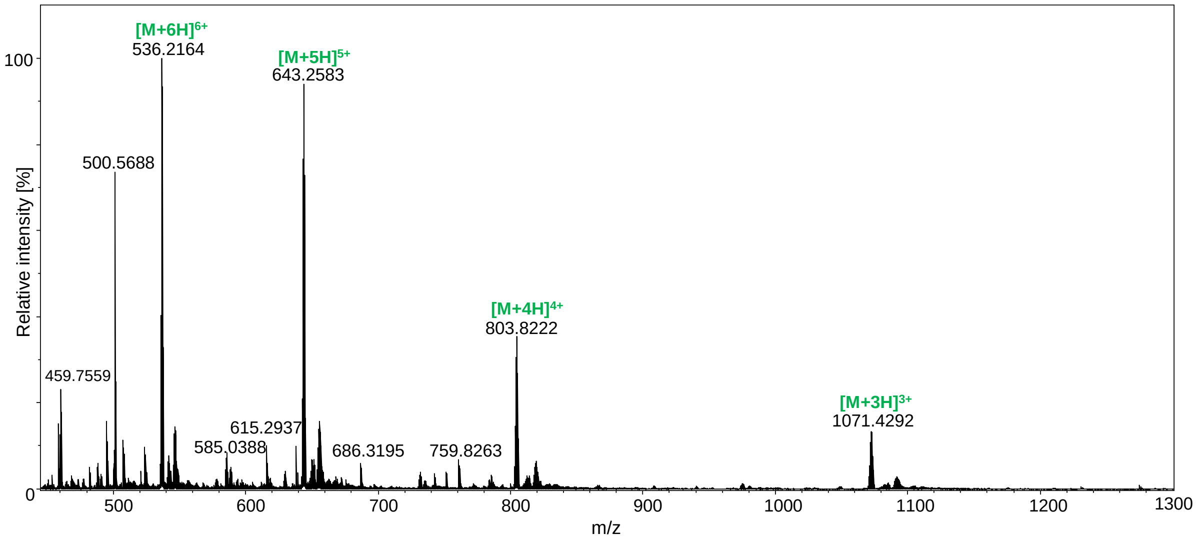


*Fig. S 69 ESI-MS spectrum obtained for PNA conjugate 7.1*

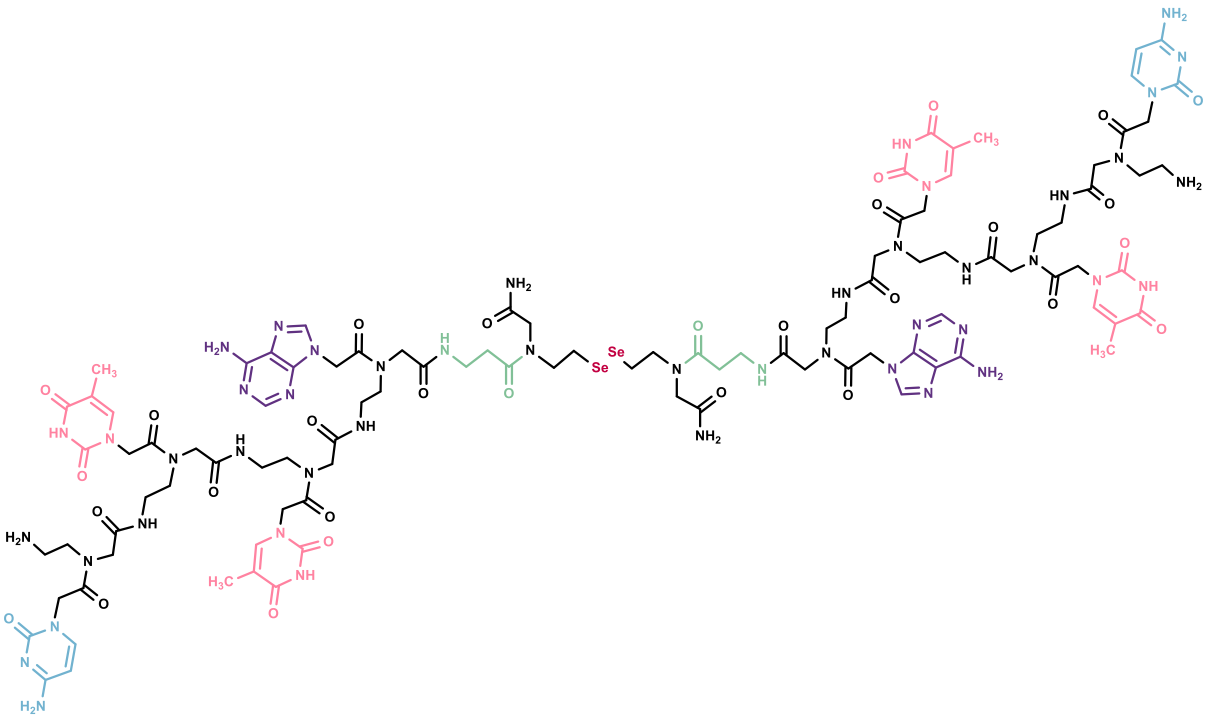

*Fig. S 70 Structure of PNA conjugate 5.2*

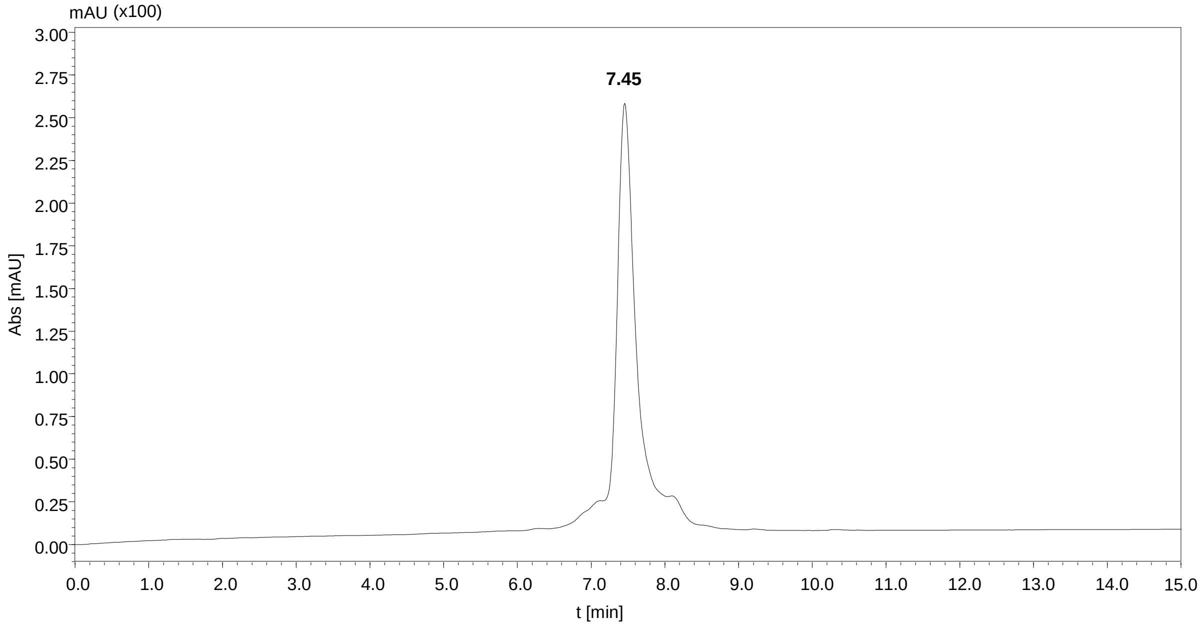


*Fig. S 71 HPLC chromatogram obtained for PNA conjugate 5.2*

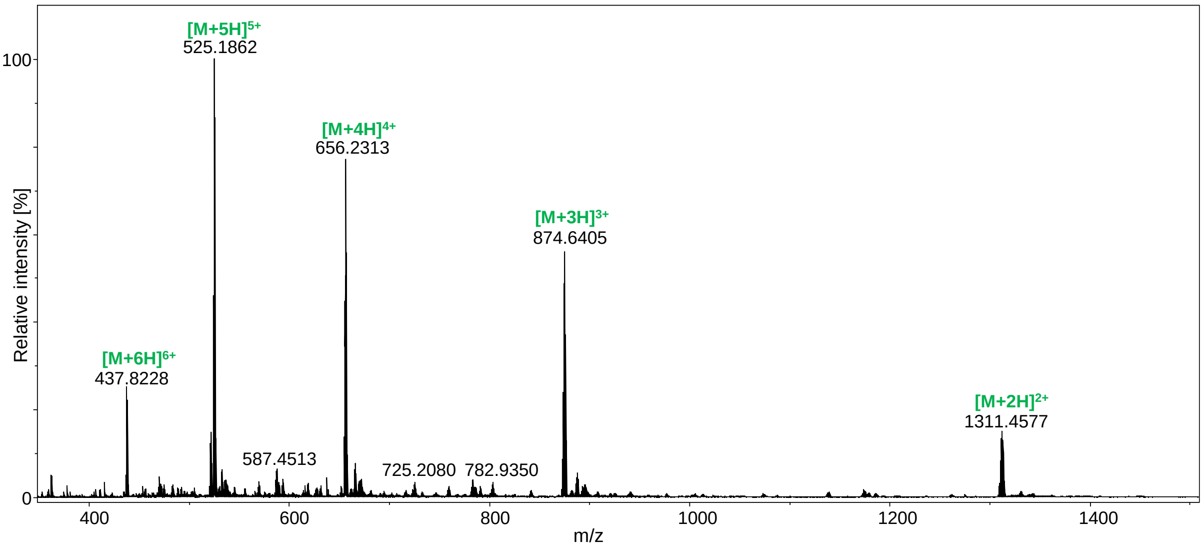


*Fig. S 72 ESI-MS spectrum obtained for PNA conjugate 5.2*

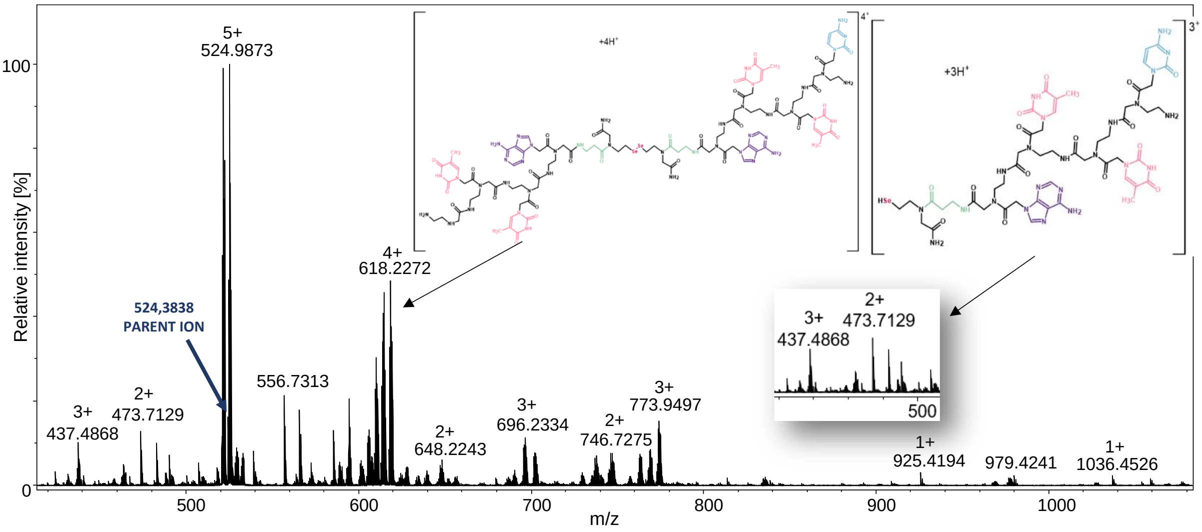


*Fig. S 73 ESI-MS/MS (CE 25 eV) spectrum obtained for PNA conjugate 5.2*

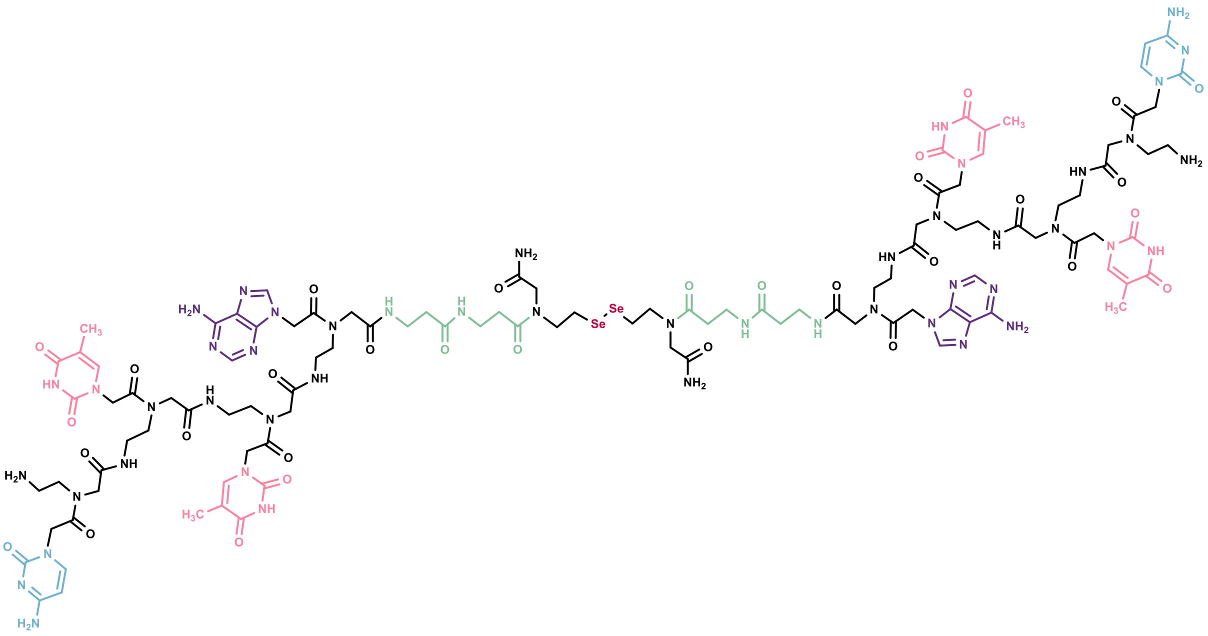

*Fig. S 74 Structure of PNA conjugate 6.2*

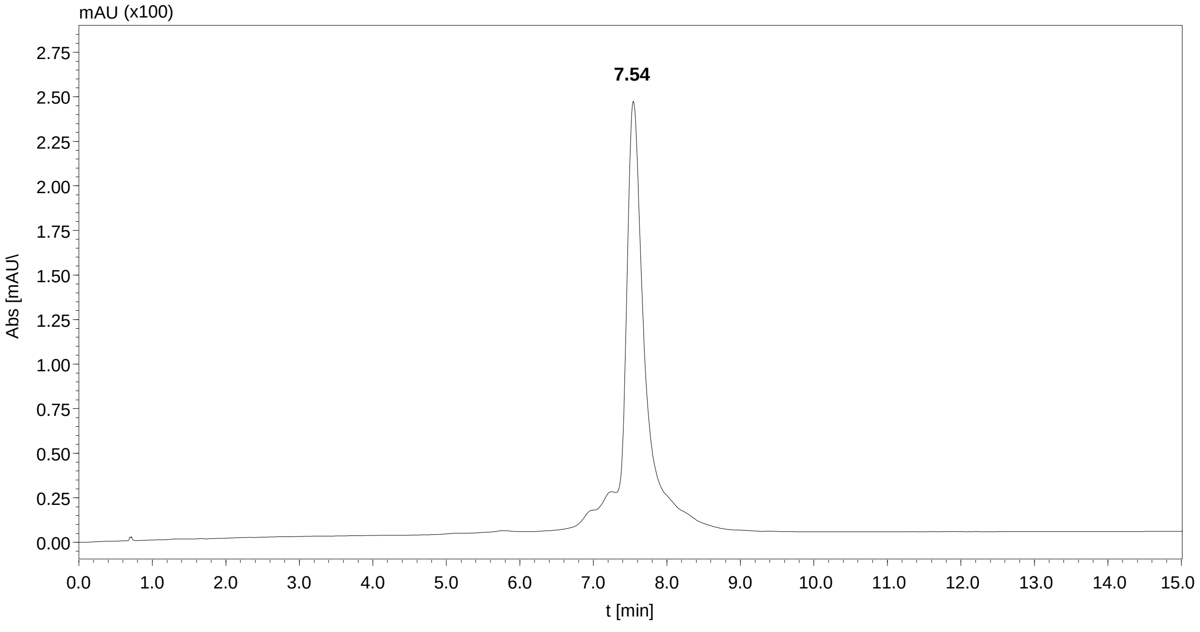


*Fig. S 75 HPLC chromatogram obtained for PNA conjugate 6.2*

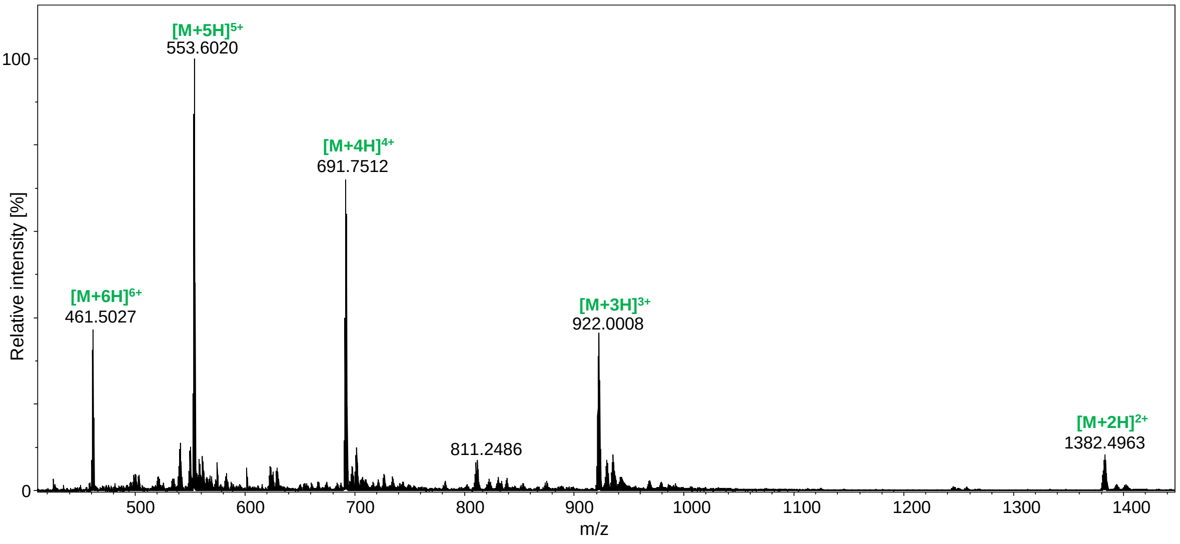


*Fig. S 76 ESI-MS spectrum obtained for PNA conjugate 6.2*

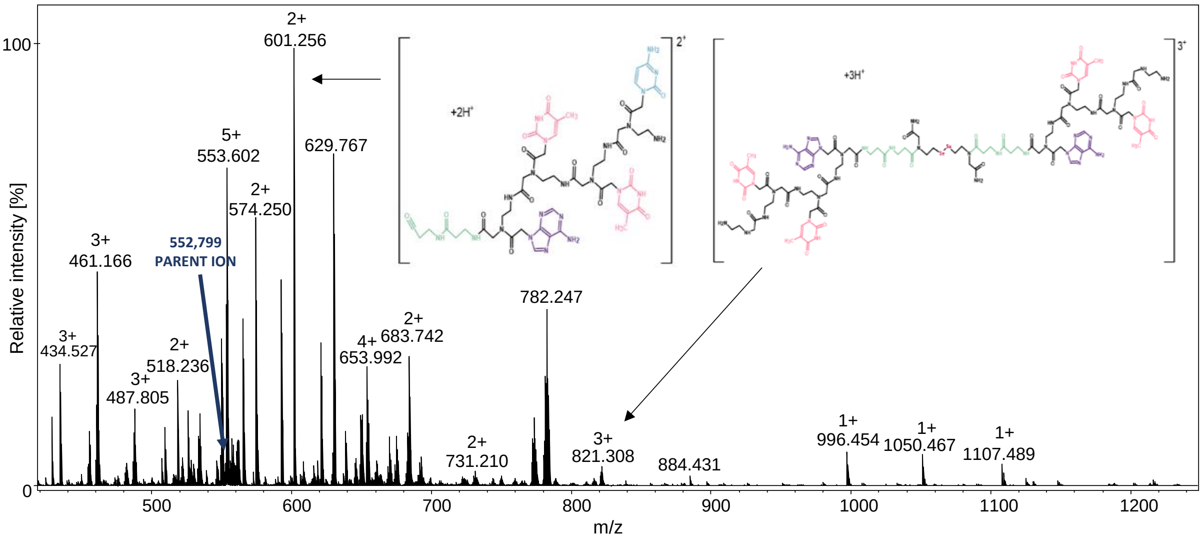


*Fig. S 77 ESI-MS/MS (CE 25 eV) spectrum obtained for PNA conjugate 6.2*

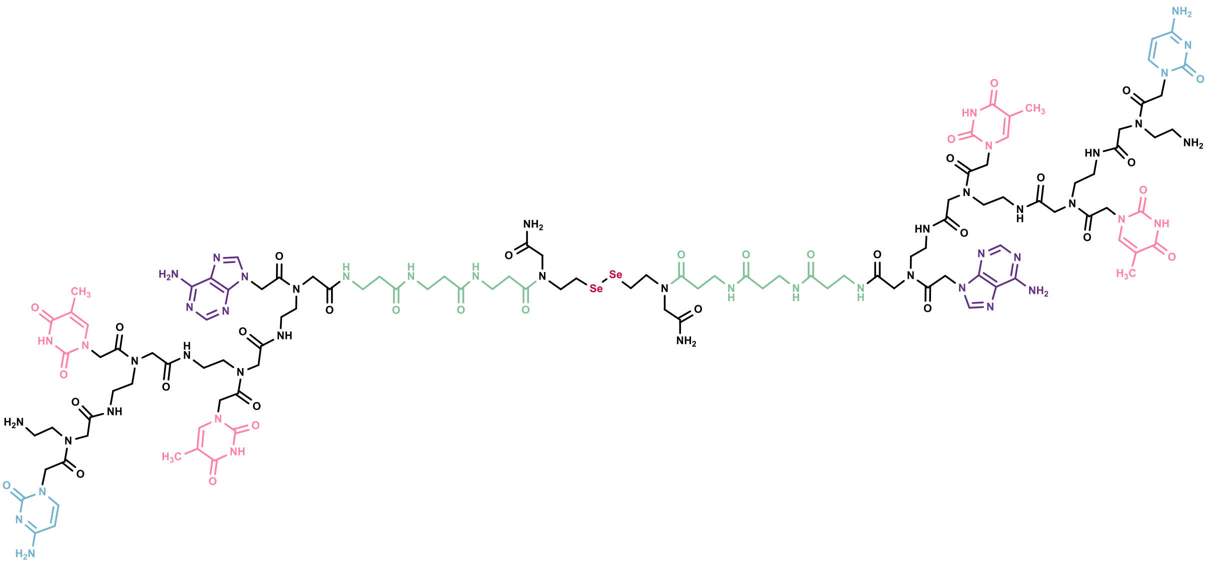

*Fig. S 78 Structure of PNA conjugate 7.2*

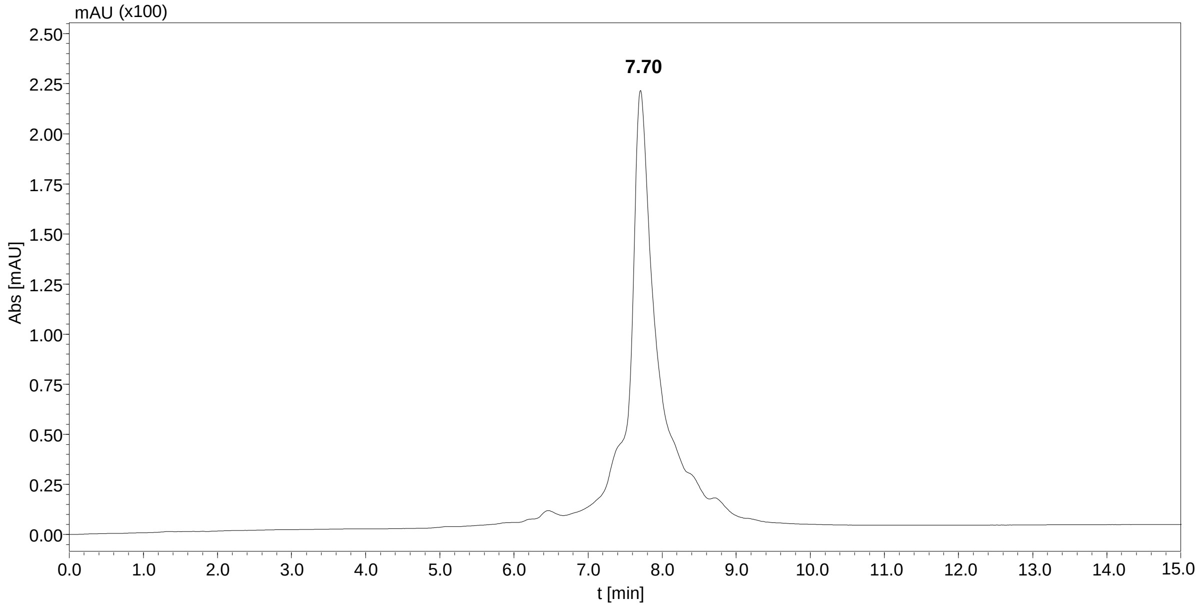


*Fig. S 79 HPLC chromatogram obtained for PNA conjugate 7.2*

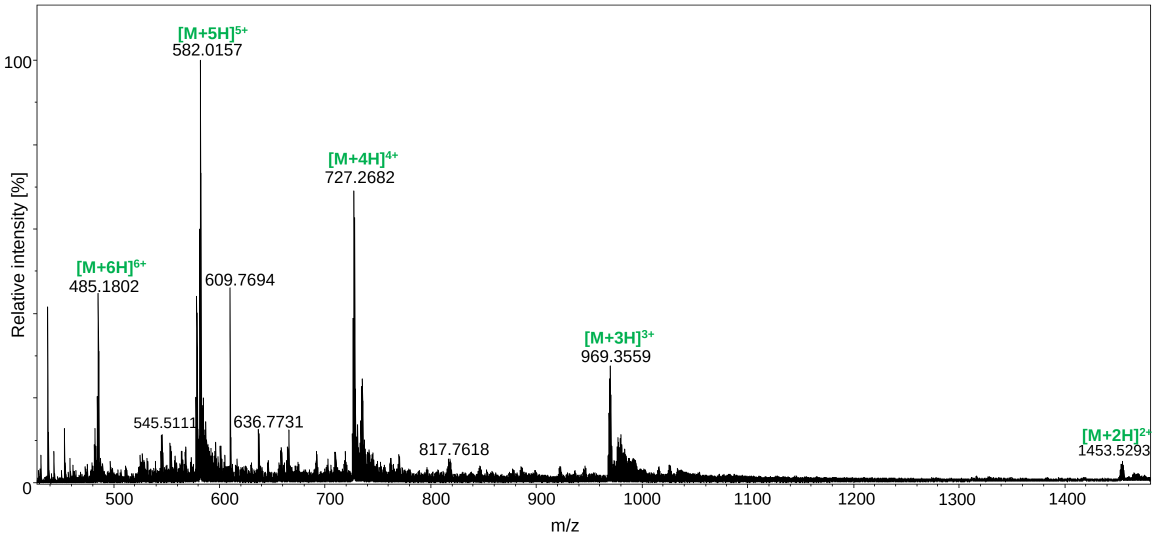


*Fig. S 80 ESI-MS spectrum obtained for PNA conjugate 7.2*

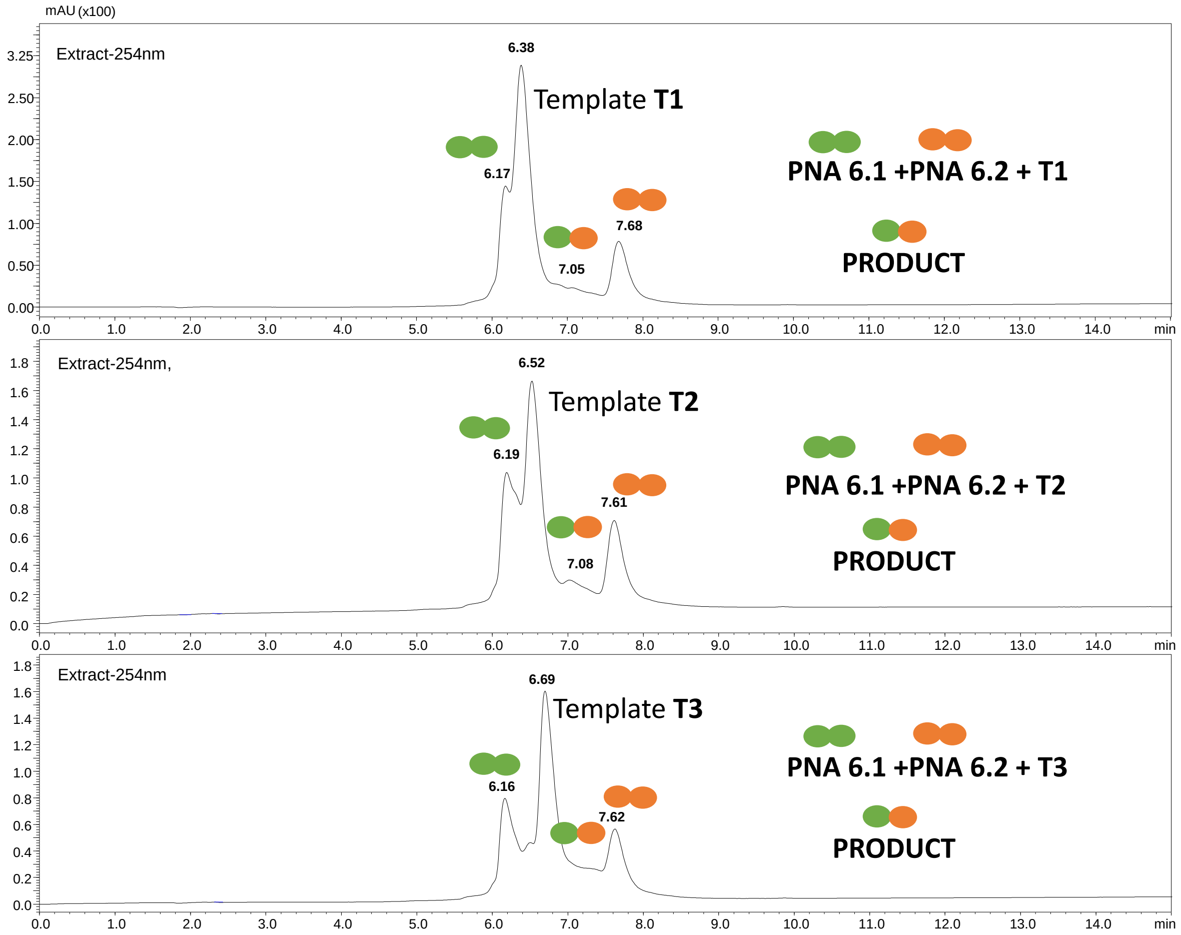


*Fig. S 81 HPLC chromatograms obtained for 3-component mixtures consisting of PNA conjugate 6.1, 6.2 and PNA template T1, T2 and T3, respectively, after visible-light irradiation for 60 min*

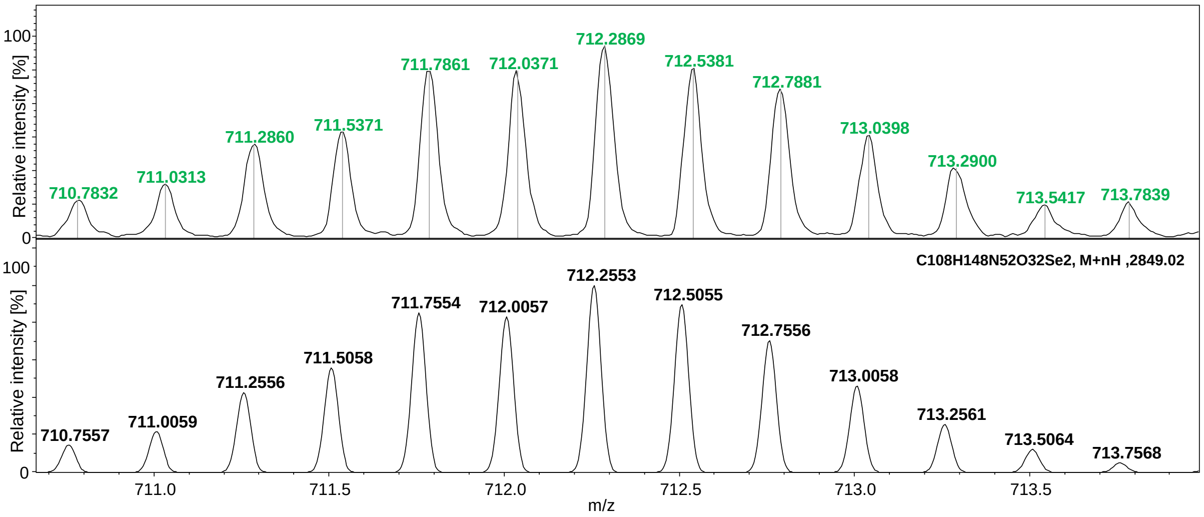


*Fig. S 82 MS spectrum (expanded area) obtained for 3-component mixtures consisting of PNA conjugate 6.1, 6.2 and PNA template, T2 after visible-light irradiation for 60 min. Expanded isotopic pattern for the signal corresponding to the metathesis product and its comparison with the simulated one (charge 4+)*

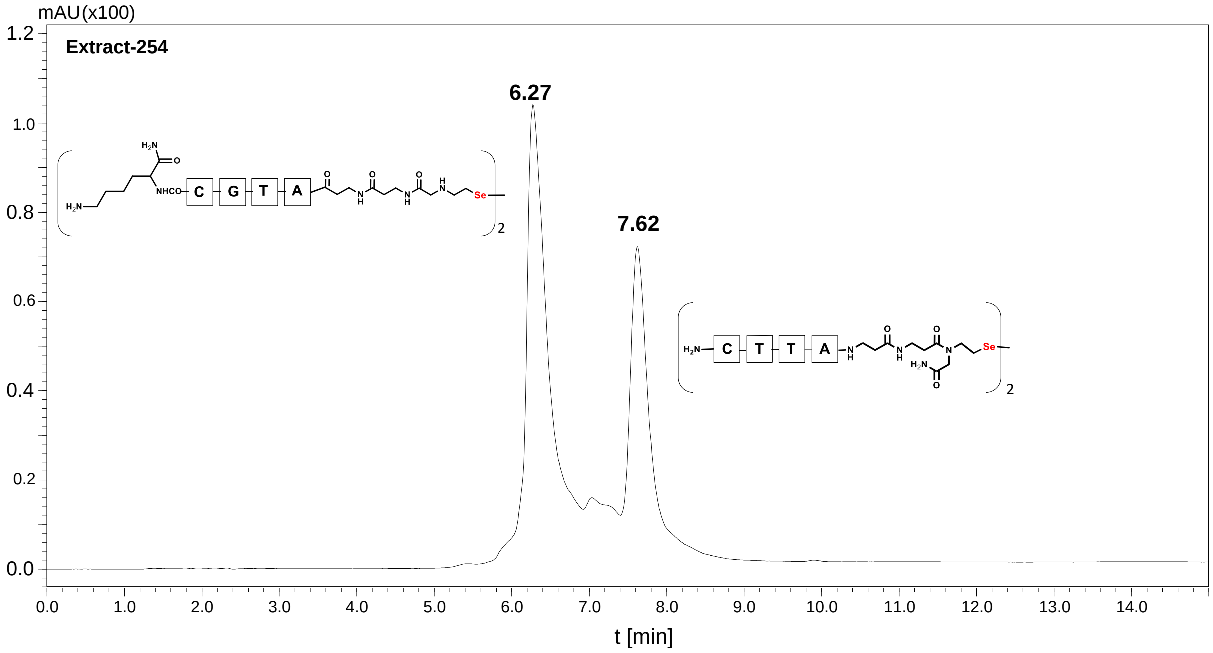


*Fig. S 83. HPLC chromatogram obtained after irradiation of non-complemenatry PNA conjugates*

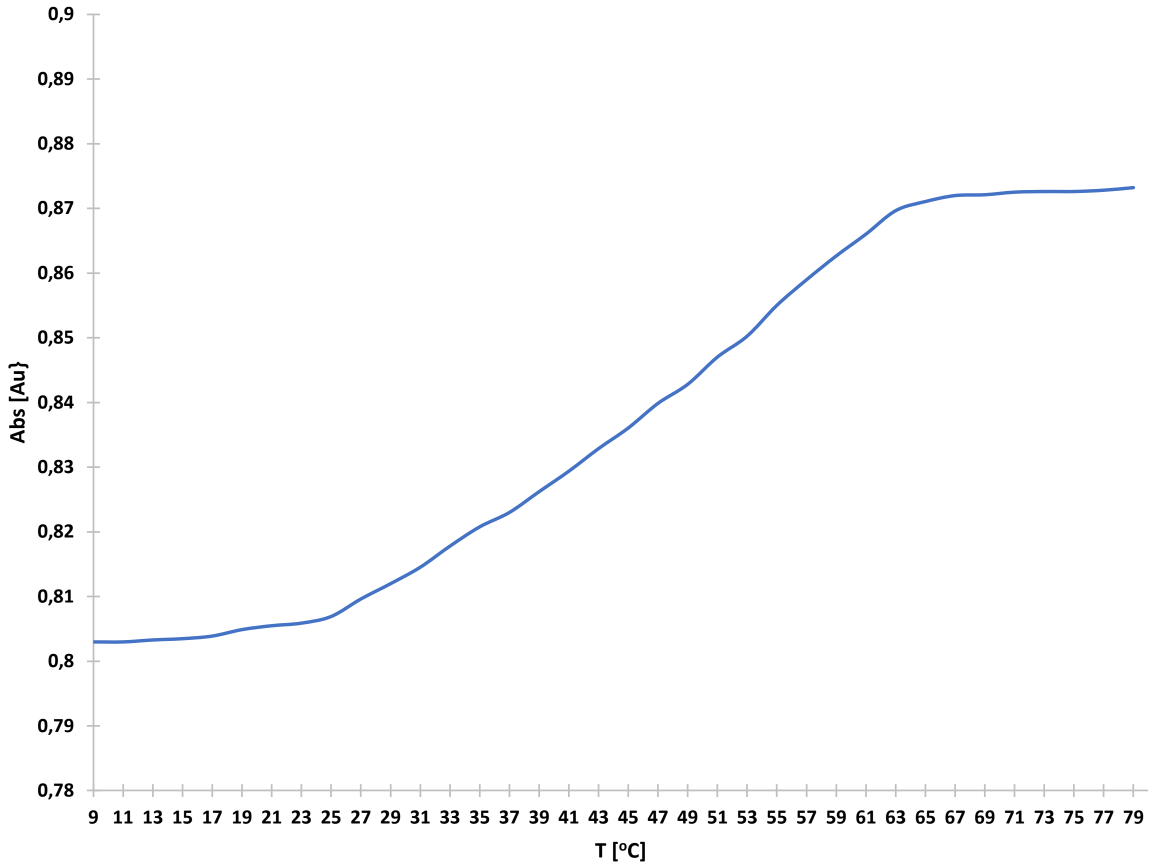


*Fig. S 84 Melting curve obtained for metathesis product after irradiation of PNA 3.1 and 3.2*

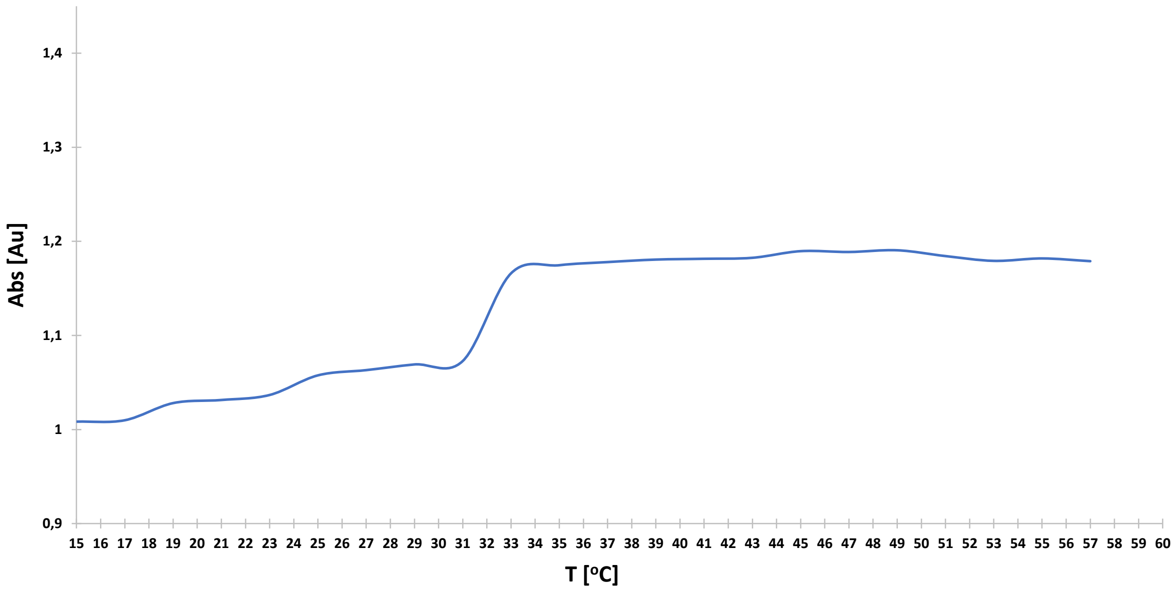


*Fig. S 85 Melting curve obtained for 3-component system consisting of template 2, PNA 6.1 and 6.2*
